# Highly efficient hydrogenative depolymerisation of polycaprolactone to 1,6-hexanediol

**DOI:** 10.1039/d5su00729a

**Published:** 2025-12-02

**Authors:** Garima Saini, Alejandra Sophia Lozano Perez, Niklas von Wolff, Amit Kumar

**Affiliations:** a EaStCHEM, School of Chemistry, University of St. Andrews St. Andrews KY169ST UK ak336@st-andrews.ac.uk; b Institut Parisien de Chimie Moléculaire (IPCM), UMR 8232, Sorbonne Université, CNRS 75005 Paris France

## Abstract

We report here our study on the development of an efficient process to make 1,6-hexanediol from the hydrogenation of polycaprolactone assisted by ethanolysis. Using a ruthenium SNS pincer catalyst, a record high turnover number of 19 600 with 98% yield of 1,6-hexanediol is obtained at 80 °C and 60 bar H_2_ pressure. The reported method has environmental advantages over the conventional process for the production of 1,6-hexanediol, which emits a significant amount of nitrous oxide greenhouse gas.

## Abstract 2

We report here our study on the development of an efficient process to make 1,6-hexanediol from the hydrogenation of polycaprolactone assisted by ethanolysis.

## Graphical abstract

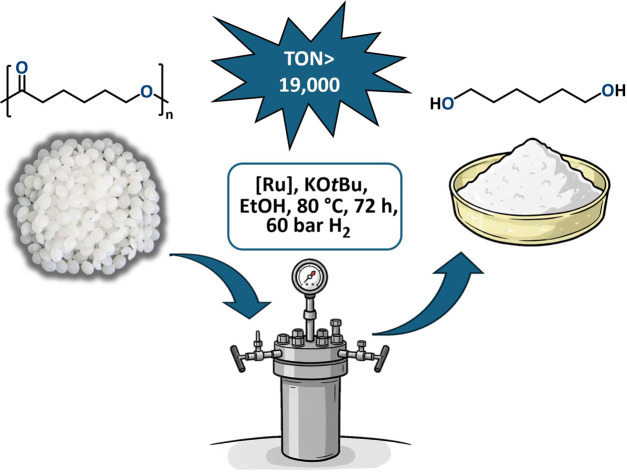

**Sustainability spotlight**

Plastic waste poses a growing environmental challenge, and even “biodegradable” plastics like polycaprolactone can persist for years, releasing CO_2_ as they degrade. This work demonstrates a highly efficient catalytic process to upcycle PCL waste through ruthenium catalysed hydrogenative depolymerisation producing 1,6-hexanediol, a valuable feedstock for polyurethanes and polyesters. By achieving high turnover numbers up to 19 600 and yields up to 98% in ethanol (80 °C), the method provides a greener alternative to conventional industrial routes that emit nitrous oxide, a potent greenhouse gas. This sustainable advancement contributes to circular economy strategies by converting plastic waste into useful chemicals, directly supporting UN Sustainable Development Goals 9 (Industry, Innovation, and Infrastructure), 12 (Responsible Consumption and Production), and 13 (Climate Action).

## Introduction

**Fig. 1:**
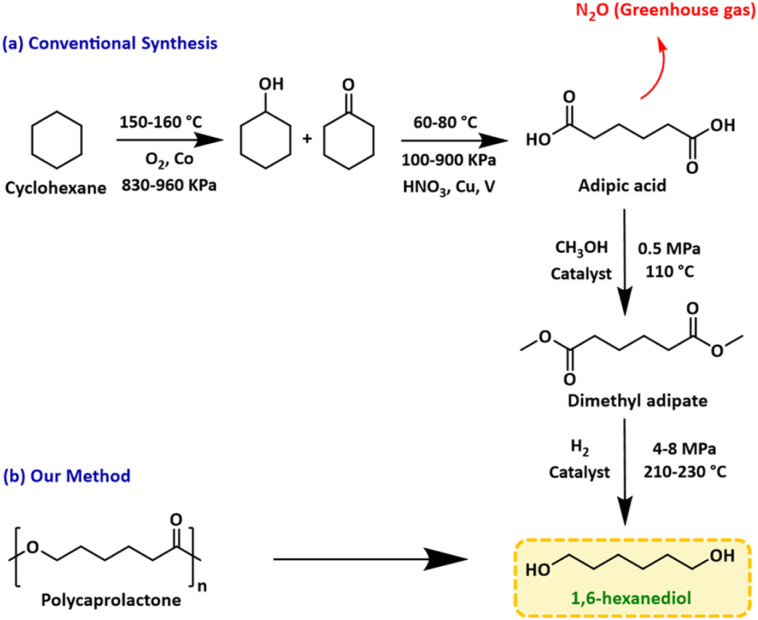
Industrial method (a) and hydrogenative route (b) for the synthesis of 1,6-HD.

1,6-Hexanediol (1,6-HD) is a versatile compound with a current market size of >£1 billion and is used in the production of polyesters,^1^ polyurethanes,^2^ and adhesives.^3^ Industrially, 1,6-HD is produced from the catalytic hydrogenation of dimethyl adipate, which is produced from adipic acid (Fig. 1A). The production of adipic acid has serious environmental concerns, mainly due to the need for large quantities of nitric acid, leading to significant nitrous oxide (NO_*x*_) emission (Fig. 1), which has a 300-fold higher global warming potential than CO_2_.^4^ Although NO_*x*_ abatement technologies have been developed *e.g.* catalytic destruction, thermal decomposition, and recycling for nitric acid production, they are energy intensive and require further downstream treatments.^5^ Thus, the development of new and efficient routes to produce 1,6-HD, avoiding NO_*x*_ emission, will be beneficial to the environment.

A few alternative routes for the production of 1,6-HD or adipic acid have been developed lately. They involve the use of alternative oxidants such as H_2_O_2_ or O_2_, biocatalytic processes,^6^ or alternative feedstock such as butadiene.^5^ Bioderived feedstock such as 5-hydroxymethylfurfural has also been utilised for the direct synthesis of 1,6-HD through a transfer hydrogenation process using heterogeneous catalysts.^7,8^

In the last two decades, significant attention has been given to the use of plastic waste as a resource for the production of valuable feedstock. In particular, we realised that 1,6-HD can be produced from the direct hydrogenation of polycaprolactone (PCL, Fig. 1B). Polycaprolactone is a versatile plastic with a current global market value >£360 million per year^9^ and widespread applications in 3D printing, packaging and biomedical engineering.^10–14^ Despite its classification as a biodegradable polymer, the environmental degradation of PCL can take 2–3 years.^15,16^ Moreover, the process results in the release of CO_2_, a greenhouse gas that contributes to climate change. Thus, the use of PCL waste to make 1,6-HD would be more beneficial to the circular economy than allowing it to biodegrade in nature.

**Fig. 2:**
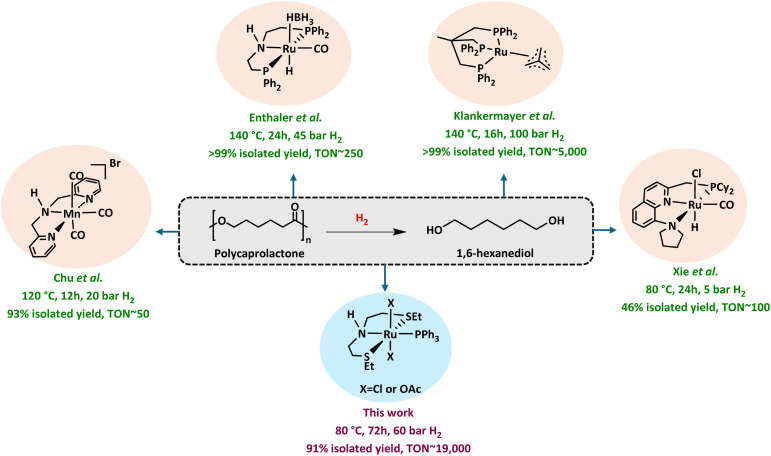
Reported studies on the hydrogenation of PCL and the work reported herein.

Among several methods studied for the chemical recycling of PCL,^17–25^ the approach of catalytic hydrogenation is the most relevant here as it offers its direct transformation to 1,6-HD. Indeed, hydrogenative depolymerisation of PCL to 1,6-HD has been reported in the past (Fig. 2) demonstrating a TON of <50 (using Manganese pincer catalysts at 120–150 °C in THF by Chu *et al.*),^26,27^ 250 (using the Ru-MACHO-BH catalyst at 140 °C in toluene by Enthaler *et al.*),^28^ and 5000 (using Ru-Triphos/HNTf_2_ at 140 °C in 1,4-dioxane by Klankermayer *et al.*).^29^ Xie *et al.* developed a relay process for polyester depolymerisation in methanol/toluene, where the polymer first undergoes transesterification to produce oligomers, followed by hydrogenation to diols using a Ru-pincer catalyst.^30^ This reaction was effective under mild conditions and showed TON ∼100 for the hydrogenation of PCL to 1,6-HD. Although these results are promising, for practical purposes, more efficient catalytic processes (*e.g.* higher TON) will be desirable. We present here our study on the catalytic hydrogenative depolymerisation of PCL to selectively produce 1,6-HD in excellent yields with a TON up to 19 600.

## Results and discussion

We began our investigation by studying the hydrogenation of a commercial polycaprolactone (*M*_n_ = 80 000 Da) in the presence of various transition-metal complexes known for the hydrogenation of esters.^31–34^ Similar to Xie's report, we envisioned that performing the reaction in the presence of ethanol could allow faster depolymerisation of PCL *via* ethanolysis, enabling us to achieve higher TON for the hydrogenation process.^30^ PCL pellets (1 mmol relative to the monomer) were subjected to hydrogenation in 1 mL 2-MeTHF using 0.05 mol% of precatalyst, 10 mol% KO*t*Bu, and 0.1 mL EtOH at 80 °C for 20 h under 60 bar H_2_ pressure. Analysis of the reaction mixture by NMR spectroscopy and GC-MS at the end of reaction time showed the formation of a mixture of products — 1,6-hexanediol (1,6-HD), ethyl 6-hydroxy hexanoate (E6-HH) and ε-caprolactone (ε-CPL) when complexes 1–6 were used as precatalysts. Analysis of the reaction mixture by GPC also showed the presence of small oligomers of *M*_n_ = ∼300 Da. Interestingly, the use of complexes 7 (ref. 34) and 8 (ref. 35–37) under the same conditions led to excellent selectivity towards 1,6-hexanediol, achieving yields of 87% (entry 7, Table 1) and 84% (entry 8, Table 1). Increasing the amount of 2-MeTHF and ethanol to 2 mL and 0.25 mL, respectively, further increased the yield of 1,6-HD to 93% (entry 9) and >99% (entry 10) with a turnover number (TON) of 2000 (entry 10). Precatalysts 7 and 8 have been reported to demonstrate excellent TON (*e.g.* >50 000) for small molecule ester hydrogenation and therefore trends observed here are in line with their previously reported catalytic activity.^34,35^

**Table 1:** Optimisation of catalytic conditions for the hydrogenation of polycaprolactone^a^

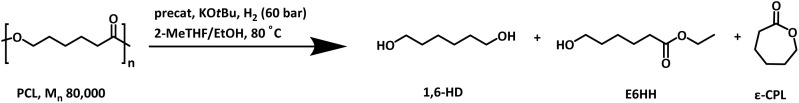											
Entry	Precatalyst	Precatalyst loading (mol%)	Base	Base loading (mol%)	2-MeTHF (mL)	EtOH (mL)	Time (h)	1,6-HD (%)	E6-HH (%)	ε-CPL (%)	TON^b^
1	1	0.05	KO*t*Bu	10	1	0.1	20	54	10	0	1080
2	2	0.05	KO*t*Bu	10	1	0.1	20	0	39	1	0
3	3	0.05	KO*t*Bu	10	1	0.1	20	0	44	1	0
4	4	0.05	KO*t*Bu	10	1	0.1	20	0	51	0	0
5	5	0.05	KO*t*Bu	10	1	0.1	20	42	17	0	840
6	6	0.05	KO*t*Bu	10	1	0.1	20	17	29	1	340
7	7	0.05	KO*t*Bu	10	1	0.1	20	87	0	0	1740
8	8	0.05	KO*t*Bu	10	1	0.1	20	84	0	0	1680
9	7	0.05	KO*t*Bu	10	2	0.25	20	93	0	0	1860
10	8	0.05	KO*t*Bu	10	2	0.25	20	>99	0	0	2000
11	8	0.02	KO*t*Bu	10	2	0.25	20	36	40	2	1800
12	8	0.01	KO*t*Bu	10	2	0.25	20	34	40	2	3400
13	—	—	KO*t*Bu	10	2	0.25	20	0	48	10	0
14^c^	8	0.05	KO*t*Bu	10	2	0.25	20	88	0	0	1760
15	8	0.05	KO*t*Bu	—	2	0.25	20	0	0	0	0
16	8	0.05	KO*t*Bu	2	2	0.25	20	12	52	1	240
17	8	0.05	KO*t*Bu	5	2	0.25	20	>99	0	0	2000
18	8	0.05	KOH	5	2	0.25	20	93	0	0	1860
19	8	0.05	NaO*t*Bu	5	2	0.25	20	96	0	0	1920
20^d^	8	0.05	KO*t*Bu	5	2	0.25	20	25	39	1	500
21^e^	8	0.05	KO*t*Bu	5	2	0.25	20	87	0	0	1740
22^f^	8	0.05	KO*t*Bu	5	2	0.25	20	0	50	8	0
23^g^	8	0.05	KO*t*Bu	5	2	0.25	20	84	0	0	1680
24	8	0.05	KO*t*Bu	5	2	—	20	13^h^	0	0	260
25	8	0.05	KO*t*Bu	5	2	0.1	20	87	0	0	1740
26	8	0.01	KO*t*Bu	5	2	0.25	72	92	0	0	9200
27	8	0.005	KO*t*Bu	5	2	0.25	72	79	6	0	15 800
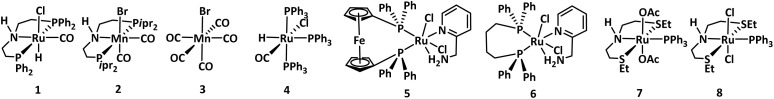											

a Polycaprolactone, *M*_n_ 80 000 (1 mmol of monomeric unit), 2-Me THF, EtOH, KO*t*Bu (1 M solution in THF), precatalyst (2 mM solution in 2-Me THF), 80 °C, 60 bar H_2_. Yields were estimated by quantitative GC-MS analysis using mesitylene as an internal standard.

b TON estimated based on the number of ester groups hydrogenated.

c Reaction performed at 60 °C.

d THF used as the solvent.

e Toluene used as the solvent.

f No external hydrogen pressure.

g 30 bar H_2_ pressure.

h ^1^H NMR yield.

We performed further optimisation studies using complex 8, aiming to achieve higher turnover numbers. Lowering the catalytic loading of complex 8 to 0.02 mol% and 0.01 mol% (keeping the remaining conditions the same) produced a mixture of products with only 36% (entry 11) and 34% (entry 12) of 1,6-HD, respectively, in 20 h. A control reaction conducted without any metal–complex formed E6-HH (48%) and ε-CPL (10%), and the formation of 1,6-hexanediol was not observed (entry 13). This highlights the essential role of the metal complex in the hydrogenation process. Reducing the reaction temperature to 60 °C from 80 °C reduced the yield of 1,6-HD slightly (88%, entry 14, in comparison to >99%, entry 10).

We also studied the effect of a base and found that the reaction did not work in the absence of a base, likely because the base is needed to generate the active species (entry 15).

Performing the reaction using 2% of KO*t*Bu showed poor selectivity of the reaction (entry 16), whereas 5% KO*t*Bu led to the complete conversion of PCL, forming 1,6-HD in >99% yield (entry 17), similar to when 10% base was used (entry 10), suggesting that 5% base is sufficient for this process. We then carried out reactions with different bases to understand the effect of the nature of the base. The use of 5% KOH (entry 18) or 5% NaO*t*Bu (entry 19) instead of 5% KO*t*Bu (entry 17), keeping the remaining conditions the same, resulted in similar yields of 1,6-HD. Next, we examined the effect of solvent under the reaction conditions described in entry 17. Surprisingly, the use of THF instead of 2-Me THF in combination with EtOH resulted in only 25% yield of 1,6-HD (entry 20), whereas 87% yield of 1,6-HD was obtained in the case of toluene + EtOH (entry 21, Table 1). In a control experiment, the hydrogenation of PCL to 1,6-HD was not observed when the reaction was carried out in the absence of H_2_ (entry 22), thus ruling out the possibility of transfer hydrogenation. Reducing the H_2_ pressure from 60 bar (entry 15) to 30 bar (entry 23) lowered the yield from >99% to 84%. Performing the reaction without ethanol (entry 24) yielded only 13% 1,6-HD, and lowering its amount to 0.1 mL (entry 25) gave a lower yield (87%) than that of entry 17 (>99%). This signifies the crucial role of ethanol in achieving higher yields of 1,6-HD and catalytic TON.

Moving further, we conducted the reactions for a longer duration (72 hours), aiming to obtain higher yields under lower catalytic loadings. Remarkably, conducting a reaction at 0.01 mol% of 8 with 5 mol% KO*t*Bu for 72 h (entry 26) led to the formation of 1,6-HD in 92% yield, exhibiting a TON of 9200. Further lowering the catalytic loading to 0.005% (entry 27, Table 1) led to the formation of 1,6-HD in 79% yield, demonstrating a record high TON of ∼16 000 for the hydrogenative depolymerisation of PCL using a homogeneous catalyst.

**Fig. 3:**
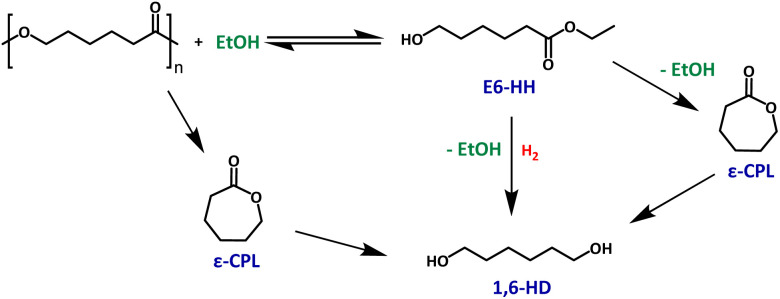
Proposed mechanism for the hydrogenative degradation of PCL.

To develop a mechanistic hypothesis, we propose that PCL first undergoes transesterification with ethanol to form E6-HH or depolymerizes to ε-CPL. These intermediates are then hydrogenated to yield 1,6-HD (Fig. 3).

**Fig. 4:**
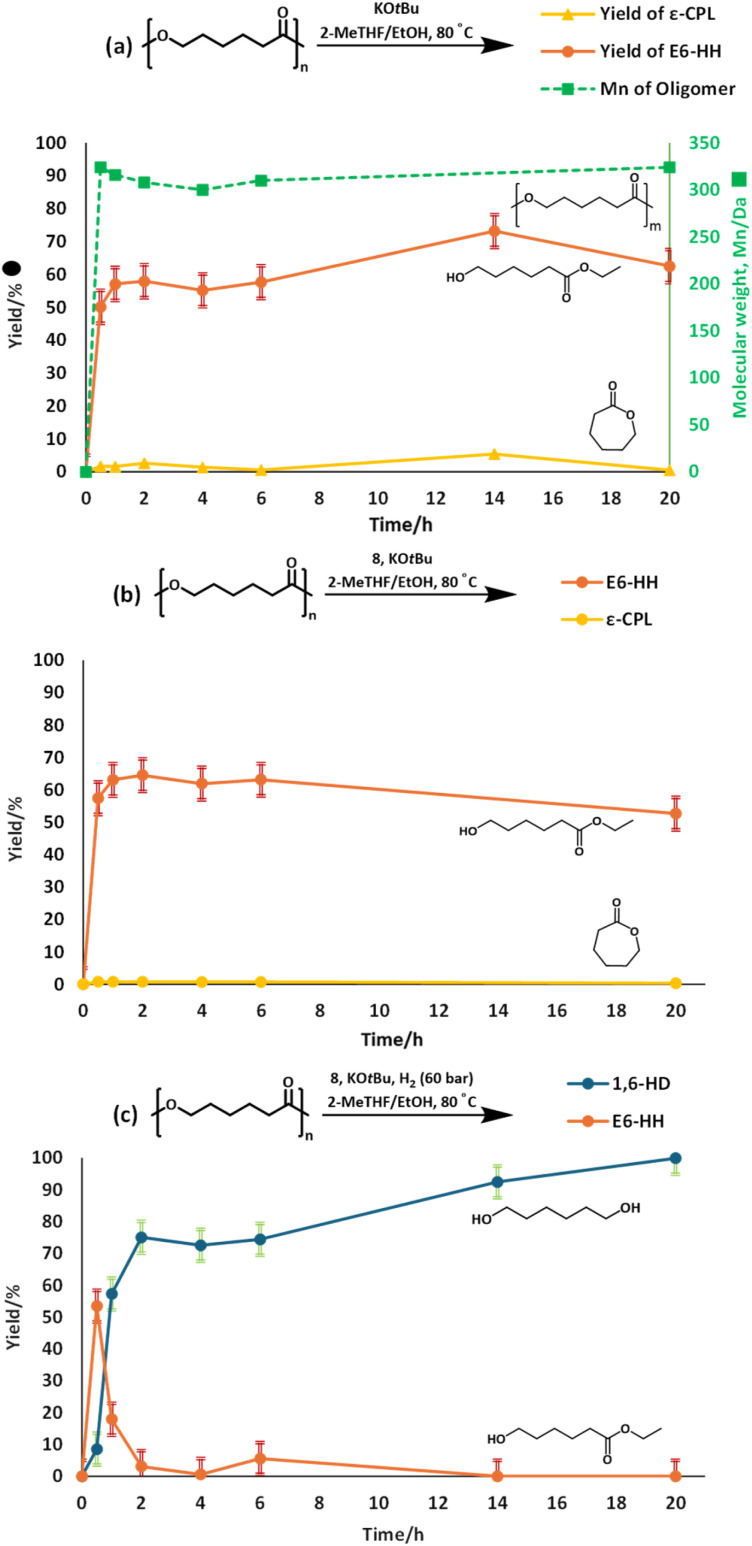
Temporal profile of the depolymerisation of PCL without hydrogen and without a metal complex, in the presence of KO*t*Bu (a) without hydrogen but in the presence of complex 8 and KO*t*Bu (b) and in the presence of H_2_, complex 8 and KO*t*Bu (c) with an error of ±5%. Conditions as per Table 1, entry 17.

To gain more insights, we studied the temporal profile of the depolymerisation of PCL. Performing a reaction of PCL without H_2_ pressure and without a ruthenium precatalyst (Fig. 4a), keeping the other conditions the same as those in entry 17, Table 1, led to the formation of E6-HH and ε-CPL in ∼50% and ∼2% yields, respectively, in 30 min. The yield of E6-HH increased slightly to 58% in 2 h and remained nearly unchanged until 20 h. Similarly, the yield of ε-CPL remained within 1–2% over 20 h. The GPC analysis at these points revealed the presence of oligomers of *M*_n_ ∼ 300 Da. These experiments are suggestive of an equilibrium between E6-HH and oligomers of *M*_n_ = ∼300 Da. Performing the transesterification reaction with methanol and isopropanol led to the formation of methyl 6-hydroxyhexanoate and isopropyl 6-hydroxyhexanoate in 76% and 52% yields, respectively, suggesting that transesterification can occur using various aliphatic alcohols. Another reaction carried out with precatalyst 8 and KO*t*Bu (entry 17, Table 1), but in the absence of hydrogen pressure (Fig. 4b), showed results similar to those in Fig. 4a, suggesting that ruthenium does not influence the ethanolysis process, and that the ethanolysis process is catalyzed solely by the base. However, when the reaction was conducted in the presence of H_2_ pressure using precatalyst 8, and KO*t*Bu (entry 17, Table 1), the yield of E6-HH increased for 30 min and started decreasing with time (Fig. 4c). On the other hand, the yield of 1,6-HD increased drastically for 2 h and then slowly reached 99% in 20 h. Almost quantitative yield of 1,6-HD and 0% yield of E6-HH after 20 h support our proposed pathway, where 1,6-HD forms through the hydrogenation of E6-HH, driving the equilibrium towards the product side. Under our optimised conditions, ε-CPL was also fully hydrogenated to 1,6-HD, confirming the possibility of ε-CPL being an intermediate (Fig. S54, see SI).

**Fig. 5:**
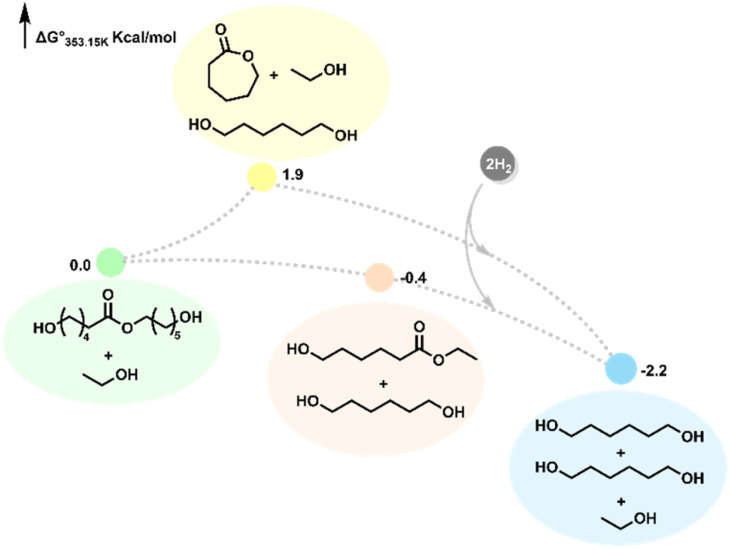
Free energy for the depolymerisation of the caprolactone dimer to form 1,6-HD. Free energies were calculated at 353.15 K, 60 bar and at 1 M concentration of solutes in kcal mol^−1^ (see the SI for details).

To further probe into the equilibrium, we estimated the thermodynamics of the process using DFT computation at the B3LYP/6-21G//B3LYP/6-311G+(d,p) level of theory. The Δ*G* for the ethanolysis of a PCL dimer to E6-HH and 1,6-HD was found to be downhill by 0.4 kcal/mol, suggesting the process to be reversible and supporting our proposal of an equilibrium between E6-HH and oligomers based on Fig. 4A. Thermodynamically, the cyclisation to caprolactone was found to be uphill by 1.9 kcal mol^−1^, whereas the hydrogenation to make 1,6-HD was found to be downhill by 2.2 kcal mol^−1^, in line with our observation of high yield of 1,6-HD in the presence of hydrogen gas (Fig. 5).

To make the catalytic process more efficient, we conducted further optimisation of the reaction conditions with a minimum amount of 2-MeTHF needed to make a clear stock solution of the precatalyst 8, as it is not soluble in ethanol. Remarkably, without using any additional solvent, 91% yield of 1,6-HD was obtained using 0.05% of 8 in 20 h. Lowering the catalytic loadings to 0.01% and 0.005% led to the formation of 96% (TON = 9,600, entry 2) and 88% (TON = 17 600, entry 3) of 1,6-HD in 72 h.

To completely eliminate the need for 2-MeTHF, we tested the catalytic activity using precatalyst 7, which has a higher solubility in ethanol in comparison to that of 8 and thus can be used to make a stock solution. Similar to precatalyst 8, 96% of 1,6-HD was obtained when 0.05 mol% of 7 was used (entry 4, Table 2). However, the reaction performed better with precatalyst 7 under lower catalytic loading. The hydrogenation of PCL using 0.01% and 0.005% catalytic loading of 7 yielded 99% (entry 5, Table 2) and 98% (entry 6, Table 2) of 1,6-HD selectively, reaching TON near 10 000 and 20 000, even better than those obtained in entries 26 and 27 in Table 1. We also scaled up the process, successfully hydrogenating 5 mmol of PCL to 1,6-HD under the optimized reaction conditions, affording yields of about 92% (TON = 18 400) and 95% (TON = 19 000) with precatalysts 8 and 7, respectively (entries 7 and 8, Table 2). 1,6-HD was also isolated in 91% yield from the reaction conducted in entry 8 through simple filtration and solvent extraction, followed by vacuum drying (see SI, Section 3).

**Table 2:** Hydrogenation of polycaprolactone under solvent-minimised conditions^a^

Entry	Precatalyst	Precatalyst loading (mol%)	KO*t*Bu (mol%)	EtOH (mL)	Time (h)	1,6-HD (%)	E6-HH (%)	ε-CPL (%)	TON^b^
1	8	0.05	5	0.25	20	91	0	0	1820
2	8	0.01	5	0.25	72	96	0	0	9600
3	8	0.005	5	0.25	72	88	0	0	17 600
4	7	0.05	5	0.25	20	96	0	0	1920
5	7	0.01	5	0.25	72	99	0	0	9900
6	7	0.005	5	0.25	72	98	0	0	19 600
7^c^	8	0.005	5	1.25	72	92	0	0	18 400
8^c^	7	0.005	5	1.25	72	95	0	0	19 000

a Polycaprolactone *M*_n_ 80 000 (1 mmol of monomeric unit), EtOH, KO*t*Bu, precatalyst 8 (2.5 mM solution in 2-Me THF), precatalyst 7 (2 mM solution in EtOH), 80 °C, 60 bar H_2_. Yields were estimated by quantitive GC-MS analysis using mesitylene as an internal standard.

b TON estimated based on the number of ester groups hydrogenated.

c 5 mmol of PCL used (relative to the monomeric unit).

**Fig. 6:**
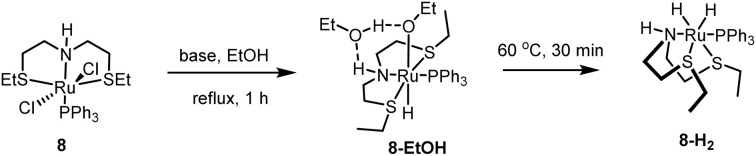
Conversion of complex 8 to facial dihydride complex 8-H_2_.

The mechanism for the hydrogenation of esters using the precatalyst 8 has been reported using both experimental studies and DFT computation.^35,37^ Gusev reported that complex 8, in the presence of a base and ethanol, can form a ruthenium hydride ethoxide complex 8-EtOH that can convert to facial–dihydride complex 8-H_2_ upon heating at 60 °C for 30 minutes.^35^ Yang, through a DFT computation, has reported that complex 8-H_2_ can isomerise to a meridional dihydride complex that performs the hydrogenation of esters.^37^ Based on these studies, it is likely that organometallic catalysis proceeds through a similar pathway where the facial–dihydride complex 8-H_2_ and the corresponding meridional isomer transfer a hydride from the metal centre to the carbonyl group to reduce esters to alcohols (Fig. 6).

## Conclusion

In conclusion, we report here an efficient method of forming 1,6-hexanediol from the hydrogenation of commercial-grade polycaprolactone. A record high turnover number of around 19 000 was achieved using Gusev's catalysts-Ru-SNS-Cl_2_ (complex 8) and Ru-SNS-(OAc)_2_ (complex 7) under relatively mild reaction conditions (80 °C, and 60 bar H_2_), producing 1,6-HD in up to 98% yield. Based on our mechanistic studies, we suggest that the reaction undergoes a relay process where PCL first gets depolymerised to E6-HH through ethanolysis, followed by the hydrogenation to make 1,6-HD in the presence of H_2_ pressure.

## Conflicts of interest

There are no conflicts to declare.

## Supplementary Material

SU-004-D5SU00729A-s001

## Data Availability

All raw data (NMR, GC-MS, and DFT computations) files supporting this publication can be accessed at https://doi.org/10.17630/6a910f9b-befc-4b74-81a7-6c6cfb08741e. Supplementary information (SI): contains all relevant data and experimental details necessary for conducting catalytic and mechanistic studies. See DOI: https://doi.org/10.1039/d5su00729a.
