# Supplementary material for: Highly efficient hydrogenative depolymerisation of polycaprolactone to 1,6-hexanediol
Source: RSC Sustain. 2025 Dec 2;4(2):829–35. doi: 10.1039/d5su00729a (PMC12709232; doi:10.1039/d5su00729a)
Supplement: SU-004-D5SU00729A-s001 [file SU-004-D5SU00729A-s001.pdf]

Supplementary Information (SI) for RSC Sustainability.
This journal is © The Royal Society of Chemistry 2025

Electronic Supporting Information for Manuscript:

Highly efficient Hydrogenative depolymerisation of Polycaprolactone to 1,6-hexanediol

Garima Saini,[a] Alejandra Sophia Lozano Perez,[a] Niklas von-Wolff,[b] and Amit Kumar*[a]

[a] EaStCHEM, School of Chemistry, University of St. Andrews, North Haugh, St. Andrews, KY16 9ST, UK.

[b] Sorbonne Université, CNRS, Institut Parisien de Chimie Moléculaire, IPCM, eMOCA, 4 Place Jussieu, 75005 Paris, France.

Email: ak366@st-andrews.ac.uk

Table of Contents

1. General Considerations 3
2. Procedure for hydrogenolysis of Polycaprolactone 3
3. Method for the isolation of 1,6-hexanediol 4
4. Characterisation of commercial polycaprolactone 4
5. Characterisation of commercial ethyl 6-hydroxyhexanoate 6
6. Characterisation of commercial ε-caprolactone 8
7. Characterisation of isolated product-1,6-hexanediol 9
8. Optimisation of Catalytic Conditions 11
9. Optimisation of Catalytic Conditions under solvent minimised conditions 34
10. Kinetic studies 38
11. Reaction with ε-caprolactone 43
12. Role of alcohols in transesterification reactions 43
13. Role of temperature in transesterification and hydrogenation reactions 45
14. Catalyst stability 46
15. DFT Studies 47
16. References 53

1. General Considerations

All manipulations, unless otherwise stated, were performed under an argon atmosphere using standard Schlenk line and glove-box techniques. Hydrogen gas was supplied by BOC Gases. Glassware was oven-dried at 130 °C overnight and flamed under vacuum prior to use. THF and Toluene were dried using a Grubbs-type solvent purification system (Innovative Technologies SPS) equipped with a degasser. Polycaprolactone (M_n = 80,000 Da), 2-methyltetrahydrofuran (anhydrous) and ethanol (anhydrous) were purchased from Sigma-Aldrich. Afterwards, the solvents were degassed by flushing them with argon through an argon lance equipped with a frit. The solvents were dried and stored in glass Schlenk flask over molecular sieves 3 Å or 4 Å. Ruthenium complex **1** was purchased from TCI and used as received. Manganese complexes **2** and **3** were synthesized following the procedure described in the literature.[1] Complex **4** was bought from Thermo Scientific and complexes **5**, **6**, **7**, and **8** were donated by Johnson Matthey.

KO*t*Bu, KOH, and NaO*t*Bu were purchased from Sigma-Aldrich, and TCI and stored at 80 °C and dried in oven before use. NMR solvents were purchased from Sigma-Aldrich and used as received.

NMR spectra were recorded on a Bruker AVII 500 MHz and Bruker Neo 400 MHz NMR spectrometer at 298 K unless otherwise specified. Residual proton solvent was used as reference for ^{1}H spectra in deuterated solvent samples. All chemical shifts (δ) are quoted in ppm and coupling constants (*J*) in Hz. GC-MS (Gas Chromatography-Mass Spectrometry) data was collected using Agilent 8860 GC system coupled to an Agilent 5977B EI instrument. Helium was used as carrier gas with a column flow of 1.87 mL/min. SH-RTx-1 (100% dimethyl polysiloxane, 30 m) was used as a column. Ion source temperature of 200 ^{o}C, and injection temperature of 330 ^{o}C were used. The oven temperature range was from 50 ^{o}C to 250 ^{o}C (start with 50 ^{o}C, hold for 4.2 minutes, followed by a temperature ramp of 10°C/minute for 15 minutes and stay at 250 ^{o}C until 39 minutes).

2. Procedure for hydrogenolysis of Polycaprolactone

General method for the hydrogenation of Polycaprolactone:

Polycaprolactone (114 mg, 1 mmol relative to the monomer, 1eq.) and KO*t*Bu {11.2 mg, 0.1 mmol, 10 mol%, 0.1 mL (1M solution in THF)} were weighed in an 8 mL glass vial with a stirrer and sealed with a crimp cap using a crimper. The vial was cycled with argon (3 times) with needle at the top and syringe connected to the Schlenk line, and 2-MeTHF (2 mL) and ethanol (0.25 mL) were added by syringe. A 2 mM stock solution of the catalyst was prepared by dissolving 0.01 mmol of precatalyst in 5 mL of 2 Me-THF in another vial under argon atmosphere. 0.25 mL (0.05 mol% of precatalyst) of the precatalytic solution was transferred to the reaction vial by syringe. The vial was placed into a 150 mL autoclave with metal beads to ensure thermal conductivity. The autoclave was purged with argon, sealed,

purged with H_2, and pressurised with H_2 at 60 bar. It was then placed in a preheated oil bath at 80 °C and stirred for 20 h. After the completion of reaction, the autoclave was cooled to room temperature and H_2 gas was carefully vented to atmosphere. 1 mmol of internal standard (mesitylene) was added into the reaction mixture and the yield of the product was estimated by GC-MS quantitative analysis. The method for product isolation has been described in Section 3.

Method for the hydrogenation of Polycaprolactone under solvent-minimised conditions:

Polycaprolactone (114mg, 1mmol, 1eq.) and KO*t*Bu {5.6 mg, 0.05 mmol, 5 mol%, 0.05 mL} were weighed in an 8mL glass vial with a stirrer and sealed with a crimp cap using a crimper. The vial was cycled with argon (3 times) with needle at the top and syringe connected to the Schlenk line, and 0.25 mL of Ethanol was added by syringe. A 2 or 5 mM stock solution of the catalyst was prepared by dissolving 0.01 mmol of precatalyst in 5 or 2 mL of 2 Me-THF (for precatalyst 8) or Ethanol (for precatalyst 7) in another vial under argon atmosphere. The required amount of precatalytic solution was transferred to the reaction vial by syringe. The vial was placed into a 150 mL autoclave with metal beads to ensure thermal conductivity. The autoclave was purged with argon, closed, purged with H_2 and pressurised with H_2 at 60 bar. It was then placed in a preheated oil bath at 80 °C and stirred for 20 h. After the completion of reaction, the autoclave was cooled to room temperature and H_2 gas was carefully vented to atmosphere. 1 mmol of internal standard (Mesitylene) was added into the reaction mixture and the yield of the product was estimated by GC-MS quantitative analysis.

3. Method for the isolation of 1,6-hexanediol

The reaction mixture (Table 2, entry 8) was cooled to room temperature. Ethanol (2-5 mL) was then added to the residue, followed by vacuum filtration to remove any starting material left as the desired product is soluble in ethanol whereas the starting material (polycaprolactone) is insoluble in ethanol. The solution containing the product was concentrated using a rotary evaporator to remove the ethanol. It was then extracted with toluene/water mixture to remove the catalyst. 1,6-hexanediol is soluble in water and the catalyst separated by dissolving in Toluene. The aqueous solution was concentrated using the rotary evaporator. Finally, the pure 1,6-hexanediol was dried under vacuum on a Schlenk line for at least 2 hours, yielding the final product in 91% yield.

4. Characterisation of commercial polycaprolactone

^{1}H NMR (500 MHz, CD_2Cl_2): δH 4.07 (t, -O-**CH$_2$**-CH$_2$-CH$_2$-CH$_2$-CH$_2$-CO-), 2.32 (t, -O-CH$_2$-CH$_2$-CH$_2$-CH$_2$-**CH$_2$**-CO-), 1.66-1.63 (m, -O-CH$_2$-**CH$_2$**-CH$_2$-**CH$_2$**-CH$_2$-CO-), 1.41 (m, -O-CH$_2$-CH$_2$-**CH$_2$**-CH$_2$-CH$_2$-CO-).

^{13}C{^{1}H} NMR (126 MHz, CD_2Cl_2): δC 173.34 (-O-CH$_2$-CH$_2$-CH$_2$-CH$_2$-CH$_2$-**CO**-), 64.02 (-O-**CH$_2$**-CH$_2$-CH$_2$-CH$_2$-CH$_2$-CO-), 34.03 (-O-CH$_2$-CH$_2$-CH$_2$-CH$_2$-**CH$_2$**-CO-), 28.35, 25.49, 24.58 (-O-CH$_2$-**CH$_2$**-**CH$_2$**-**CH$_2$**-CH$_2$-CO-).

The NMR data matches well with the literature.[2]

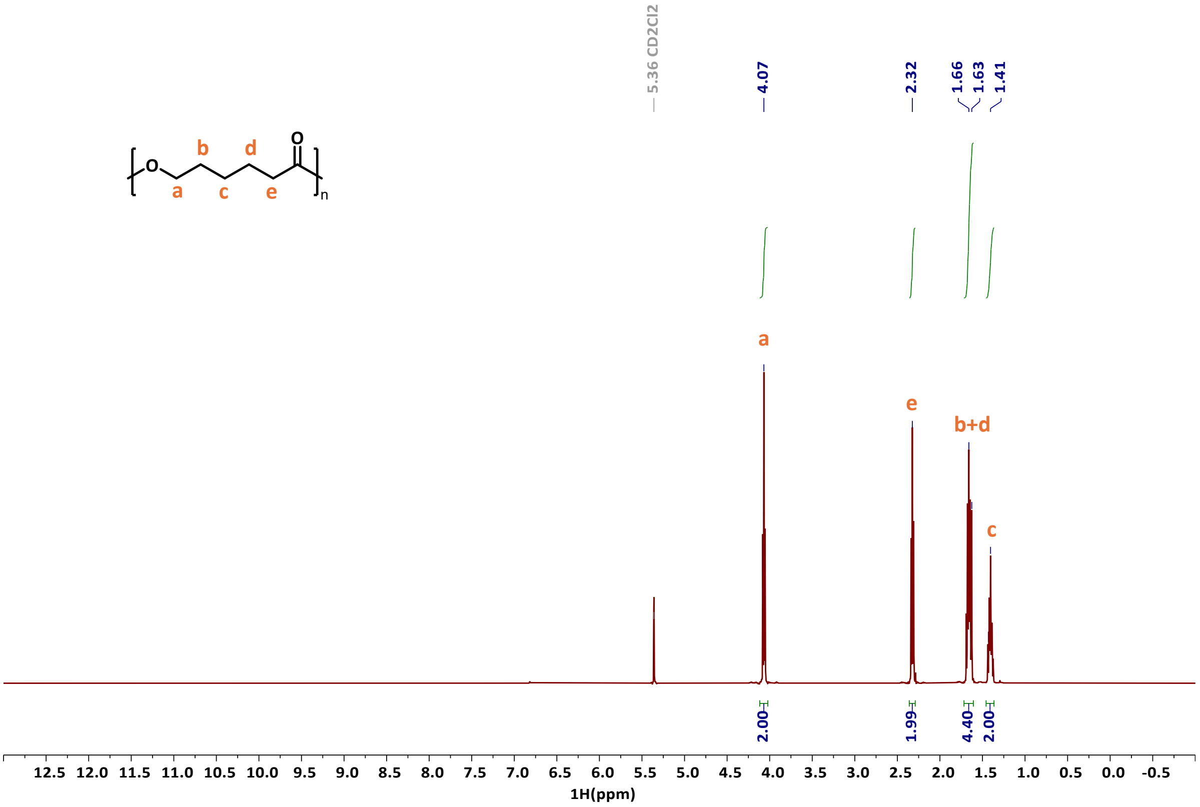

Figure S1. ^{1}H NMR (500 MHz, CD_2Cl_2) spectrum of polycaprolactone.

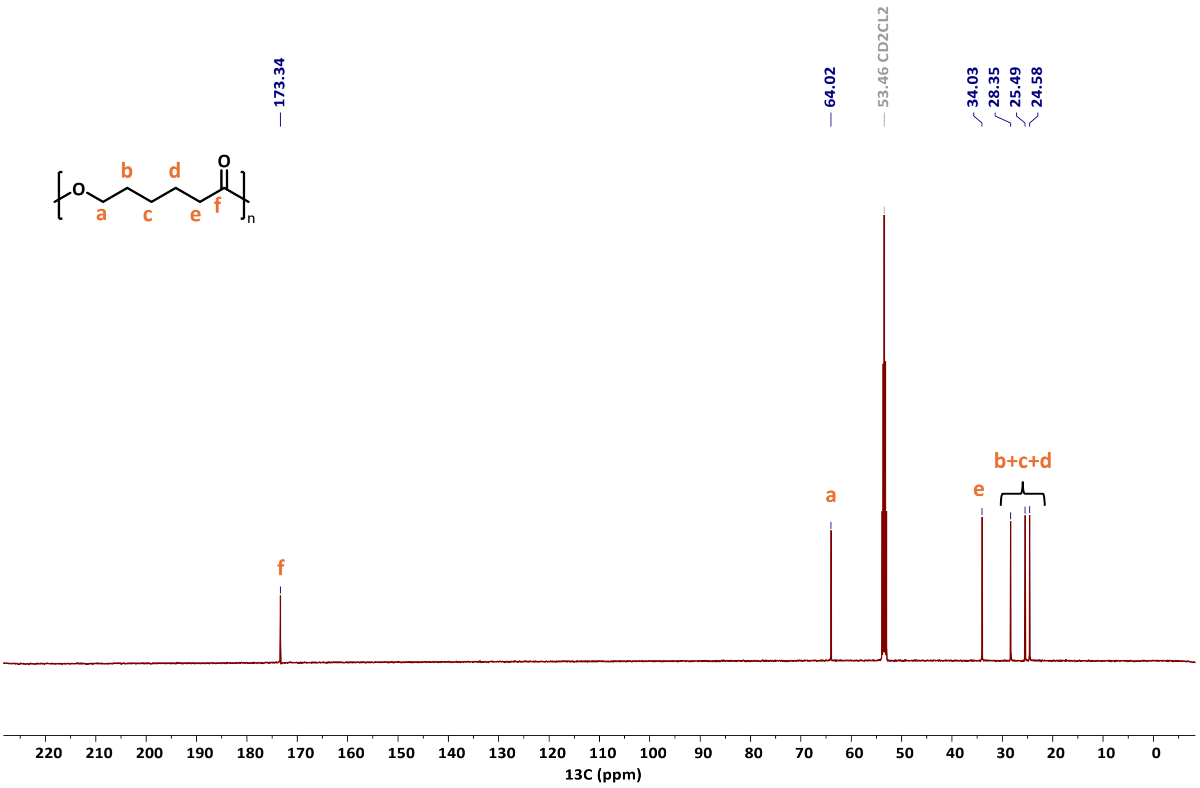

Figure S2. $^{13}C\{^1H\}$ NMR (126 MHz, CD_2Cl_2) spectrum of polycaprolactone.

5. Characterisation of commercial ethyl 6-hydroxyhexanoate

^{1}H NMR (500 MHz, CD_3OD): δH 4.13 (q, HO-CH_2-CH_2-CH_2-CH_2-CH_2-CO-O- **CH_2**-CH_3), 3.57 (t, HO-**CH_2**-CH_2-CH_2-CH_2-CH_2-CO-O-CH_2-CH_3), 2.34 (t, HO-CH_2-CH_2-CH_2-CH_2-**CH_2**-CO-O- CH_2-CH_3), 1.65 (m, HO-CH_2-CH_2-CH_2-**CH_2**-CH_2-CO-O-CH_2-CH_3), 1.57 (m, HO-CH_2-**CH_2**-CH_2-CH_2-CH_2-CO-O- CH_2-CH_3), 1.41 (m, HO-CH_2-CH_2-**CH_2**-CH_2-CH_2-CO-O- CH_2-CH_3), 1.26 (t, HO-CH_2-CH_2-CH_2-CH_2-CH_2-CO-O- CH_2-**CH_3**).

$^{13}C\{^1H\}$ NMR (126 MHz, CD_3OD): δC 173.86 (HO-CH_2-CH_2-CH_2-CH_2-CH_2-**CO**-O- CH_2-CH_3), 61.34 (HO-**CH_2**-CH_2-CH_2-CH_2-CH_2-CO-O- CH_2-CH_3), 59.99 (HO-CH_2-CH_2-CH_2-CH_2-CH_2-CO-O- **CH_2**-CH_3), 33.70 (HO-CH_2-CH_2-CH_2-CH_2-**CH_2**-CO-O- CH_2-CH_3), 31.92 (HO-CH_2-**CH_2**-CH_2-CH_2-CH_2-CO-O-CH_2-CH_3), 25.10,24.52 (HO-CH_2-CH_2-**CH_2-CH_2**-CH_2-CO-O- CH_2-CH_3), 13.22 (HO-CH_2-CH_2-CH_2-CH_2-CH_2-CO-O- CH_2-**CH_3**).

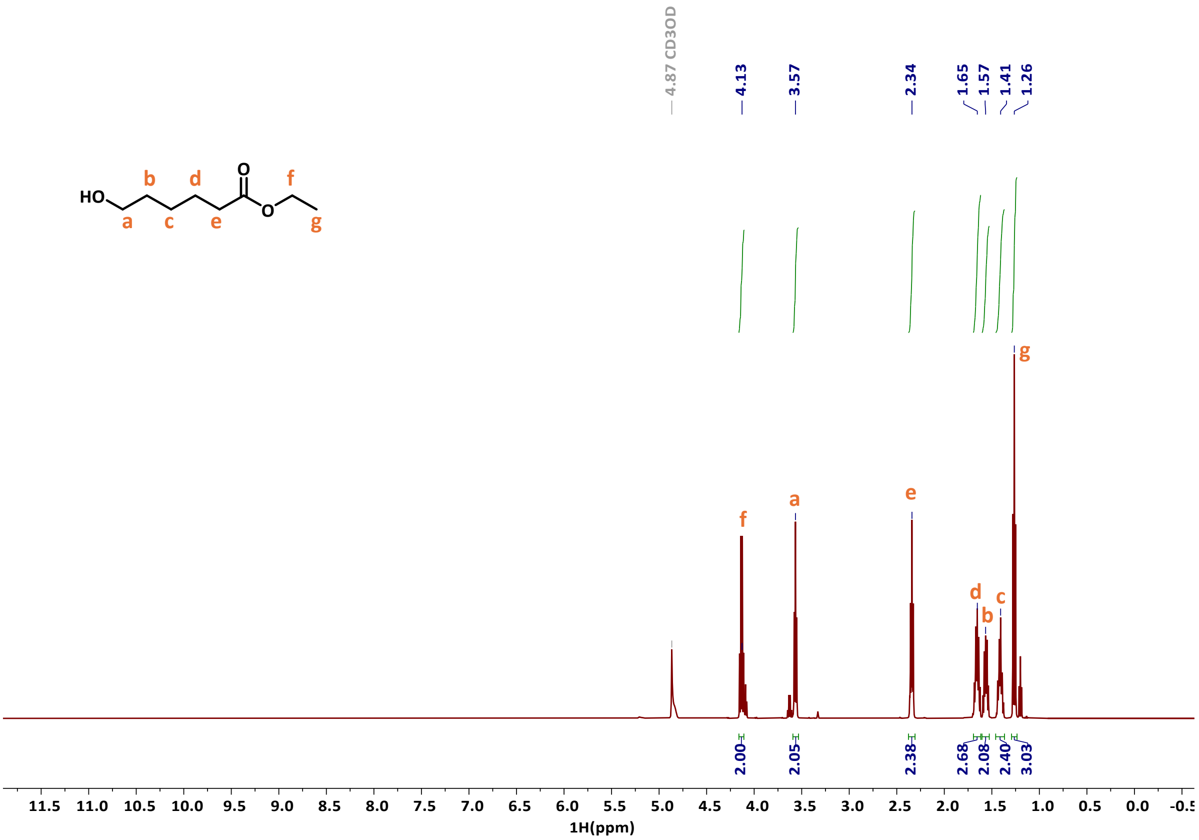

Figure S3. ^{1}H NMR (500 MHz, CD_3OD) spectrum of ethyl 6-hydroxyhexanoate.

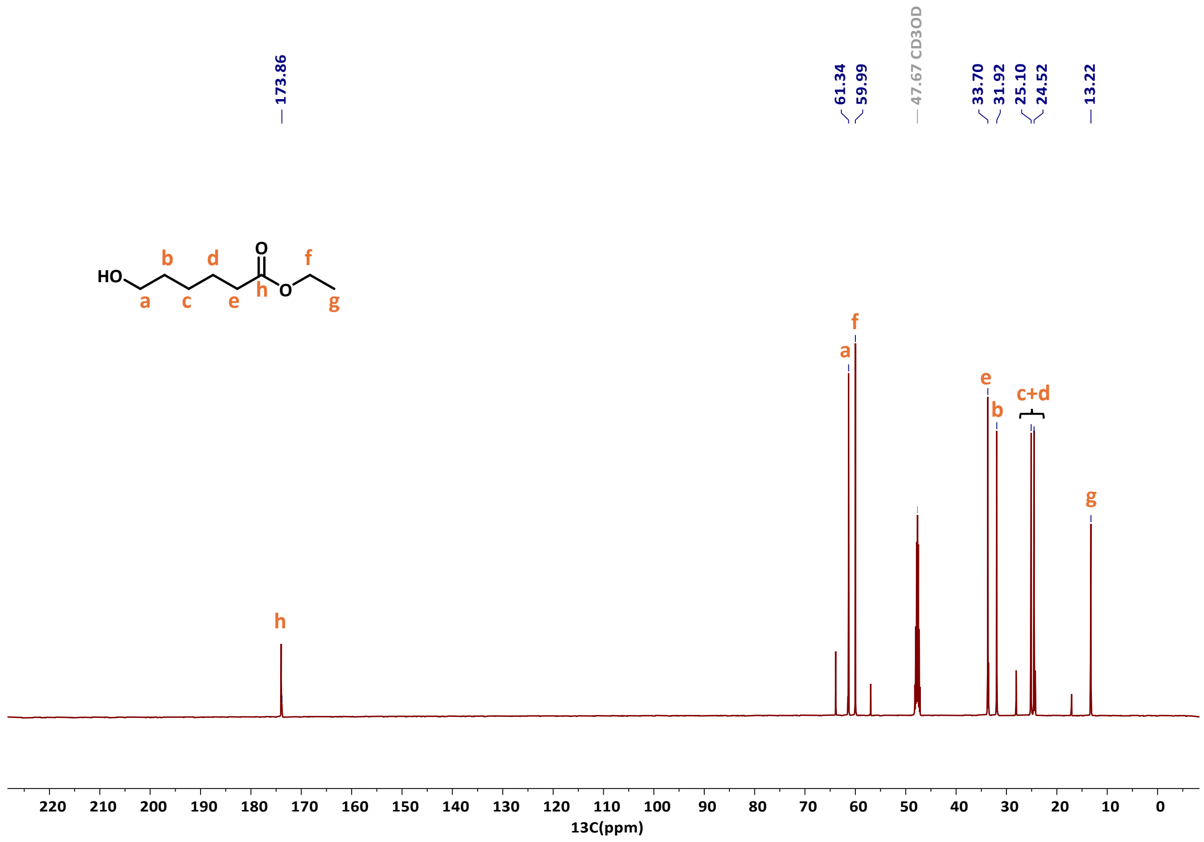

Figure S4. $^{13}C\{^1H\}$ NMR (126 MHz, CD_3OD) spectrum of ethyl 6-hydroxyhexanoate.

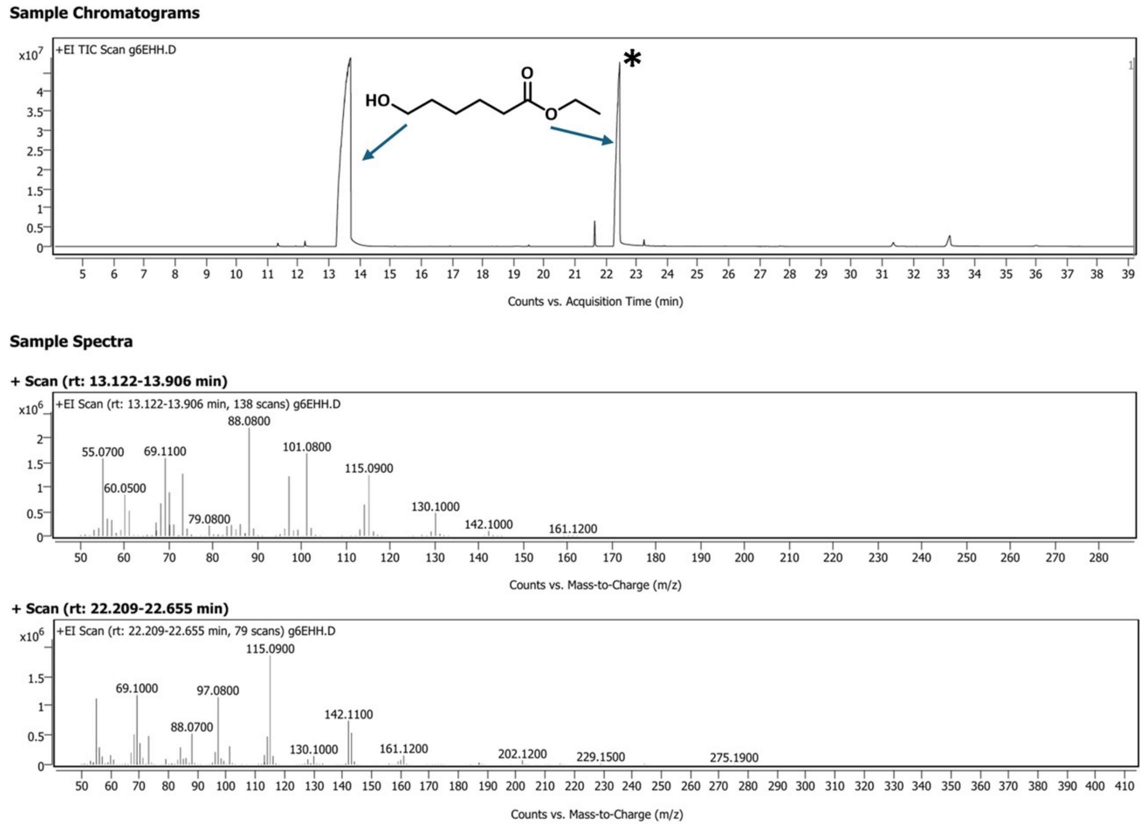

Figure S5. GC-MS data of Ethyl 6-hydroxyhexanoate.

* We speculate that the peak at 22.4 min could be due to the dimer formed during the GC-MS analysis due to high temperatures.

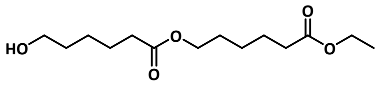

6. Characterisation of commercial ε-caprolactone

^{1}H NMR (500 MHz, CD_3OD): δH 4.31 (t, -O-$\mathbf{CH_2}$-CH_2-CH_2-CH_2-CH_2-CO-), 2.69 (t, -O-CH_2-CH_2-CH_2-CH_2-$\mathbf{CH_2}$-CO-), 1.86-1.81 (m, -O-CH_2-$\mathbf{CH_2}$-CH_2-$\mathbf{CH_2}$-CH_2-CO-), 1.74 (m, -O-CH_2-CH_2-$\mathbf{CH_2}$-CH_2-CH_2-CO-).

^{13}C{^{1}H} NMR (126 MHz, CD_3OD): δC 177.85(-O-CH_2-CH_2-CH_2-CH_2-CH_2-**CO**-), 69.14 (-O-$\mathbf{CH_2}$-CH_2-CH_2-CH_2-CH_2-CO-), 33.94 (-O-CH_2-CH_2-CH_2-CH_2-$\mathbf{CH_2}$-CO-), 29.13,28.43 (-O-CH_2-$\mathbf{CH_2}$-$\mathbf{CH_2}$-CH_2-CH_2-CO-), 22.70 (-O-CH_2-CH_2-CH_2-$\mathbf{CH_2}$-CH_2-CO-).

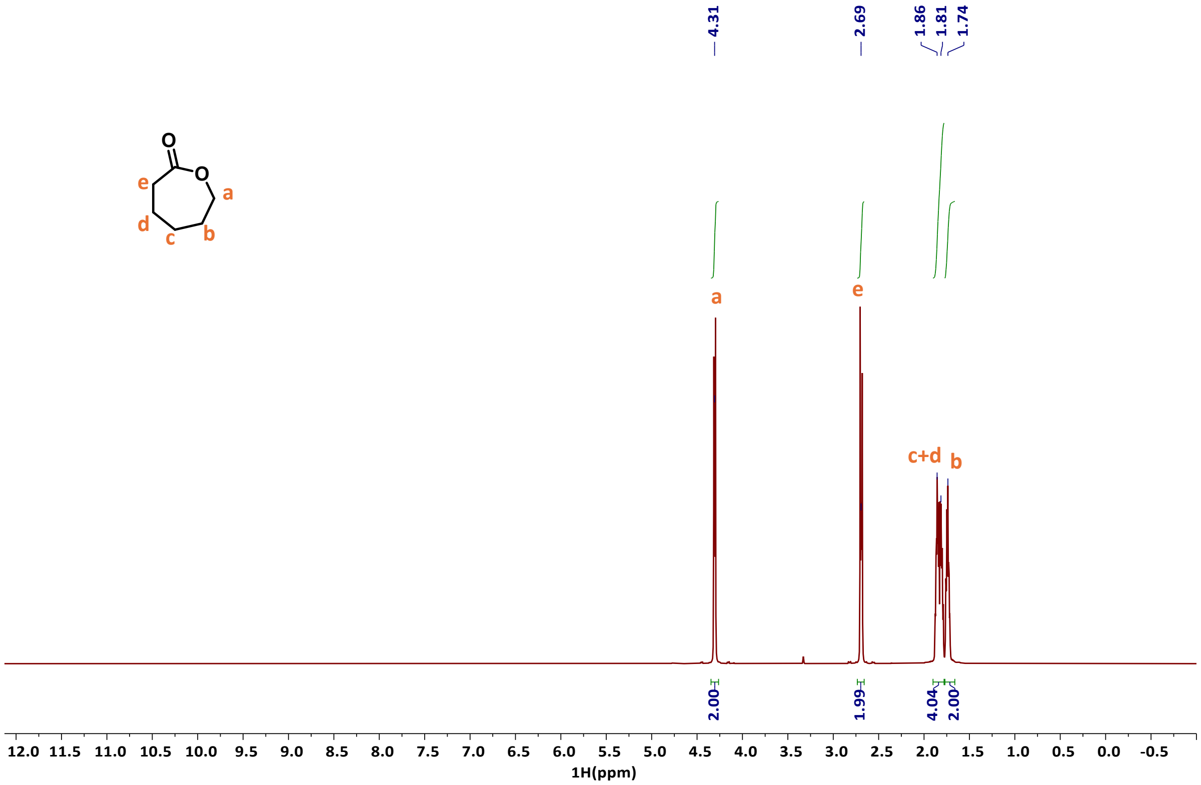

Figure S6. ^{1}H NMR (500 MHz, CD_3OD) spectrum of ε-caprolactone.

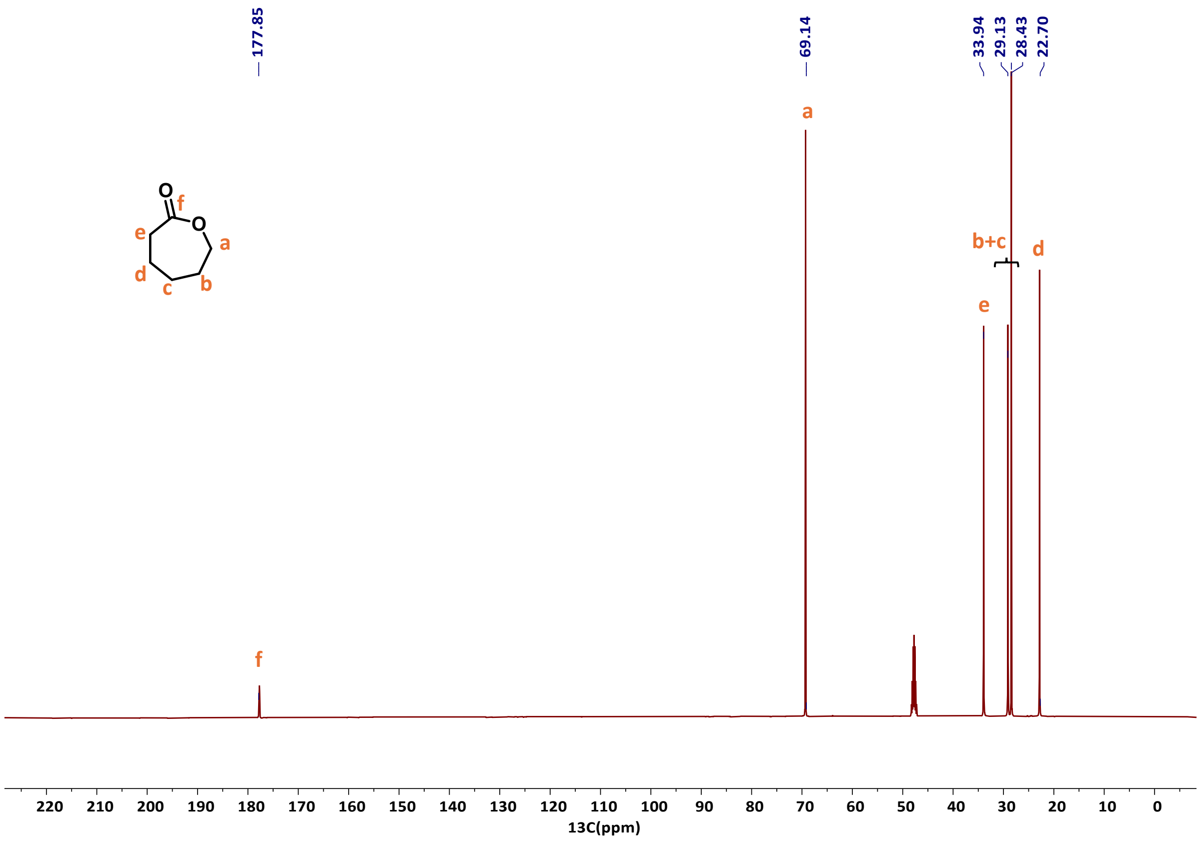

Figure S7. $^{13}C\{^1H\}$ NMR (126 MHz, CD_3OD) spectrum of ε-caprolactone.

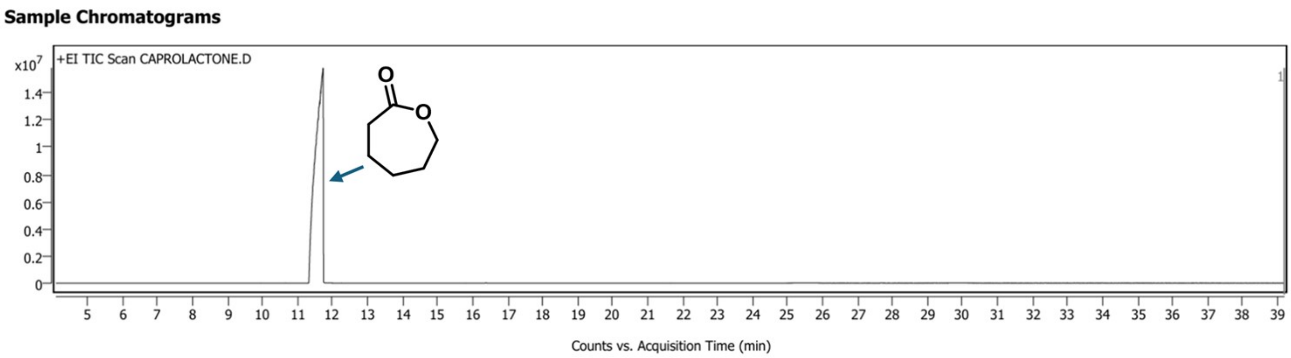

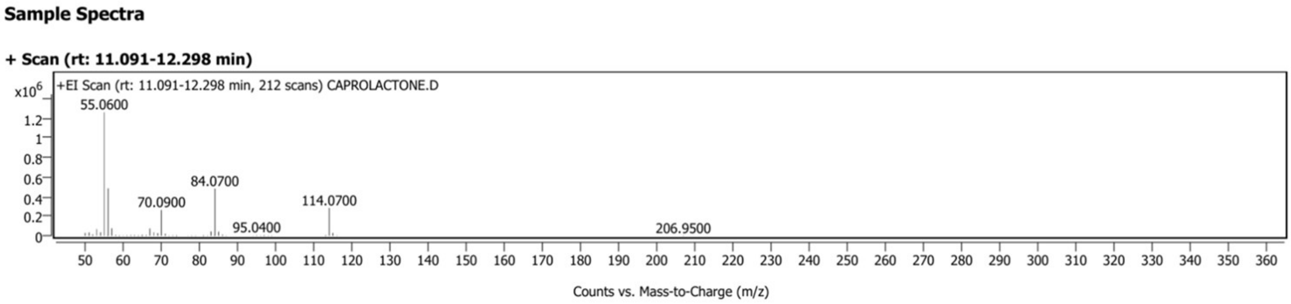

Figure S8. GC-MS data of ε-caprolactone.

7. Characterisation of isolated product-1,6-hexanediol

The following data were obtained for the product isolated from the reaction conducted as per the conditions described in Table S2, entry 8.

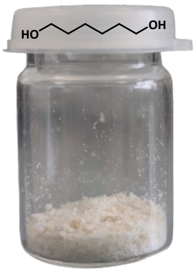

Yield of the 1,6-HD= 91%

^{1}H NMR (500 MHz, CD_3OD): δH 3.57 (t, HO-**CH_2**-CH_2-CH_2-CH_2-CH_2-**CH_2**-OH), 1.57 (t, HO-CH_2-**CH_2**-CH_2-CH_2-**CH_2**-CH_2-OH), 1.40 (t, HO-CH_2-CH_2-**CH_2**-**CH_2**-CH_2-CH_2-OH).

^{13}C{^{1}H} NMR (126 MHz, CD_3OD): δC 61.51 (HO-**CH_2**-CH_2-CH_2-CH_2-CH_2-**CH_2**-OH), 32.24 (HO-CH_2-**CH_2**-CH_2-CH_2-**CH_2**-CH_2-OH), 25.37 (HO-CH_2-CH_2-**CH_2**-**CH_2**-CH_2-CH_2-OH).

The NMR data matches well with the literature.[2]

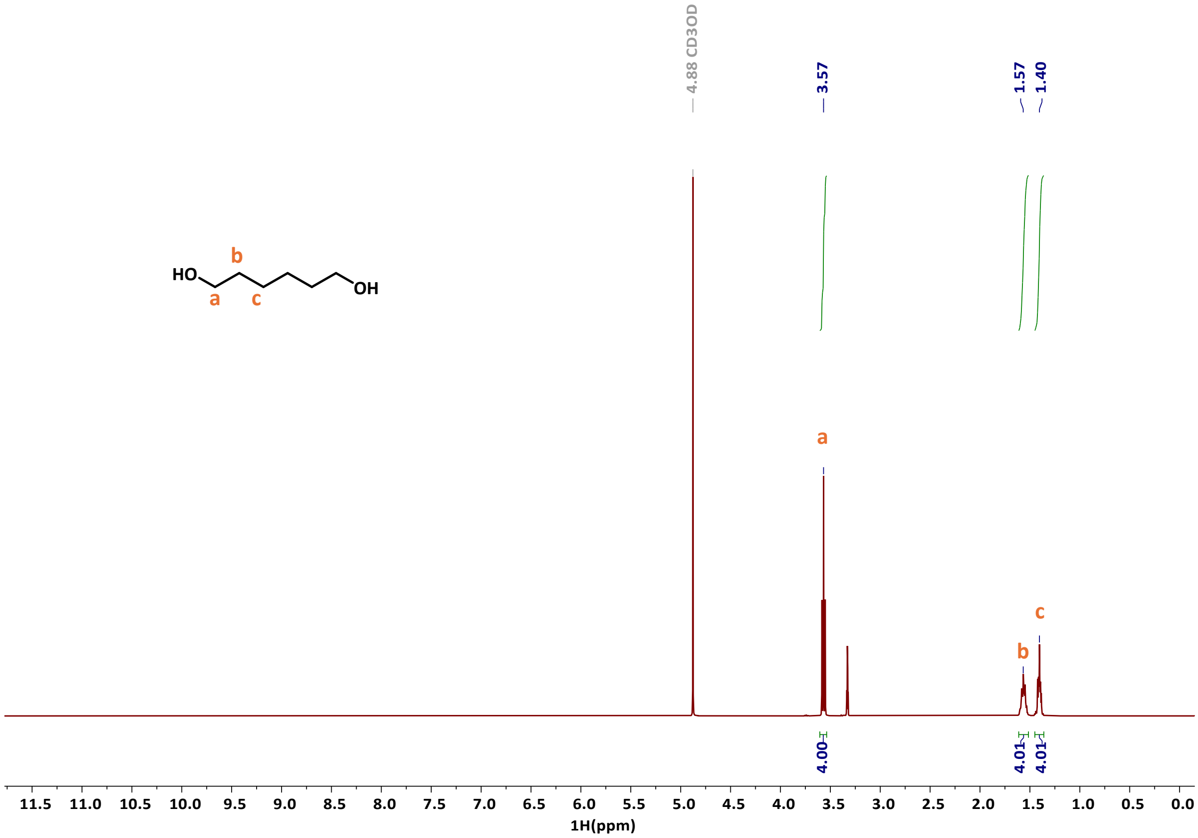

Figure S9. ^{1}H NMR (500 MHz, CD_3OD) spectrum of 1,6-hexanediol.

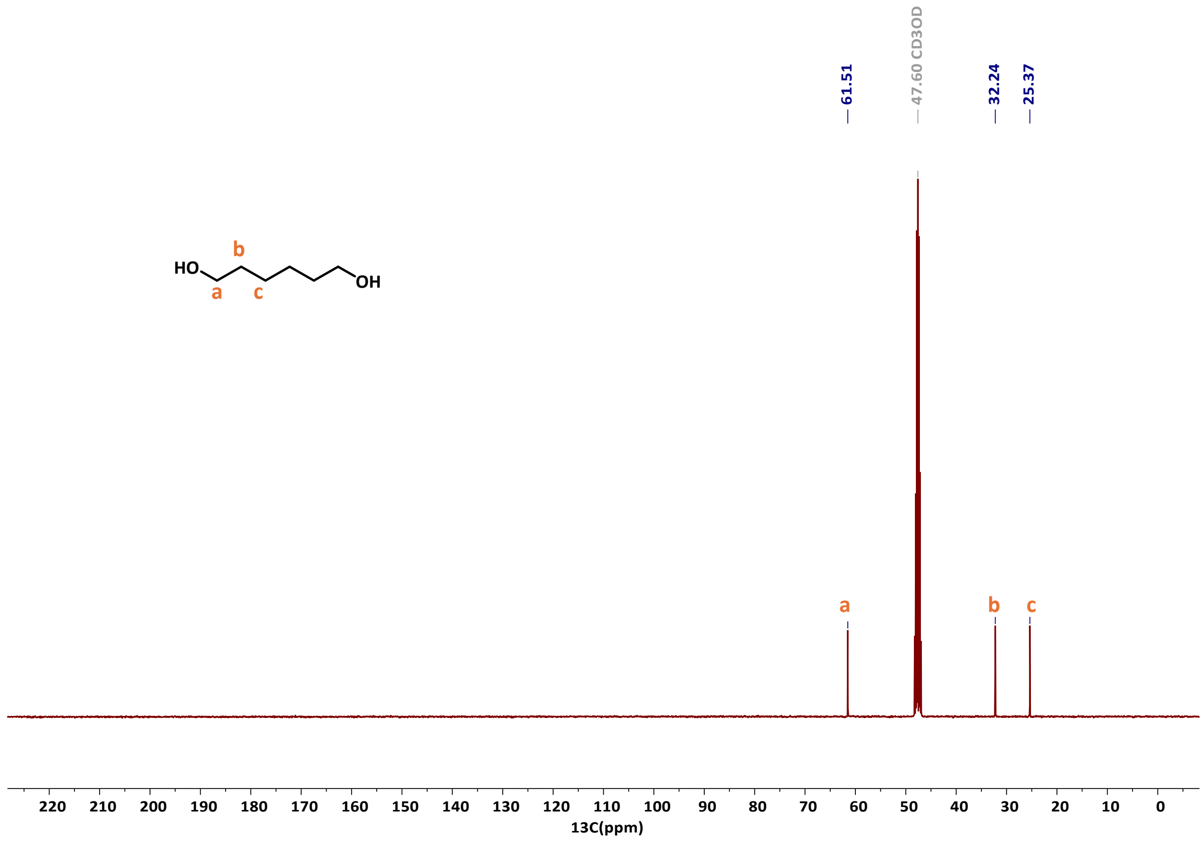

Figure S10. $^{13}C\{^{1}H\}$ NMR (126 MHz, CD_3OD) spectrum of 1,6-hexanediol.

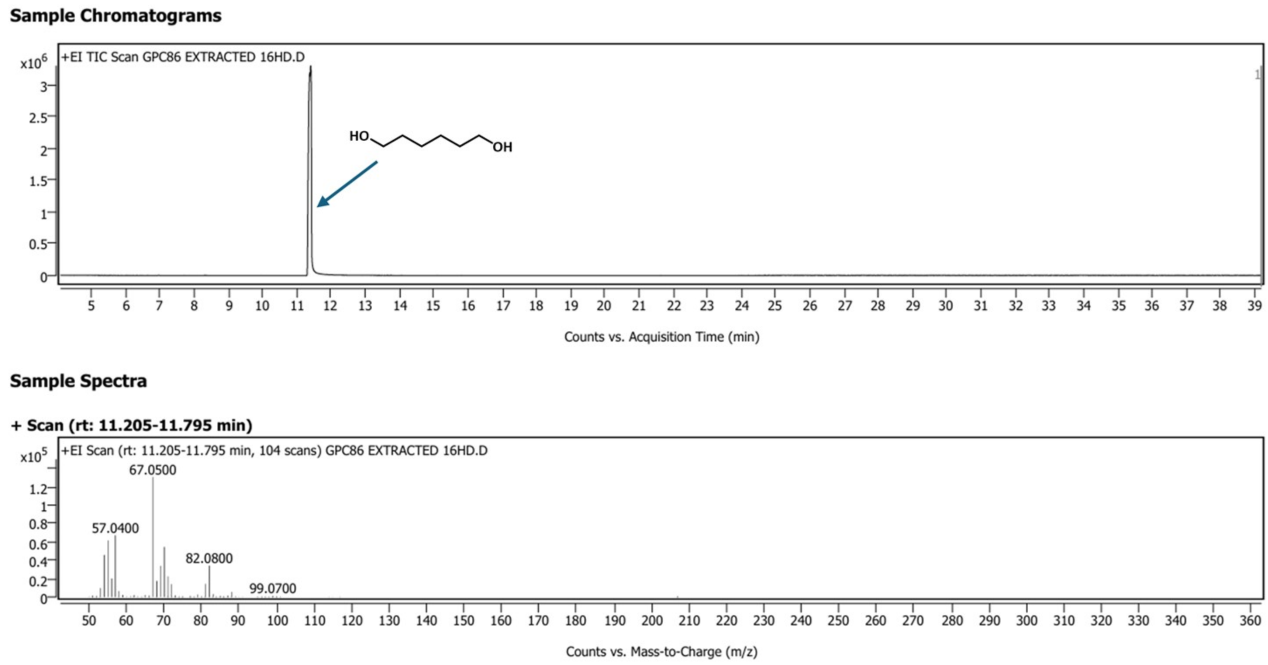

Figure S11. GC-MS data of 1,6-hexanediol.

8. Optimisation of Catalytic Conditions

Table S1. Optimisation of catalytic conditions for the hydrogenolysis of Polycaprolactone

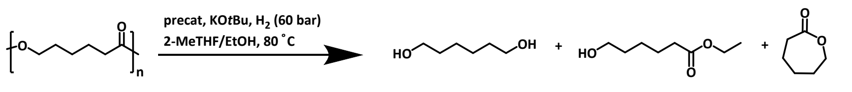

PCL, M_n 80,000 **1,6-HD** **E6HH** **ε-CPL**

Entry	Precatalyst	Precatalyst loading (mol%)	Base	Base loading (mol%)	2 Me THF (mL)	EtOH (mL)	Time,h	1,6-HD (%)	E6HH (%)	ε-CPL (%)	TON[b]
1	1	0.05	KO*t*Bu	10	1	0.1	20	54	10	0	1,080
2	2	0.05	KO*t*Bu	10	1	0.1	20	0	39	1	0
3	3	0.05	KO*t*Bu	10	1	0.1	20	0	44	1	0
4	4	0.05	KO*t*Bu	10	1	0.1	20	0	51	0	0
5	5	0.05	KO*t*Bu	10	1	0.1	20	42	17	0	840
6	6	0.05	KO*t*Bu	10	1	0.1	20	17	29	1	340
7	7	0.05	KO*t*Bu	10	1	0.1	20	87	0	0	1,740
8	8	0.05	KO*t*Bu	10	1	0.1	20	84	0	0	1,680
9	7	0.05	KO*t*Bu	10	2	0.25	20	93	0	0	1,860
10	8	0.05	KO*t*Bu	10	2	0.25	20	>99	0	0	2,000
11	8	0.02	KO*t*Bu	10	2	0.25	20	36	40	2	1,800
12	8	0.01	KO*t*Bu	10	2	0.25	20	34	40	2	3,400
13	-	-	KO*t*Bu	10	2	0.25	20	0	48	10	0
14[c]	8	0.05	KO*t*Bu	10	2	0.25	20	88	0	0	1,760
15	8	0.05	KO*t*Bu	-	2	0.25	20	0	0	0	0
16	8	0.05	KO*t*Bu	2	2	0.25	20	12	52	1	240
17	8	0.05	KO*t*Bu	5	2	0.25	20	>99	0	0	2,000
18	8	0.05	KOH	5	2	0.25	20	93	0	0	1,860
19	8	0.05	NaO*t*Bu	5	2	0.25	20	96	0	0	1,920
20[d]	8	0.05	KO*t*Bu	5	2	0.25	20	25	39	1	500
21[e]	8	0.05	KO*t*Bu	5	2	0.25	20	87	0	0	1,740
22[f]	8	0.05	KO*t*Bu	5	2	0.25	20	0	50	8	0
23[g]	8	0.05	KO*t*Bu	5	2	0.25	20	84	0	0	1,680
24	8	0.05	KO*t*Bu	5	2	-	20	13[h]	0	0	260
25	8	0.05	KO*t*Bu	5	2	0.1	20	87	0	0	1,740
26	8	0.01	KO*t*Bu	5	2	0.25	72	92	0	0	9,200
27	8	0.005	KO*t*Bu	5	2	0.25	72	79	6	0	15,800

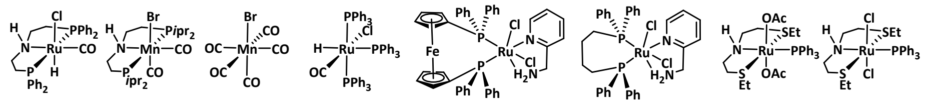

1 **2** **3** **4** **5** **6** **7** **8**

[a]Polycaprolactone, Mn 80,000 (1 mmol of monomeric unit), 2-Me THF, EtOH, KO*t*Bu (1M solution in THF), precatalyst (2 mM solution in 2-Me THF), 80 °C, 60 bar H_2. Yields were estimated by quantitive GC-MS analysis using mesitylene as an internal standard. [b]TON estimated based on the number of ester groups hydrogenated. [c] Reaction done at 60 °C, [d] THF used as solvent. [e] Toluene used as solvent. [f] No external hydrogen pressure. [g]30 bar H_2 pressure.[h] ^{1}HNMR yield.

Table S1; Entry 1:

^{1}H NMR (500 MHz, CD_3OD): δH 4.07 (t, -O-**CH₂**-CH₂-CH₂-CH₂-CH₂-CO-), 2.32 (t, -O-CH₂-CH₂-CH₂-CH₂-**CH₂**-CO-), 1.66-1.63 (m, -O-CH₂-**CH₂**-CH₂-**CH₂**-CH₂-CO-), 1.41 (m, -O-CH₂-CH₂-**CH₂**-CH₂-CH₂-CO-), 4.13 (q, HO-CH₂-CH₂-CH₂-CH₂-CH₂-CO-O- **CH₂**-CH₃), 3.57 (t, HO-**CH₂**-CH₂-CH₂-CH₂-CH₂-CO-O- CH₂-CH₃), 2.34 (t, HO-CH₂-CH₂-CH₂-CH₂-**CH₂**-CO-O- CH₂-CH₃), 1.65 (m, HO-CH₂-CH₂-CH₂-**CH₂**-CH₂-CO-O-CH₂-CH₃), 1.57 (m, HO-CH₂-**CH₂**-CH₂-CH₂-CH₂-CO-O- CH₂-CH₃), 1.41 (m, HO-CH₂-CH₂-**CH₂**-CH₂-CH₂-CO-O-CH₂-CH₃), 1.26 (t, HO-CH₂-CH₂-CH₂-CH₂-CH₂-CO-O- CH₂-**CH₃**), 3.57 (t, HO-**CH₂**-CH₂-CH₂-CH₂-CH₂-**CH₂**-OH), 1.57 (t, HO-CH₂-**CH₂**-CH₂-CH₂-**CH₂**-CH₂-OH), 1.40 (t, HO-CH₂-CH₂-**CH₂**-**CH₂**-CH₂-CH₂-OH).

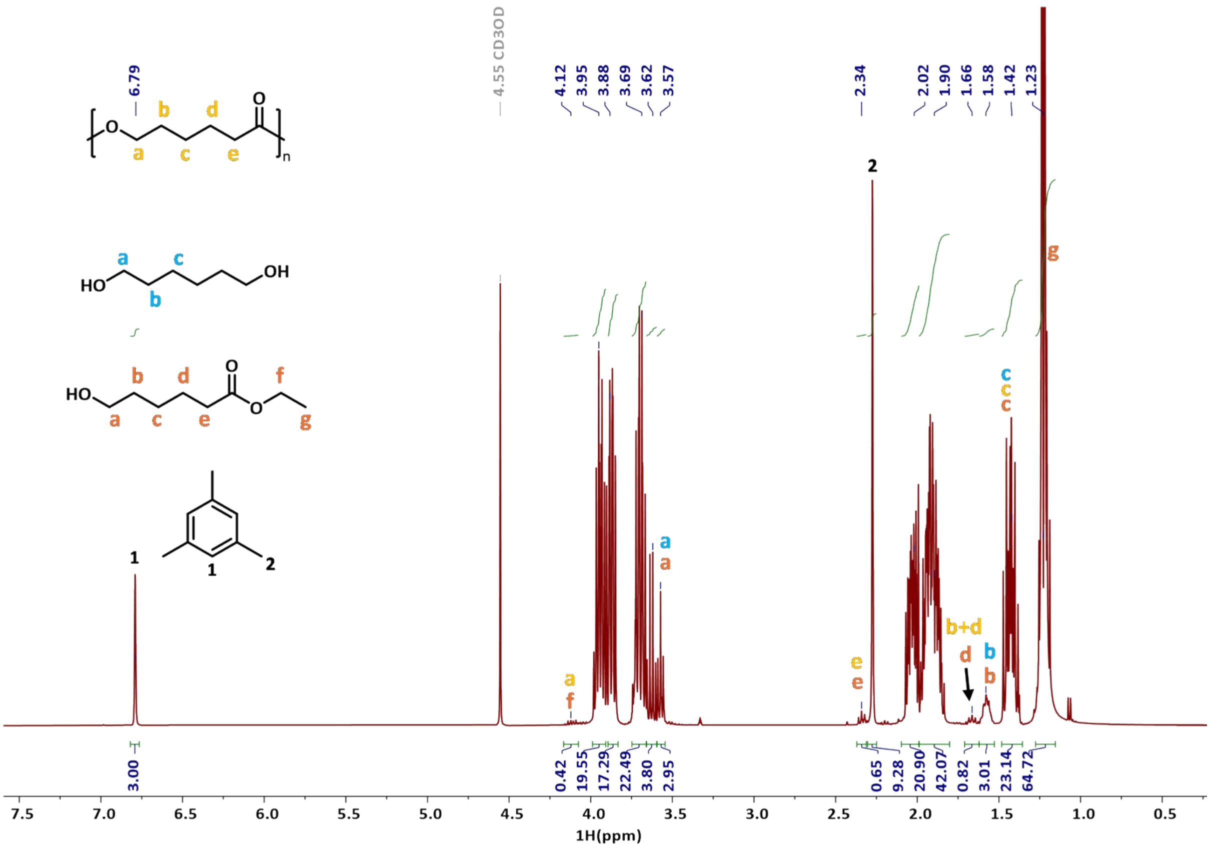

Figure S12. ^{1}H NMR (400 MHz, CD_3OD) spectrum of the reaction mixture resulting from the reaction corresponding to Table S1; Entry 1. Mesitylene is used as an internal standard.

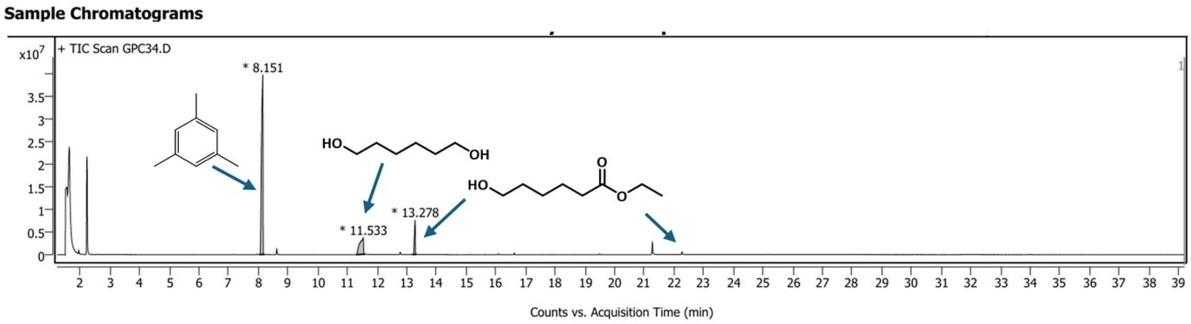

Chromatogram Peaks

Peak	Start	RT	End	Height	Area	Area %	SNR
1	8.031	8.151	8.208	39659049	136997433	100.00	
2	11.292	11.533	11.607	3648789	32560007	23.77	
3	13.186	13.278	13.335	7610996	15399147	11.24	

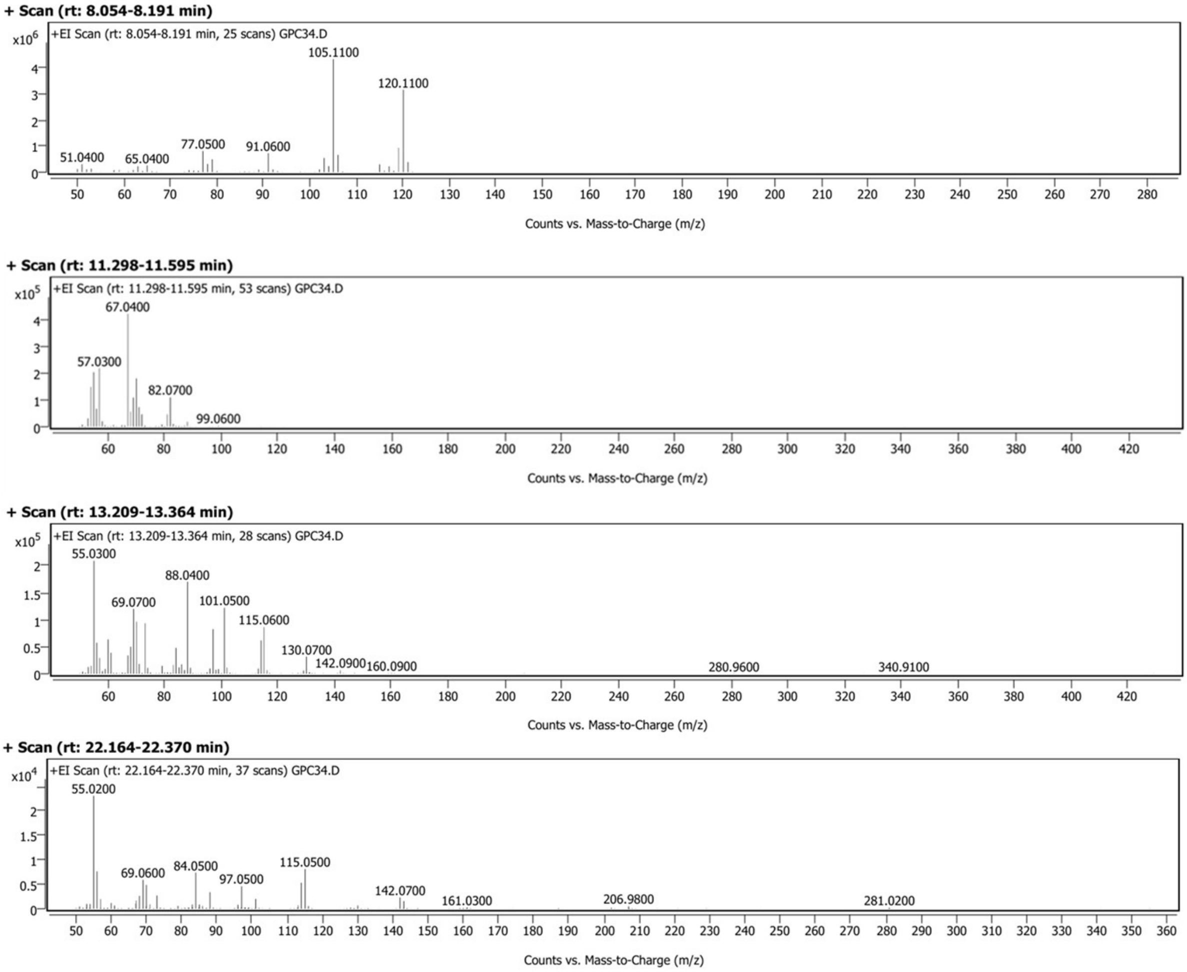

Figure S13. GC-MS data of the reaction mixture resulting from the reaction corresponding to Table S1; Entry 1.

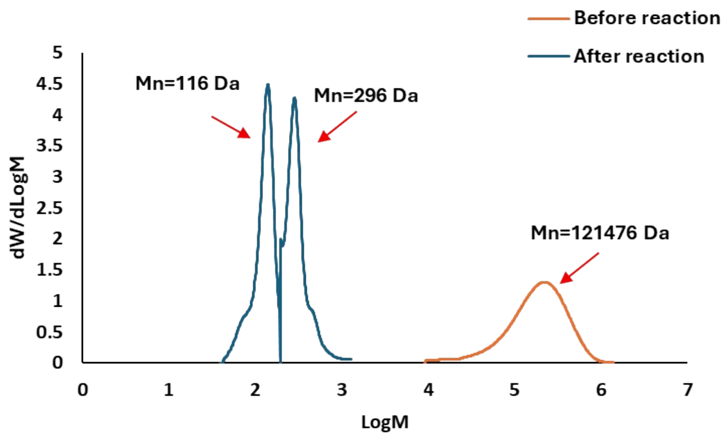

Figure S14. GPC data of the reaction mixture resulting from the reaction corresponding to Table S1; Entry 1.

Table S1; Entry 2:

Sample Chromatograms

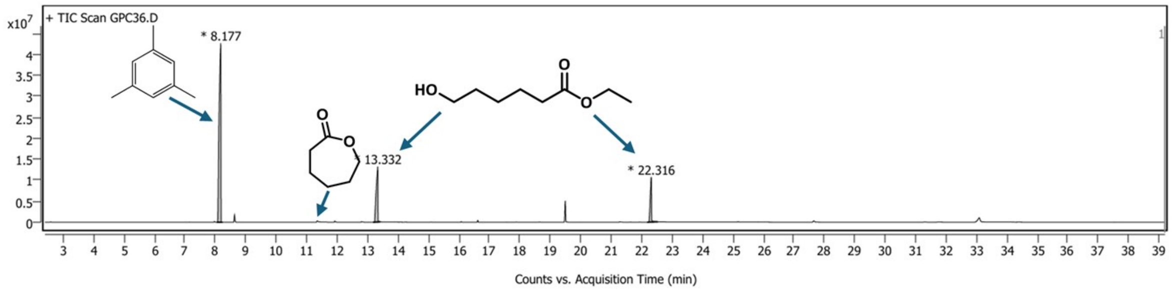

Chromatogram Peaks

Peak	Start	RT	End	Height	Area	Area %	SNR
1	8.051	8.177	8.228	42682037	175739061	100.00	
2	13.184	13.332	13.418	13211548	52025308	29.60	
3	22.184	22.316	22.533	10744000	31023317	17.65	

Sample Spectra

+ Scan (rt: 11.313-11.404 min)

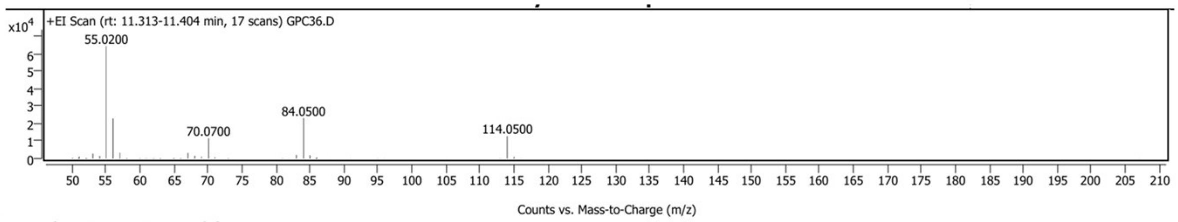

+ Scan (rt: 13.189-13.424 min)

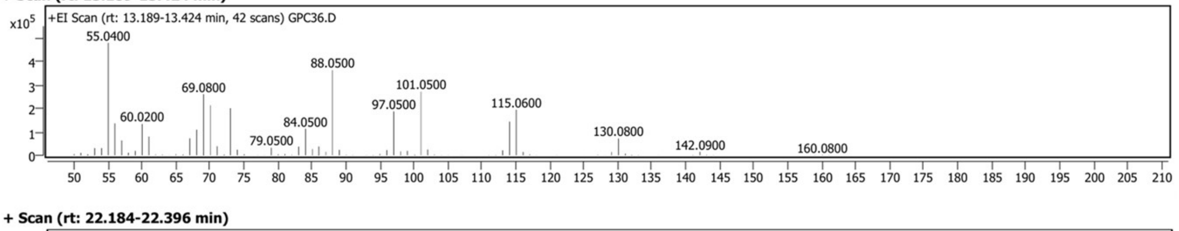

+ Scan (rt: 22.184-22.396 min)

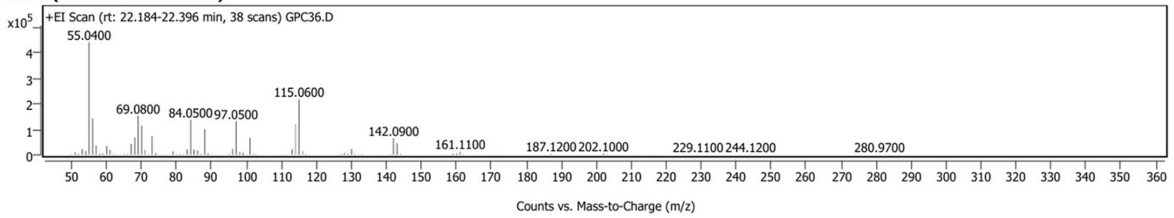

Figure S15. GC-MS data of the reaction mixture resulting from the reaction corresponding to Table S1; Entry 2.

Table S1; Entry 3:

Sample Chromatograms

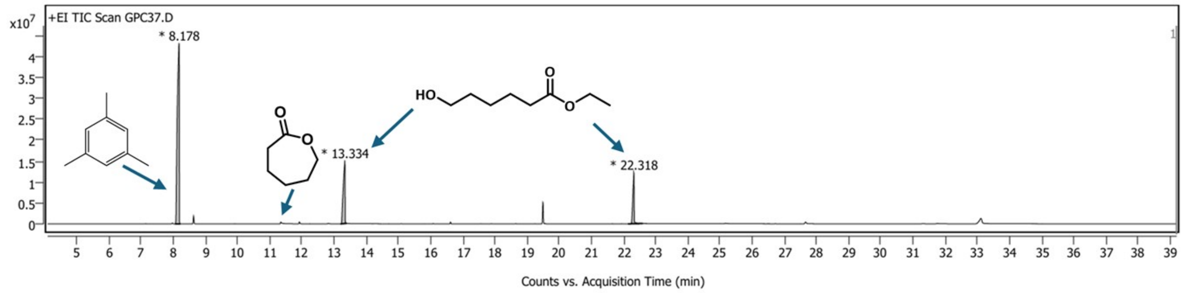

Chromatogram Peaks

Peak	Start	RT	End	Height	Area	Area %	SNR
1	8.052	8.178	8.218	43053845	183916064	100.00	
2	13.197	13.334	13.391	15035196	58431082	31.77	
3	22.140	22.318	22.598	12178582	34862724	18.96	

Sample Spectra

+ Scan (rt: 11.308-11.423 min)

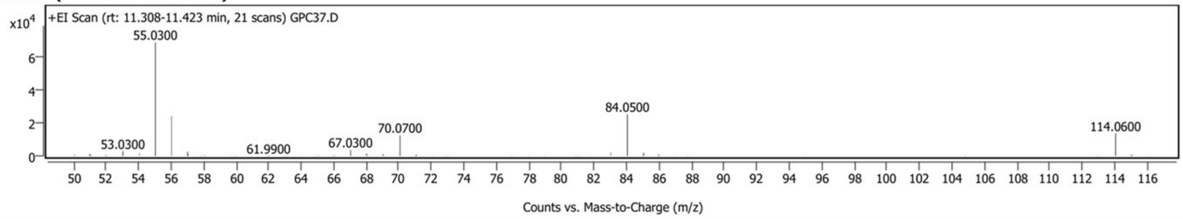

+ Scan (rt: 13.202-13.431 min)

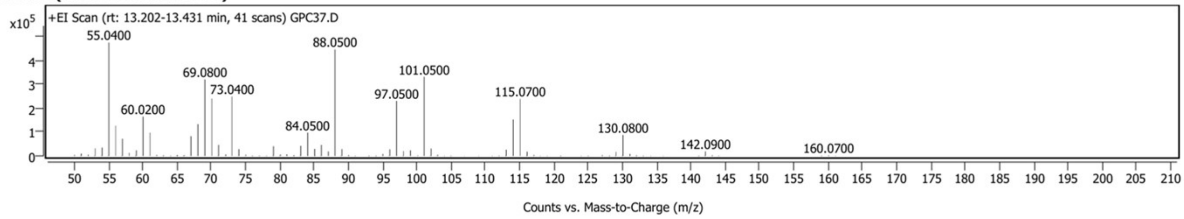

+ Scan (rt: 22.232-22.363 min)

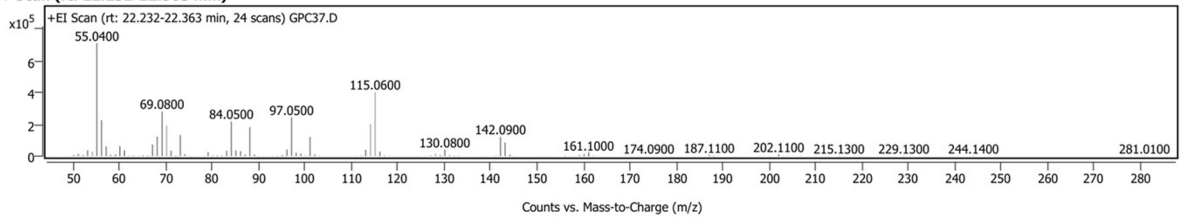

Figure S16. GC-MS data of the reaction mixture resulting from the reaction corresponding to Table S1; Entry 3.

Table S1; Entry 4

Sample Chromatograms

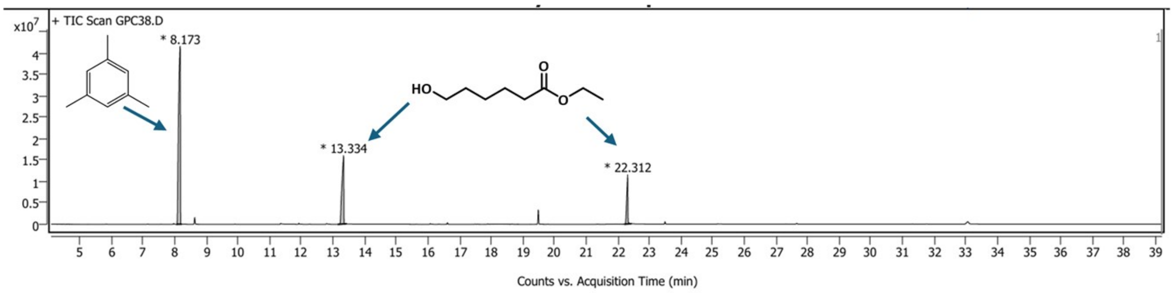

Chromatogram Peaks

Peak	Start	RT	End	Height	Area	Area %	SNR
1	8.053	8.173	8.218	41440991	163581520	100.00	
2	13.151	13.334	13.431	15979968	64485269	39.42	
3	22.197	22.312	22.432	11502873	29199212	17.85	

Sample Spectra

+ Scan (rt: 13.179-13.483 min)

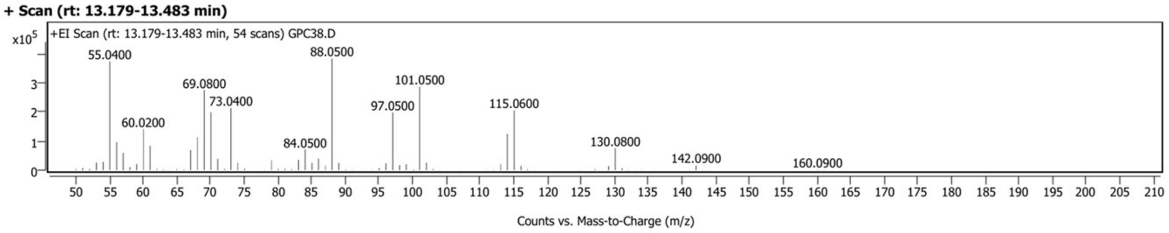

+ Scan (rt: 22.123-22.484 min)

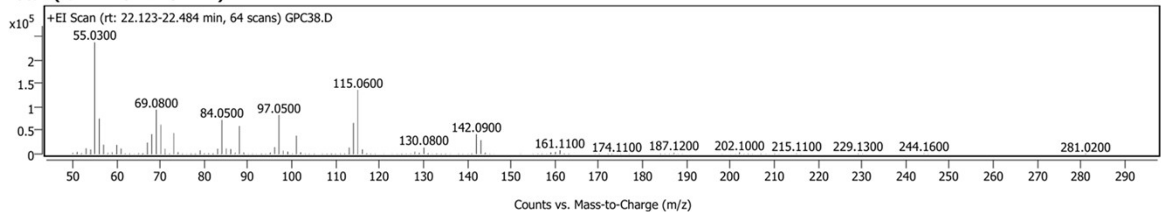

Figure S17. GC-MS data of the reaction mixture resulting from the reaction corresponding to Table S1; Entry 4.

<u>Table S1; Entry 5</u>

Sample Chromatograms

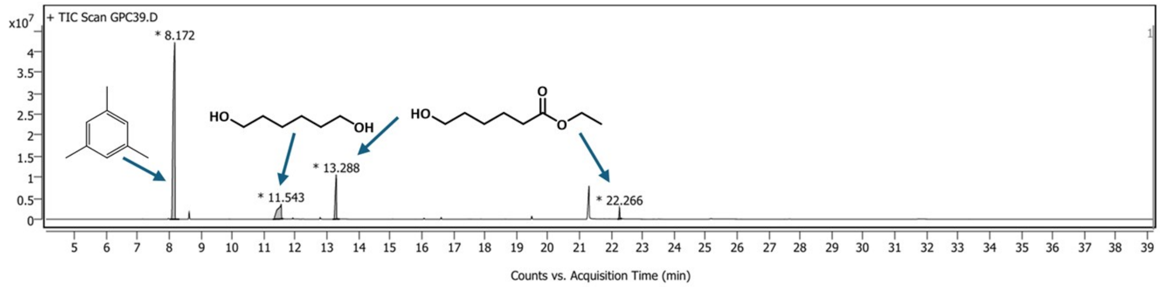

Chromatogram Peaks

Peak	Start	RT	End	Height	Area	Area %	SNR
1	8.018	8.172	8.315	42166562	171687092	100.00	
2	11.285	11.543	11.611	3497659	30401915	17.71	
3	13.179	13.288	13.397	10520258	27377143	15.95	
4	22.203	22.266	22.346	2747357	4509926	2.63	

+ Scan (rt: 11.308-11.577 min)

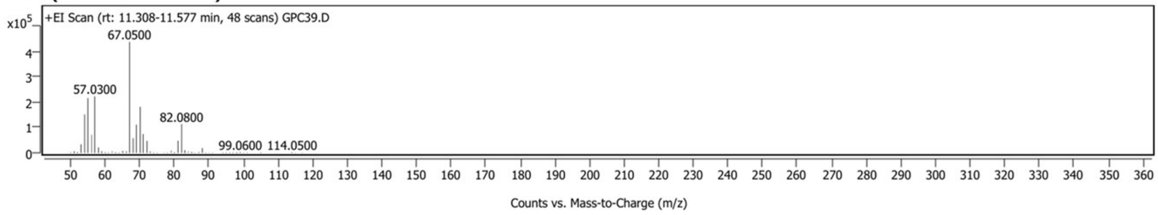

+ Scan (rt: 13.122-13.431 min)

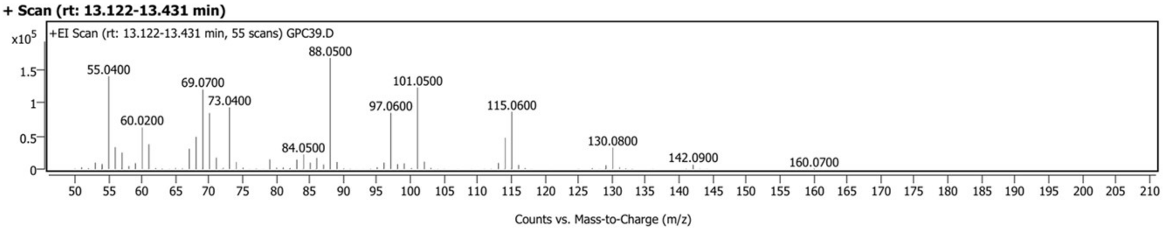

+ Scan (rt: 22.180-22.403 min)

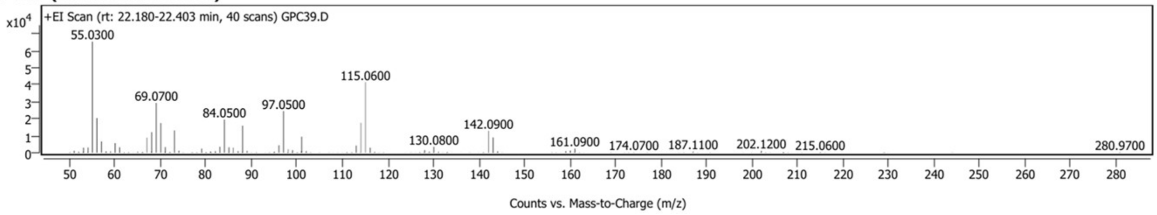

Figure S18. GC-MS data of the reaction mixture resulting from the reaction corresponding to Table S1; Entry 5.

<u>Table S1; Entry 6:</u>

Sample Chromatograms

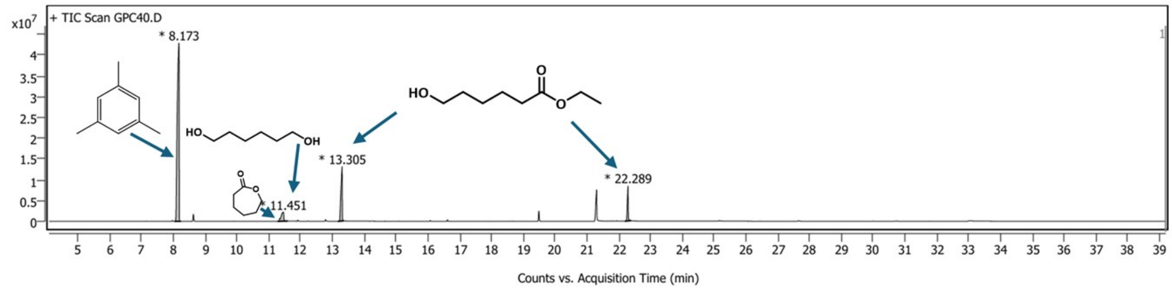

Chromatogram Peaks

Peak	Start	RT	End	Height	Area	Area %	SNR
1	8.035	8.173	8.236	42893495	175547490	100.00	
2	11.274	11.451	11.595	2170632	13041993	7.43	
3	13.168	13.305	13.357	13112016	38735477	22.07	
4	22.192	22.289	22.386	8423945	16902152	9.63	

Sample Spectra

+ Scan (rt: 11.360-11.537 min)

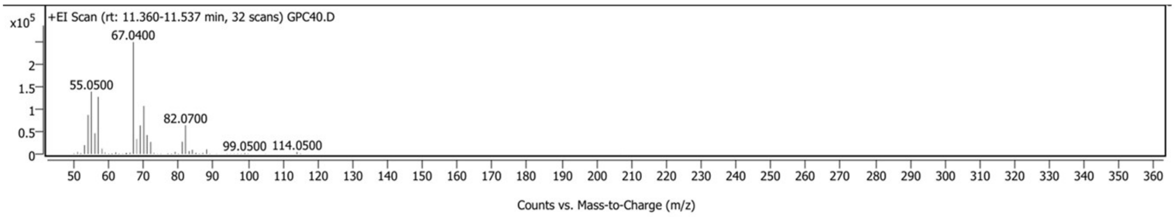

+ Scan (rt: 11.308-11.354 min)

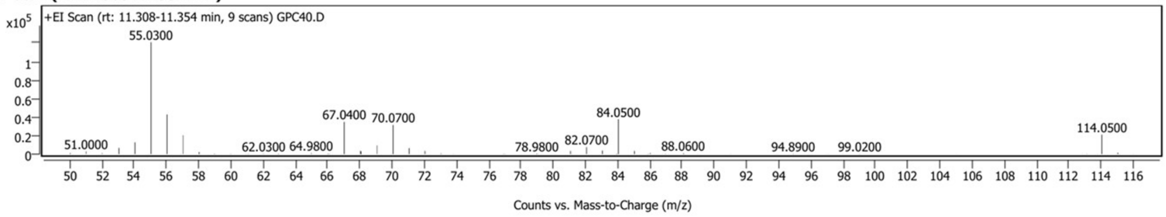

+ Scan (rt: 13.180-13.374 min)

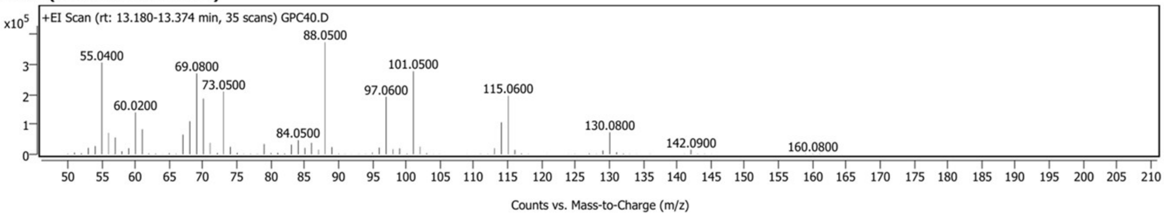

+ Scan (rt: 22.209-22.346 min)

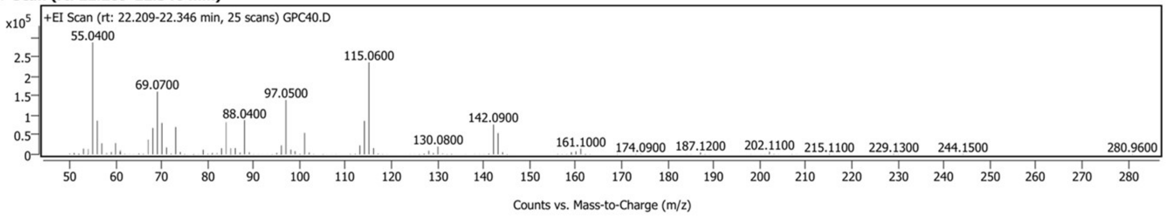

Figure S19. GC-MS data of the reaction mixture resulting from the reaction corresponding to Table S1; Entry 6.

Table S1; Entry 7:

Sample Chromatograms

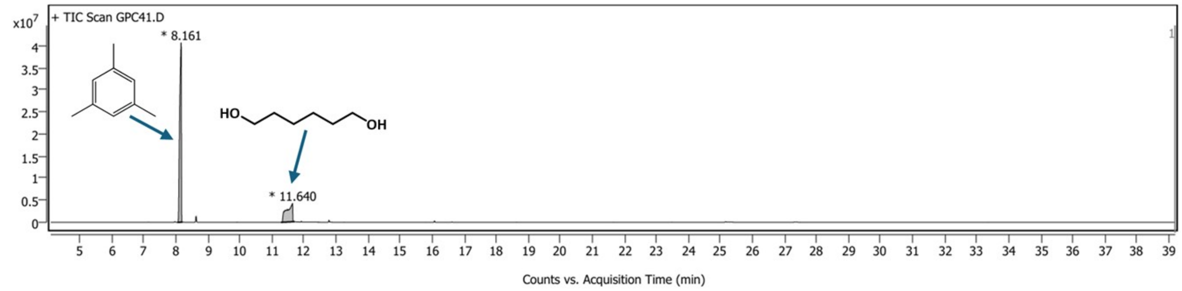

Chromatogram Peaks

Peak	Start	RT	End	Height	Area	Area %	SNR
1	8.047	8.161	8.201	40575809	146459168	100.00	
2	11.286	11.640	11.703	4115618	54559931	37.25	

Sample Spectra

+ Scan (rt: 11.303-11.680 min)

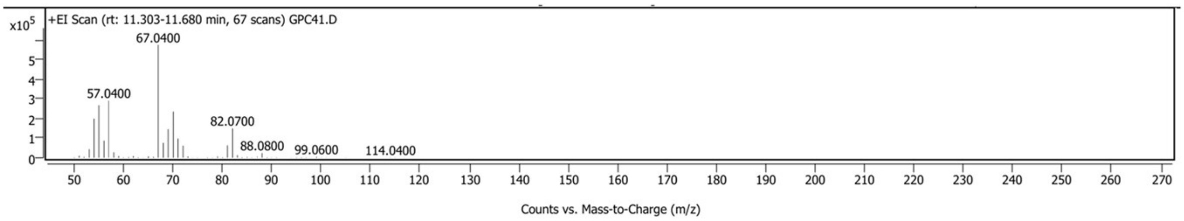

Figure S20. GC-MS data of the reaction mixture resulting from the reaction corresponding to Table S1; Entry 7.

Table S1; Entry 8:

Sample Chromatograms

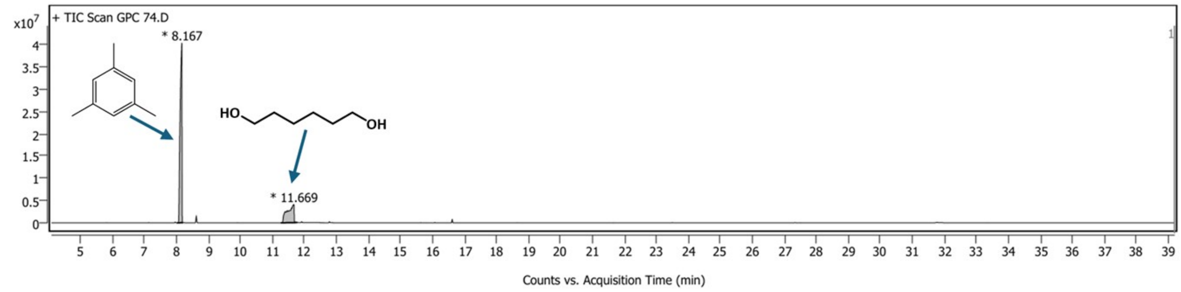

Chromatogram Peaks

Peak	Start	RT	End	Height	Area	Area %	SNR
1	8.018	8.167	8.201	40286202	160745948	100.00	
2	11.263	11.669	11.778	3993432	58942166	36.67	

Sample Spectra

+ Scan (rt: 11.309-11.692 min)

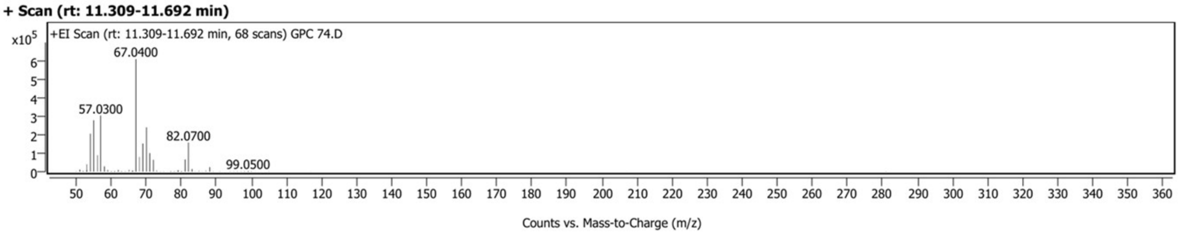

Figure S21. GC-MS data of the reaction mixture resulting from the reaction corresponding to Table S1; Entry 8.

Table S1; Entry 9:

Sample Chromatograms

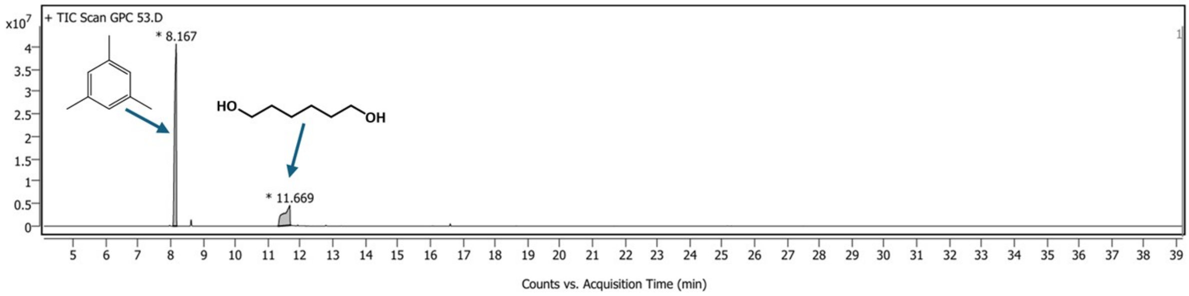

Chromatogram Peaks

Peak	Start	RT	End	Height	Area	Area %	SNR
1	8.058	8.167	8.201	40612083	155221248	100.00	
2	11.297	11.669	11.709	4427296	61503108	39.62	

Sample Spectra

+ Scan (rt: 11.291-11.824 min)

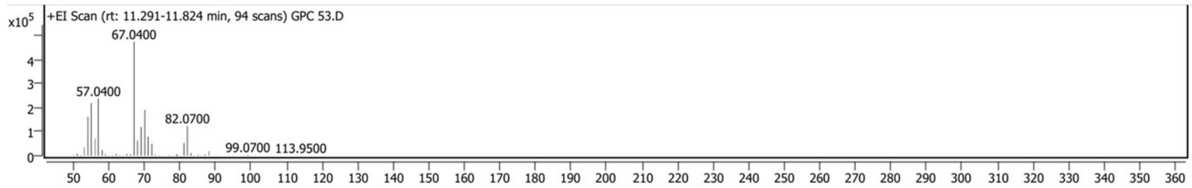

Figure S22. GC-MS data of the reaction mixture resulting from the reaction corresponding to Table S1; Entry 9.

Table S1; Entry 10:

Sample Chromatograms

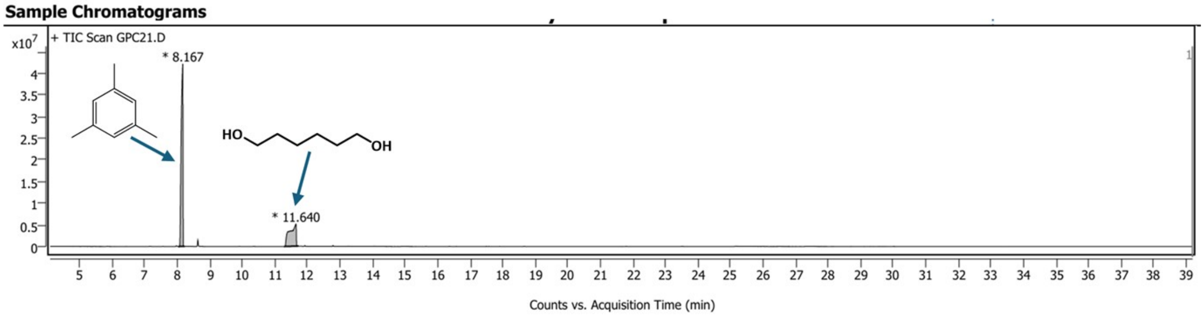

Chromatogram Peaks

Peak	Start	RT	End	Height	Area	Area %	SNR
1	8.035	8.167	8.230	42142380	156081622	100.00	
2	11.286	11.640	11.720	5095010	71698786	45.94	

Sample Spectra

+ Scan (rt: 11.291-11.726 min)

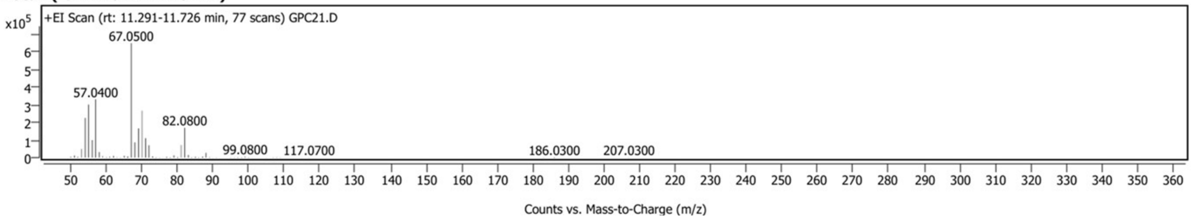

Figure S23. GC-MS data of the reaction mixture resulting from the reaction corresponding to Table S1; Entry 10.

Table S1; Entry 11:

Sample Chromatograms

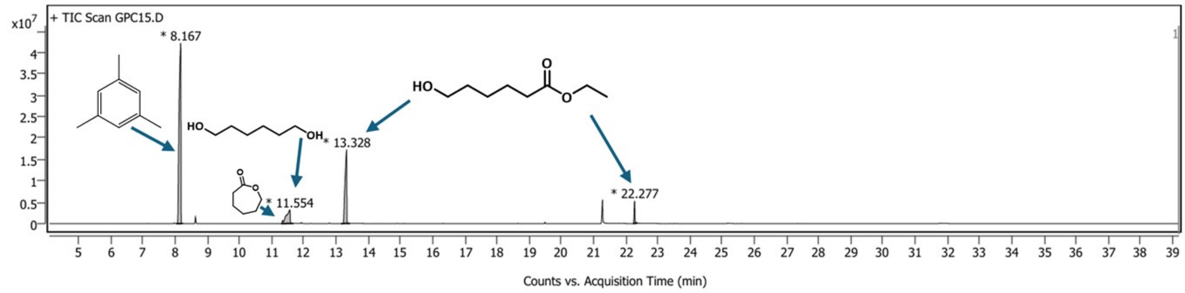

Chromatogram Peaks

Peak	Start	RT	End	Height	Area	Area %	SNR
1	8.035	8.167	8.236	42164539	162955359	100.00	
2	11.297	11.554	11.675	3168388	26543773	16.29	
3	13.157	13.328	13.431	17268723	65168840	39.99	
4	22.232	22.277	22.363	5238688	8502745	5.22	

Sample Spectra

+ Scan (rt: 11.291-11.366 min)

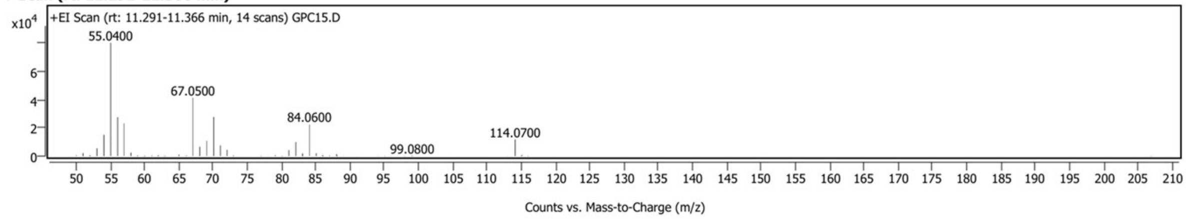

+ Scan (rt: 11.383-11.600 min)

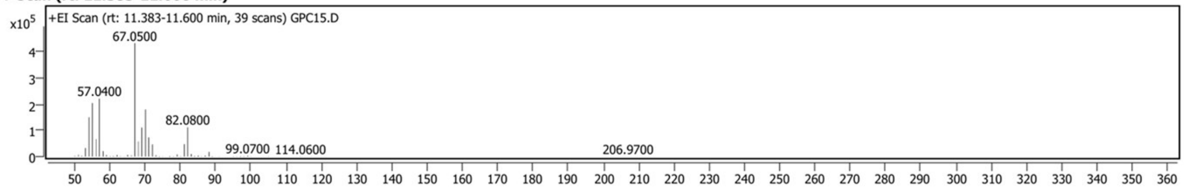

+ Scan (rt: 13.197-13.391 min)

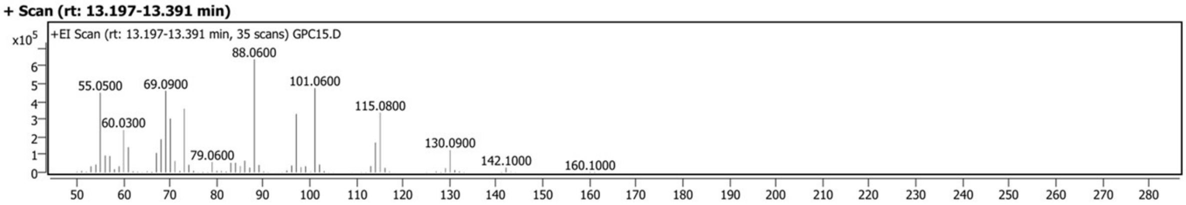

+ Scan (rt: 22.192-22.363 min)

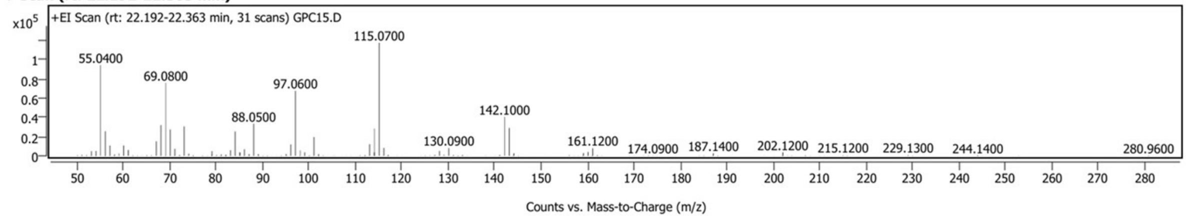

Figure S24. GC-MS data of the reaction mixture resulting from the reaction corresponding to Table S1; Entry 11.

<ins>Table S1; Entry 12:</ins>

Sample Chromatograms

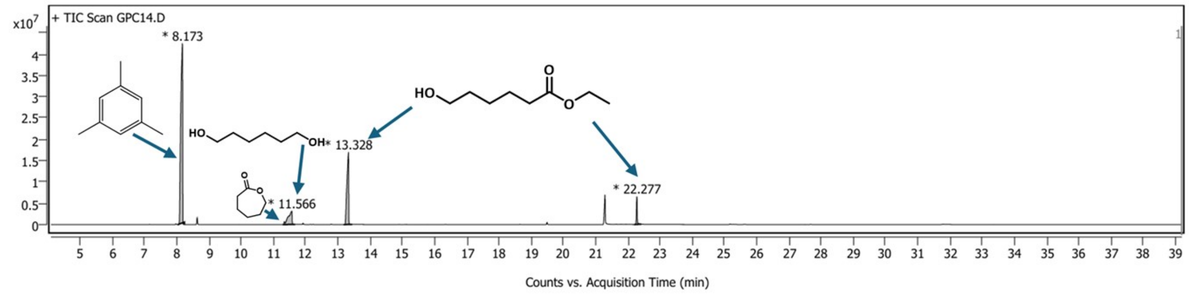

Chromatogram Peaks

Peak	Start	RT	End	Height	Area	Area %	SNR
1	8.030	8.173	8.236	42079215	165884101	100.00	
2	11.291	11.566	11.669	3090349	25998567	15.67	
3	13.179	13.328	13.437	16930708	64158822	38.68	
4	22.197	22.277	22.415	6518556	11235150	6.77	

Sample Spectra

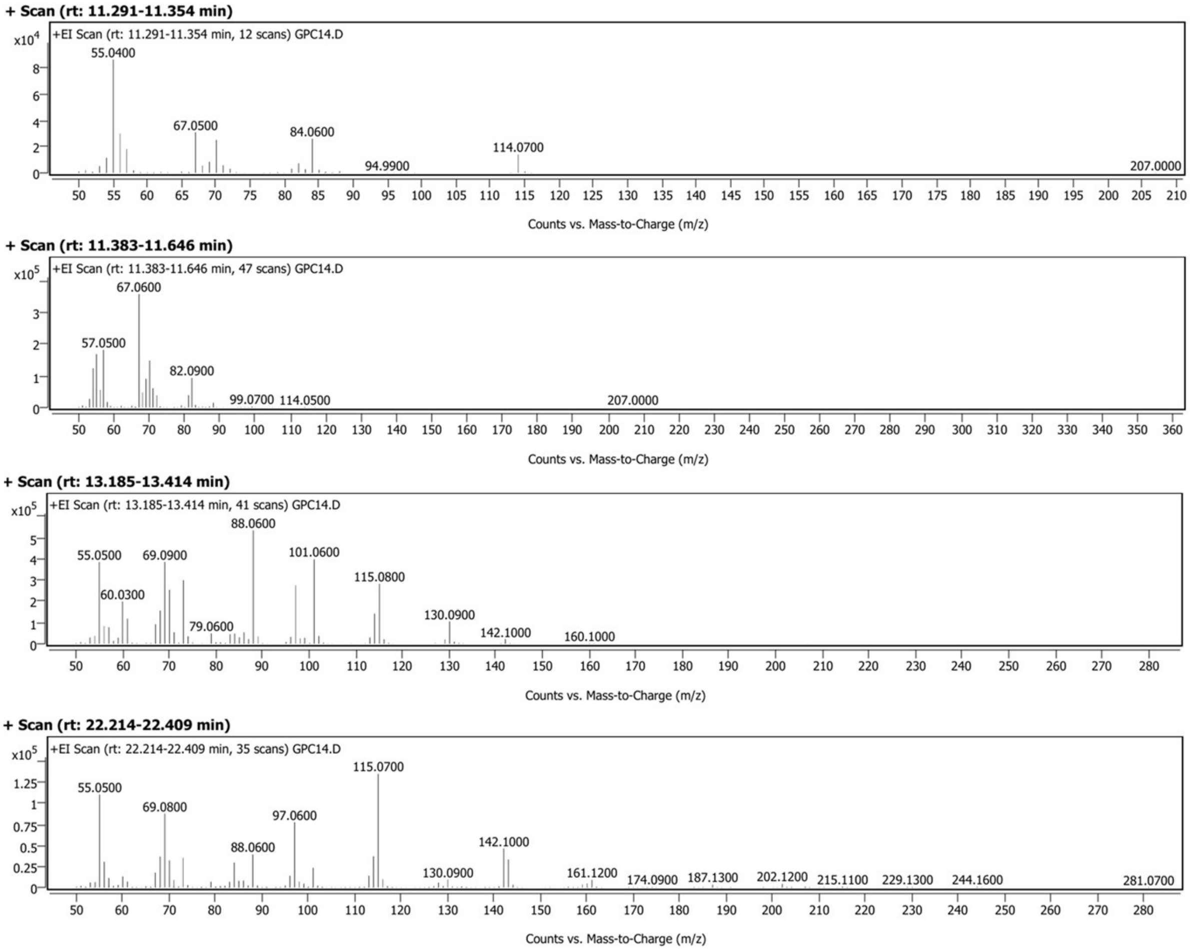

Figure S25. GC-MS data of the reaction mixture resulting from the reaction corresponding to Table S1; Entry 12.

Table S1; Entry 13:

Sample Chromatograms

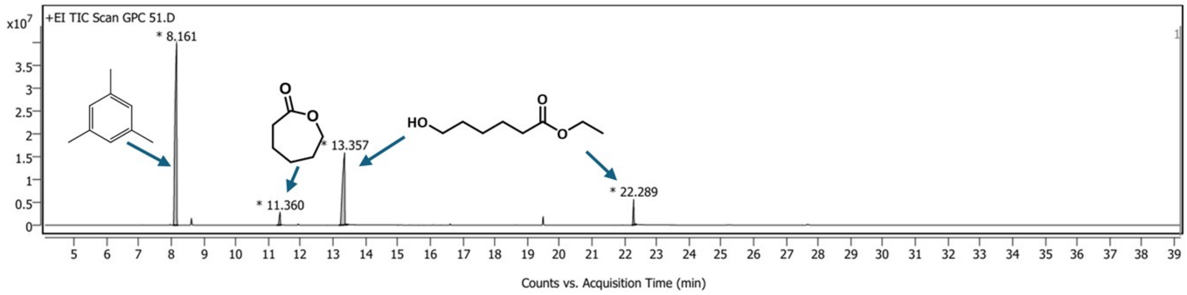

Chromatogram Peaks

Peak	Start	RT	End	Height	Area	Area %	SNR
1	8.053	8.161	8.207	39639283	155520799	100.00	
2	11.257	11.360	11.406	2753232	6508300	4.18	
3	13.174	13.357	13.477	15766528	76466297	49.17	
4	22.232	22.289	22.386	5613674	10305655	6.63	

Sample Spectra

+ Scan (rt: 11.280-11.411 min)

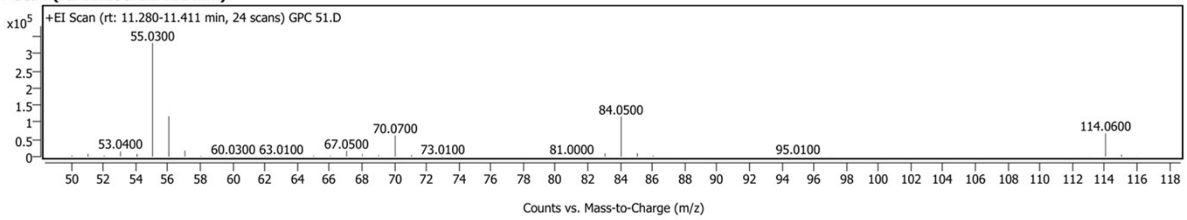

+ Scan (rt: 13.185-13.454 min)

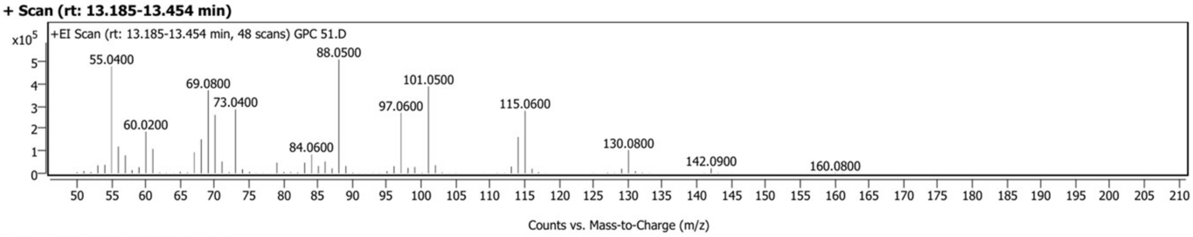

+ Scan (rt: 22.226-22.358 min)

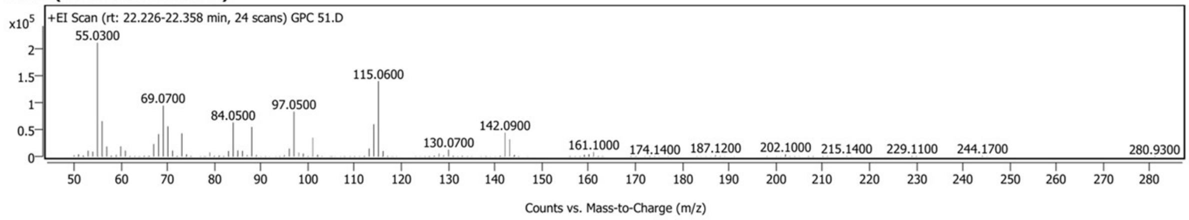

Figure S26. GC-MS data of the reaction mixture resulting from the reaction corresponding to Table S1; Entry 13.

<u>Table S1; Entry 14:</u>

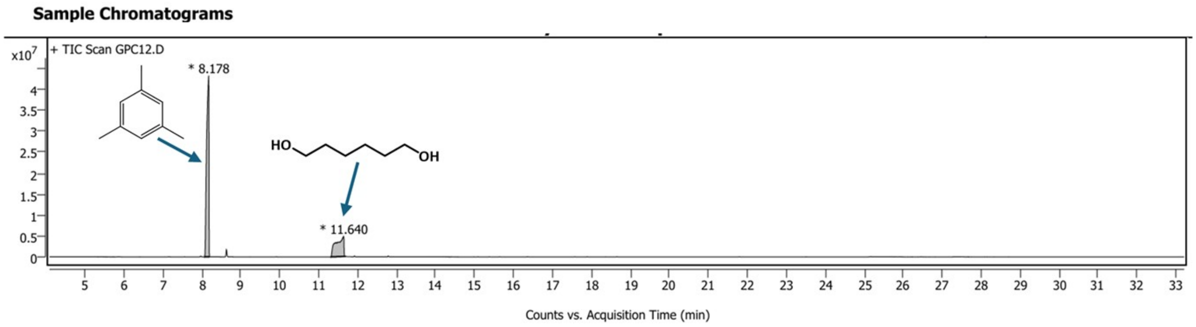

Chromatogram Peaks

Peak	Start	RT	End	Height	Area	Area %	SNR
1	8.058	8.178	8.201	43295006	175663792	100.00	
2	11.303	11.640	11.692	4806349	65612867	37.35	

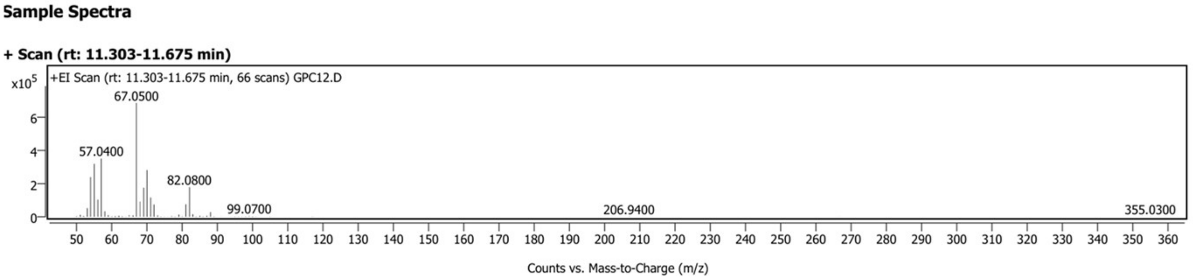

Figure S27. GC-MS data of the reaction mixture resulting from the reaction corresponding to Table S1; Entry 14.

Table S1; Entry 15:

No reaction

Table S1; Entry 16:

Sample Chromatograms

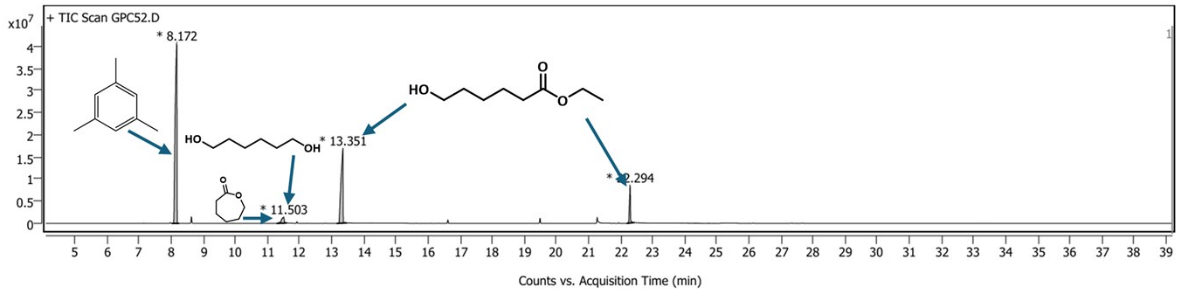

Chromatogram Peaks

Peak	Start	RT	End	Height	Area	Area %	SNR
1	8.024	8.172	8.247	40660705	157650661	100.00	
2	11.285	11.503	11.594	1351029	7918842	5.02	
3	13.156	13.351	13.465	16948097	75236712	47.72	
4	22.192	22.294	22.432	8624638	18423652	11.69	

Sample Spectra

+ Scan (rt: 11.325-11.388 min)

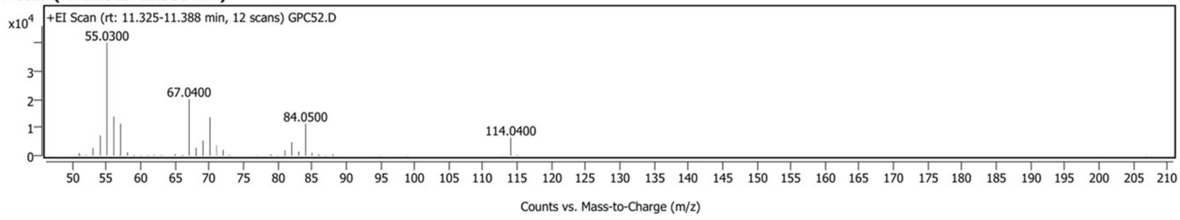

+ Scan (rt: 11.394-11.583 min)

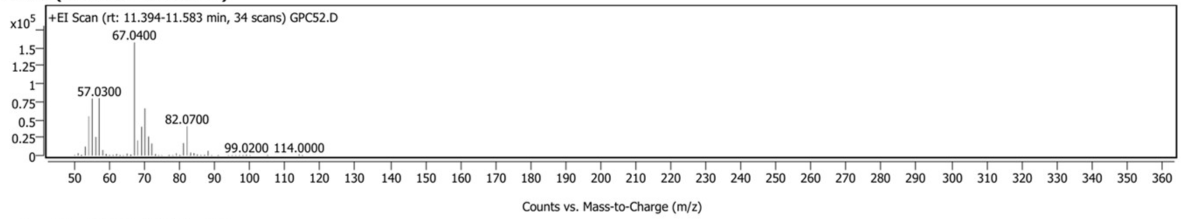

+ Scan (rt: 13.151-13.431 min)

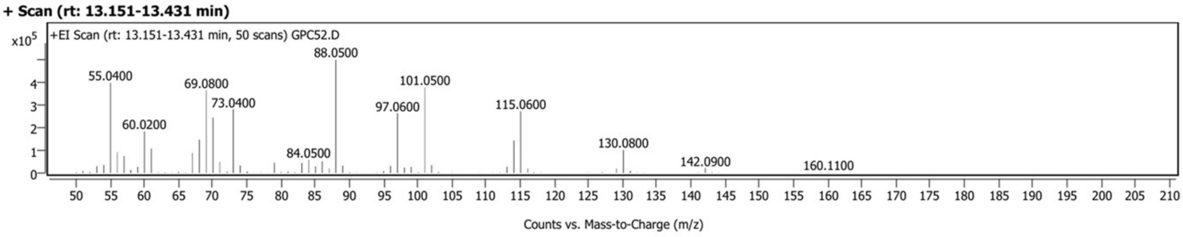

+ Scan (rt: 22.209-22.455 min)

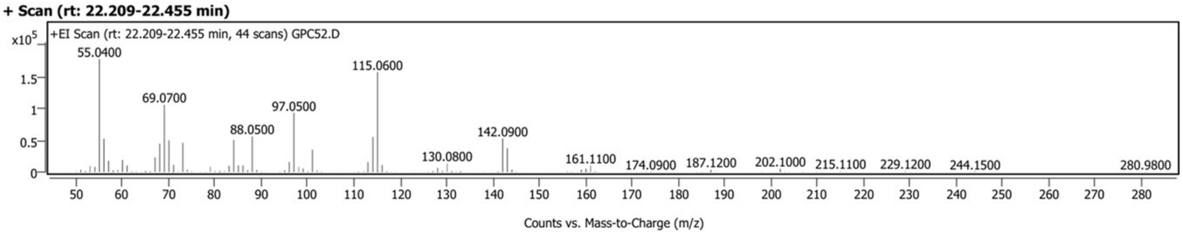

Figure S28. GC-MS data of the reaction mixture resulting from the reaction corresponding to Table S1; Entry 16.

<u>Table S1; Entry 17:</u>

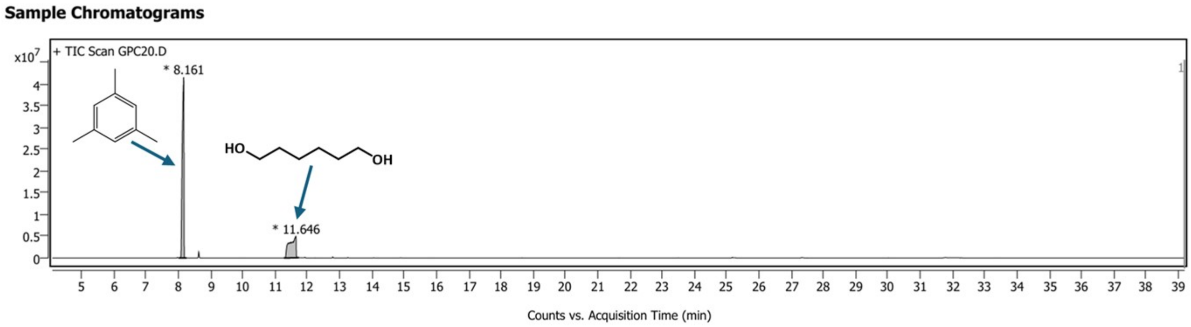

Chromatogram Peaks

Peak	Start	RT	End	Height	Area	Area %	SNR
1	8.018	8.161	8.253	41535130	148803597	100.00	
2	11.274	11.646	11.732	4927668	69102953	46.44	

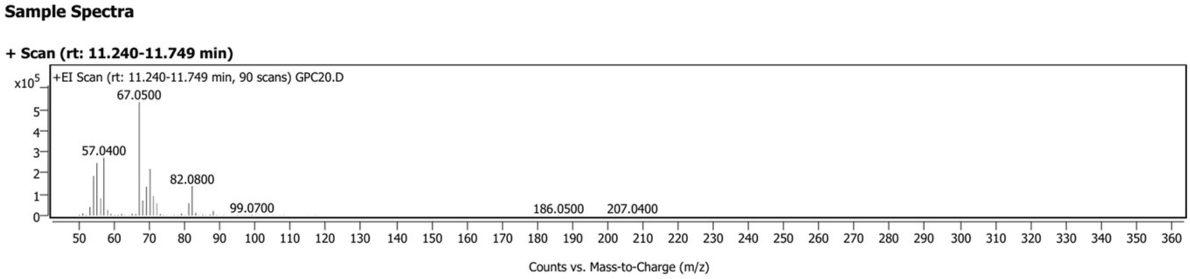

Figure S29. GC-MS data of the reaction mixture resulting from the reaction corresponding to Table S1; Entry 17.

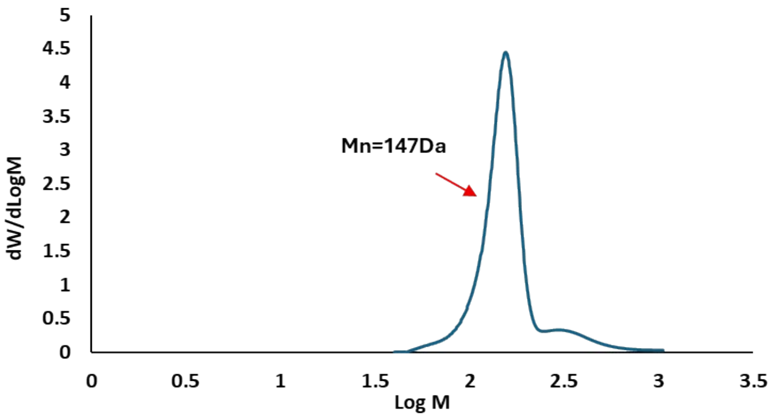

Figure S30. GPC data of the reaction mixture resulting from the reaction corresponding to Table S1; Entry 17.

Table S1; Entry 18:

Sample Chromatograms

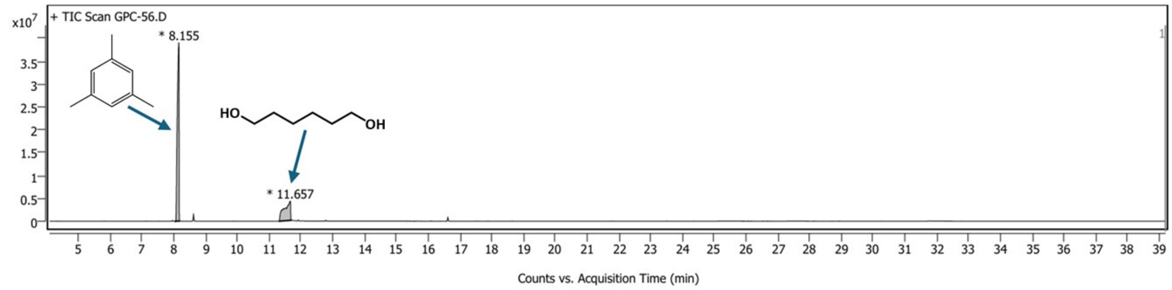

Chromatogram Peaks

Peak	Start	RT	End	Height	Area	Area %	SNR
1	8.030	8.155	8.190	39108909	147519034	100.00	
2	11.303	11.657	11.709	4197514	59107490	40.07	

Sample Spectra

+ Scan (rt: 11.280-11.709 min)

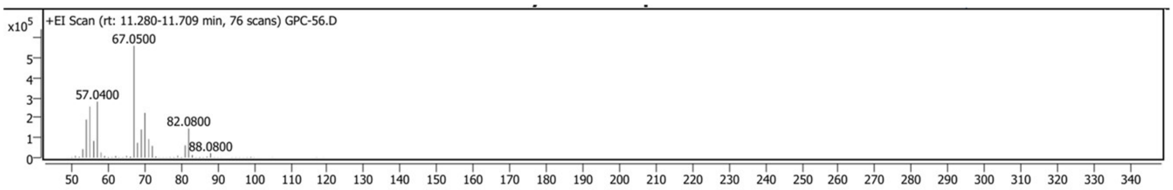

Figure S31. GC-MS data of the reaction mixture resulting from the reaction corresponding to Table S1; Entry 18.

Table S1; Entry 19:

Sample Chromatograms

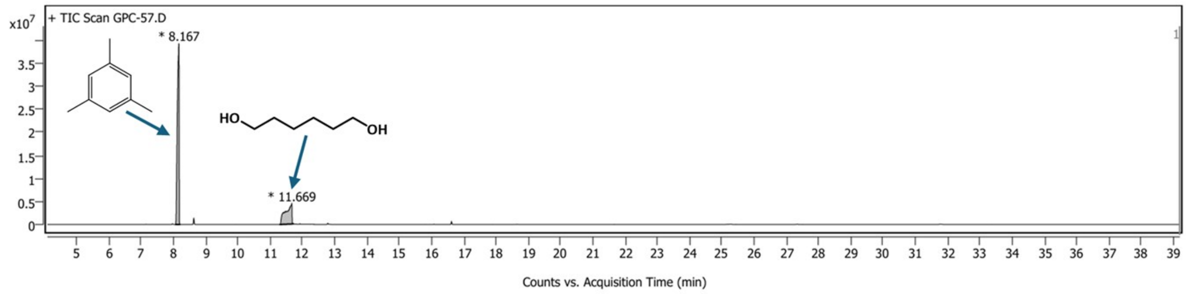

Chromatogram Peaks

Peak	Start	RT	End	Height	Area	Area %	SNR
1	8.053	8.167	8.207	39325088	147568561	100.00	
2	11.291	11.669	11.726	4429319	60697495	41.13	

Sample Spectra

+ Scan (rt: 11.234-11.766 min)

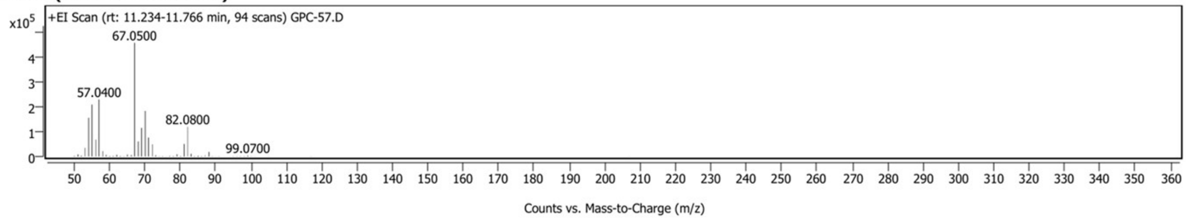

Figure S32. GC-MS data of the reaction mixture resulting from the reaction corresponding to Table S1; Entry 19.

Table S1; Entry 20:

Sample Chromatograms

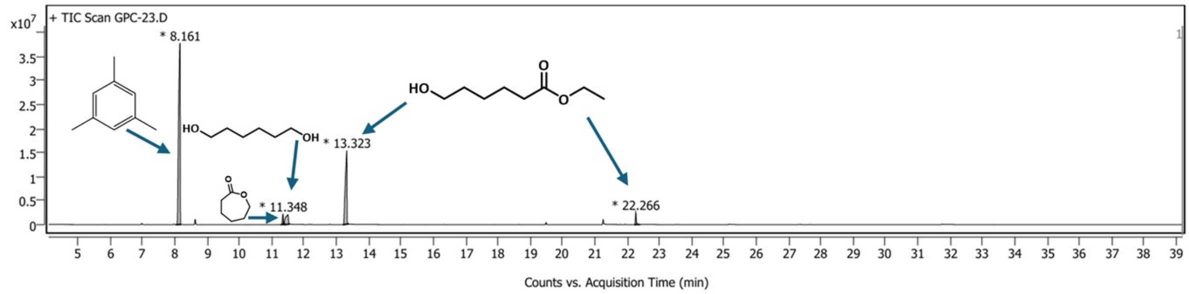

Chromatogram Peaks

Peak	Start	RT	End	Height	Area	Area %	SNR
1	8.030	8.161	8.190	37741086	129133456	100.00	
2	11.274	11.348	11.377	2111143	4124851	3.19	
3	11.377	11.503	11.560	1963029	12899033	9.99	
4	13.202	13.323	13.380	15356743	53880053	41.72	
5	22.226	22.266	22.381	2521330	4469819	3.46	

Sample Spectra

+ Scan (rt: 11.286-11.377 min)

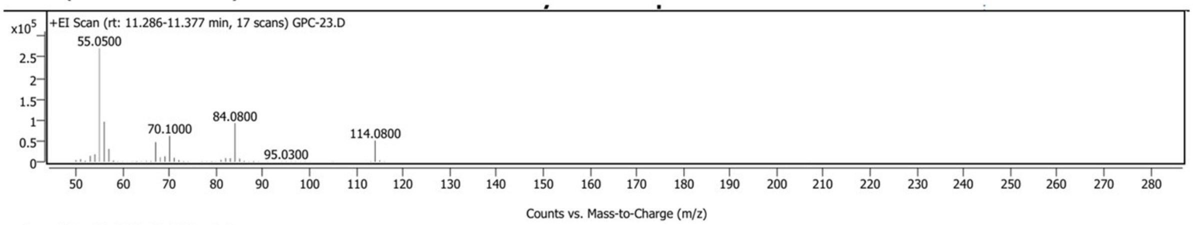

+ Scan (rt: 11.389-11.577 min)

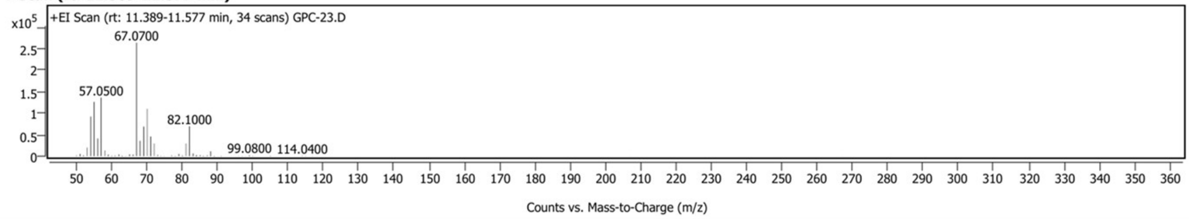

+ Scan (rt: 13.197-13.374 min)

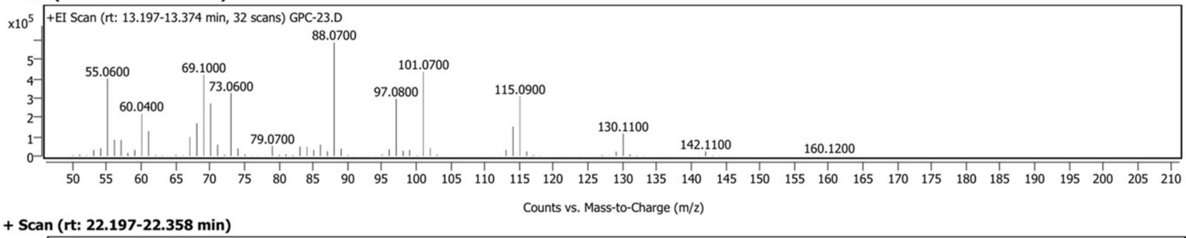

+ Scan (rt: 22.197-22.358 min)

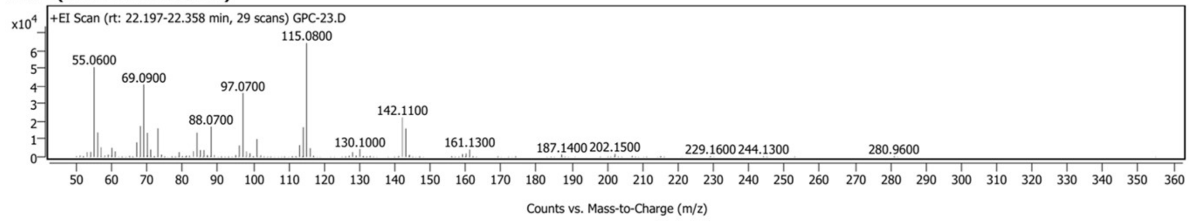

Figure S33. GC-MS data of the reaction mixture resulting from the reaction corresponding to Table S1; Entry 20.

<u>Table S1; Entry 21:</u>

Sample Chromatograms

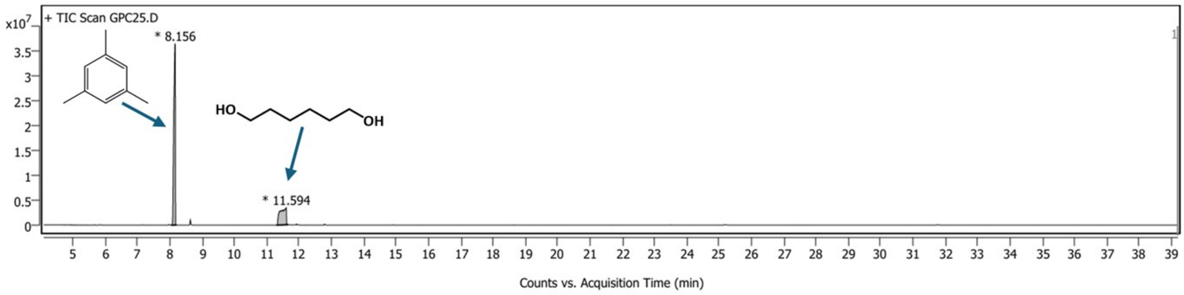

Chromatogram Peaks

Peak	Start	RT	End	Height	Area	Area %	SNR
1	8.035	8.156	8.196	36479353	122054130	100.00	
2	11.297	11.594	11.652	3434485	45789970	37.52	

Sample Spectra

+ Scan (rt: 11.297-11.663 min)

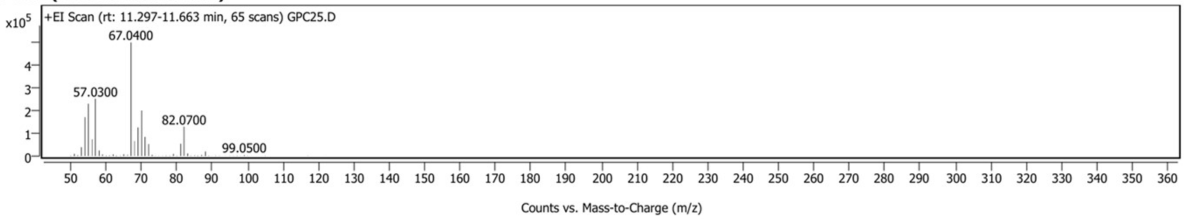

Figure S34. GC-MS data of the reaction mixture resulting from the reaction corresponding to Table S1; Entry 21.

Table S1; Entry 22:

Sample Chromatograms

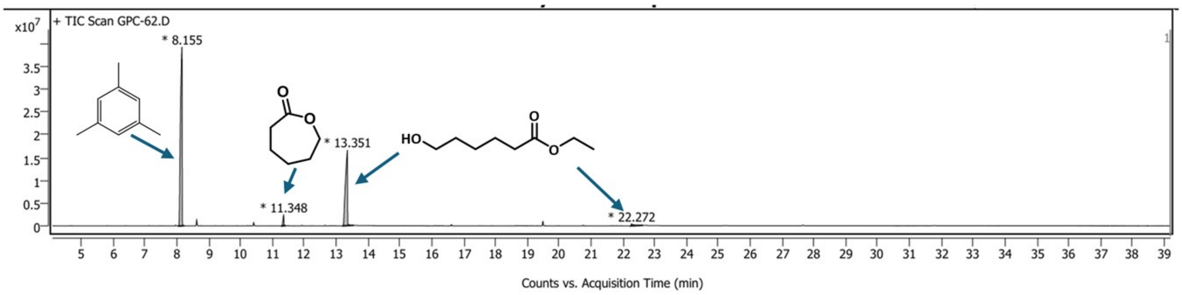

Chromatogram Peaks

Peak	Start	RT	End	Height	Area	Area %	SNR
1	8.035	8.155	8.236	39214484	142279353	100.00	
2	11.268	11.348	11.417	2313512	4630799	3.25	
3	13.191	13.351	13.546	16578279	73944361	51.97	
4	22.243	22.272	22.632	424112	3021531	2.12	

Sample Spectra

+ Scan (rt: 11.291-11.388 min)

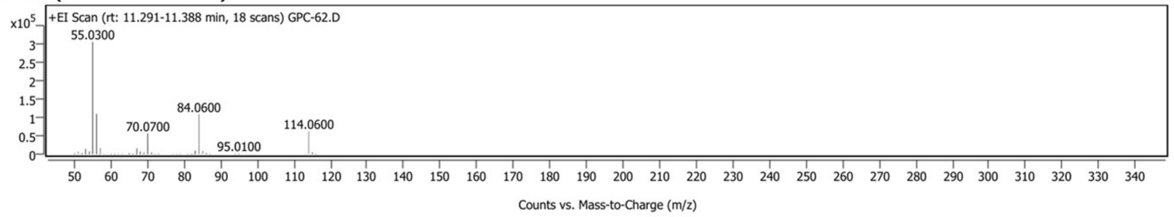

+ Scan (rt: 13.197-13.408 min)

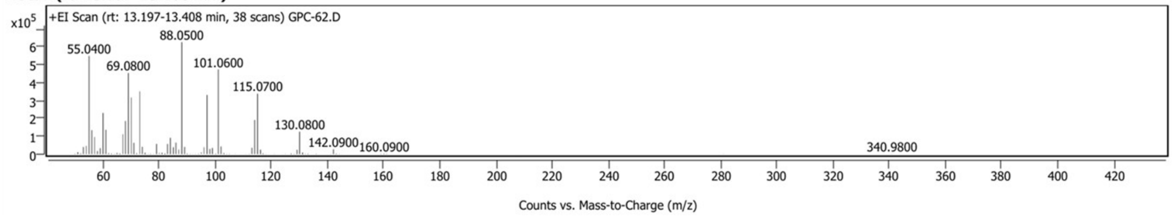

+ Scan (rt: 22.209-22.695 min)

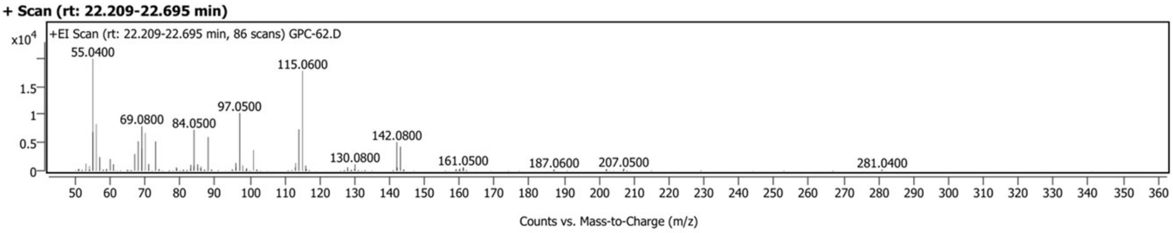

Figure S35. GC-MS data of the reaction mixture resulting from the reaction corresponding to Table S1; Entry 22.

Table S1; Entry 23:

Sample Chromatograms

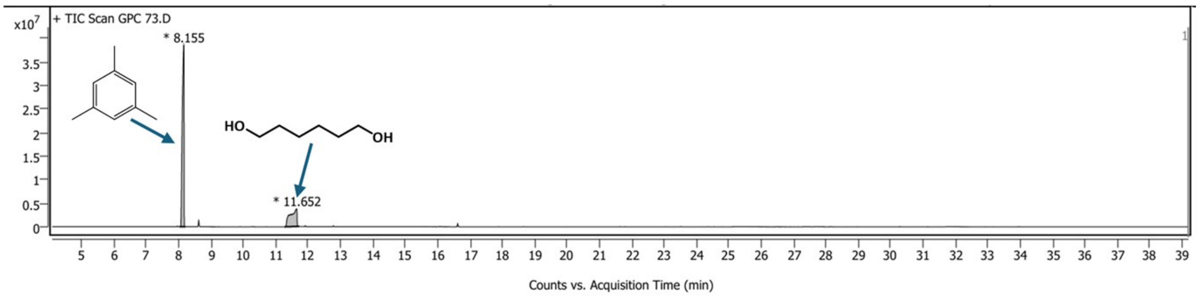

Chromatogram Peaks

Peak	Start	RT	End	Height	Area	Area %	SNR
1	8.030	8.155	8.207	38537338	146130275	100.00	
2	11.274	11.652	11.720	3711150	53255589	36.44	

Sample Spectra

+ Scan (rt: 11.274-11.709 min)

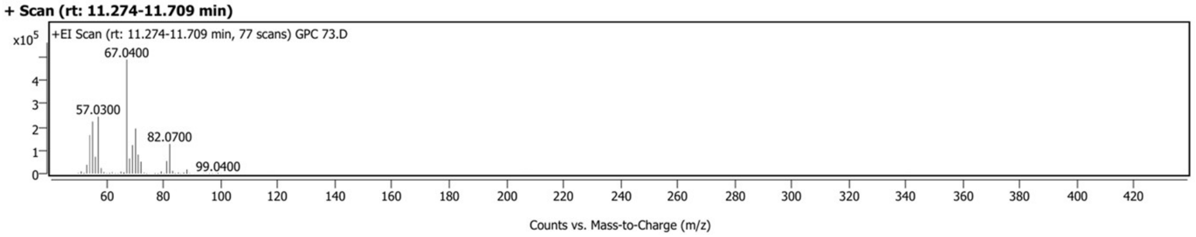

Figure S36. GC-MS data of the reaction mixture resulting from the reaction corresponding to Table S1; Entry 23.

Table S1; Entry 24:

^{1}H NMR (500 MHz, CD_2Cl_2): δH 3.57 (t, HO-**CH_2**-CH_2-CH_2-CH_2-CH_2-**CH_2**-OH), 1.57 (t, HO-CH_2-**CH_2**-CH_2-CH_2-**CH_2**-CH_2-OH), 1.40 (t, HO-CH_2-CH_2-**CH_2-CH_2**-CH_2-CH_2-OH).

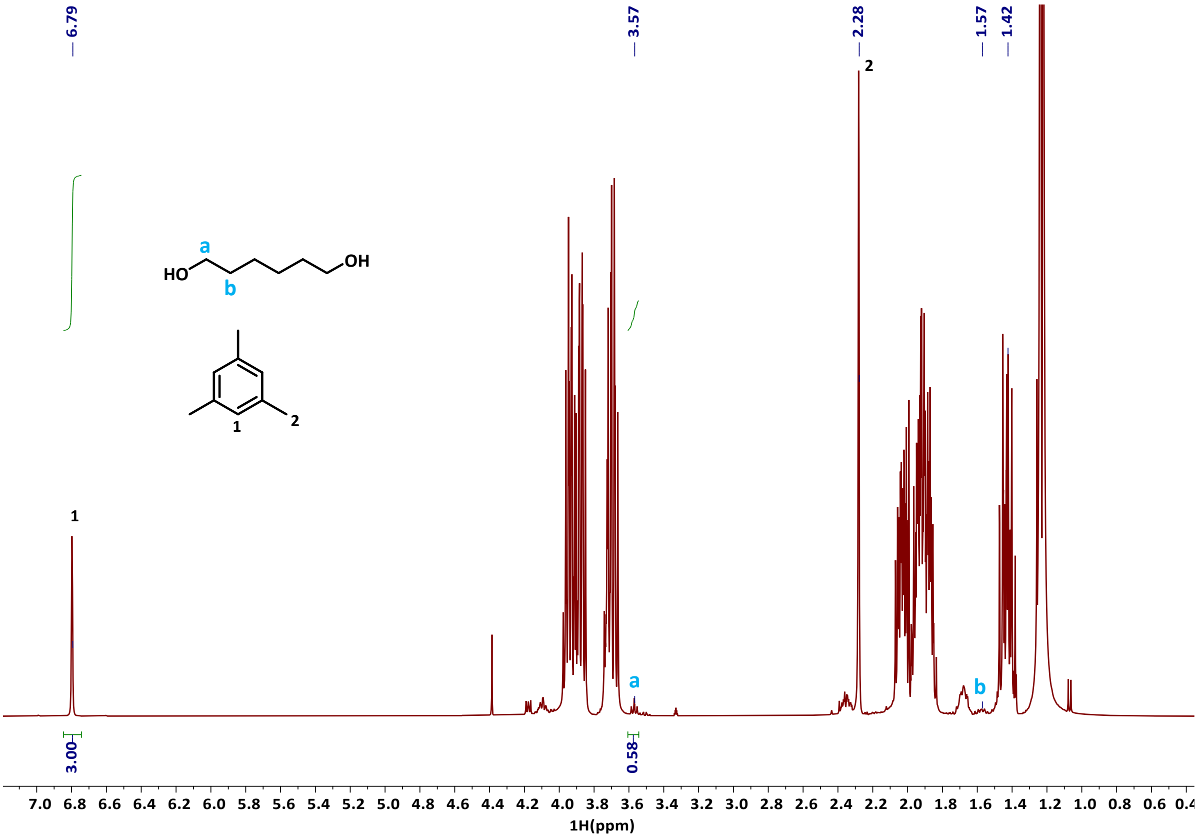

Figure S37. ^1H NMR (400 MHz, CD_3OD) spectrum of the reaction mixture resulting from the reaction corresponding to Table S1; Entry 24. Mesitylene is used as an internal standard.

Table S1; Entry 25:

Sample Chromatograms

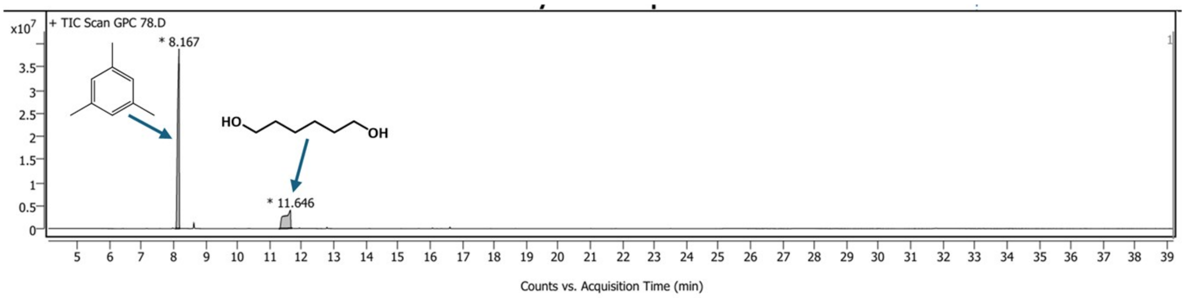

Chromatogram Peaks

Peak	Start	RT	End	Height	Area	Area %	SNR
1	8.053	8.167	8.201	38902772	141019346	100.00	
2	11.286	11.646	11.703	3917085	54139389	38.39	

Sample Spectra

+ Scan (rt: 11.257-11.697 min)

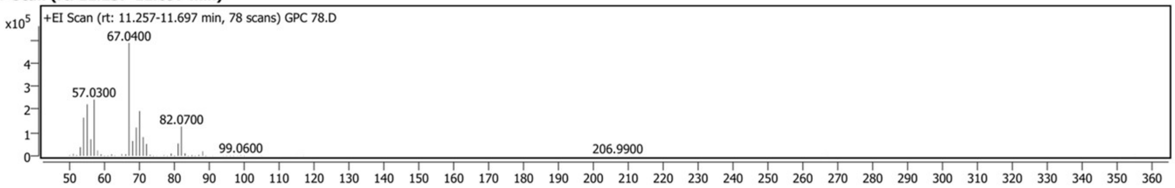

Figure S38. GC-MS data of the reaction mixture resulting from the reaction corresponding to Table S1; Entry 25.

Table S1; Entry 26:

Sample Chromatograms

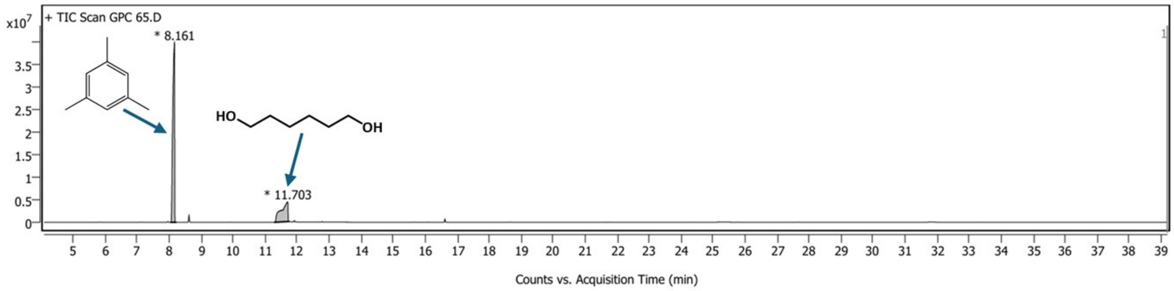

Chromatogram Peaks

Peak	Start	RT	End	Height	Area	Area %	SNR
1	8.041	8.161	8.224	39895552	161903261	100.00	
2	11.285	11.703	11.755	4330019	65788540	40.63	

Sample Spectra

+ Scan (rt: 11.285-11.755 min)

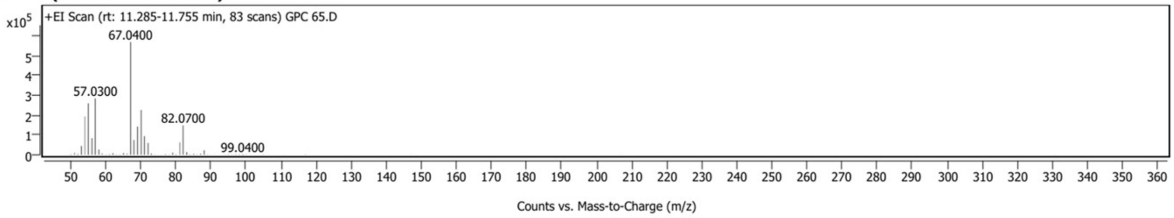

Figure S39. GC-MS data of the reaction mixture resulting from the reaction corresponding to Table S1; Entry 26.

Table S1; Entry 27:

Sample Chromatograms

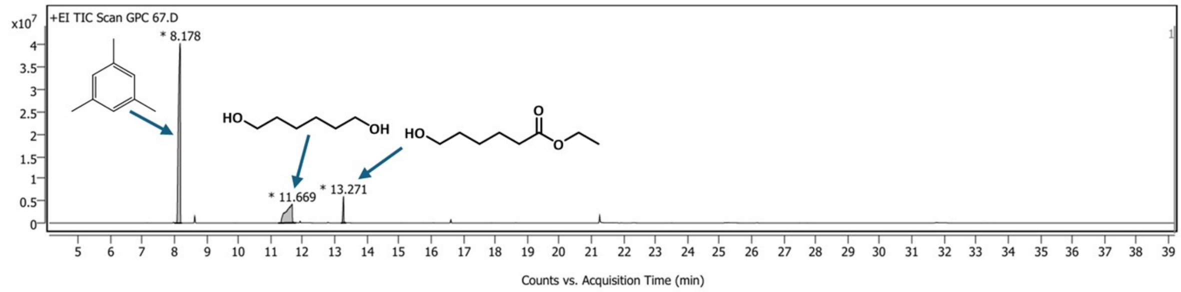

Chromatogram Peaks

Peak	Start	RT	End	Height	Area	Area %	SNR
1	8.001	8.178	8.230	40431049	166352914	100.00	
2	11.234	11.669	11.789	4155666	58284532	35.04	
3	13.191	13.271	13.351	5946552	11195951	6.73	

Sample Spectra

+ Scan (rt: 11.251-11.777 min)

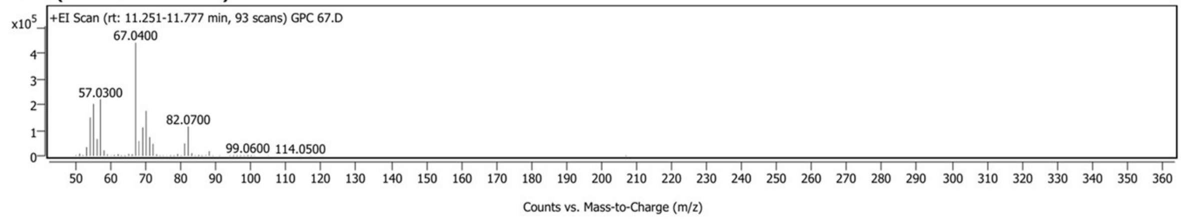

+ Scan (rt: 13.145-13.443 min)

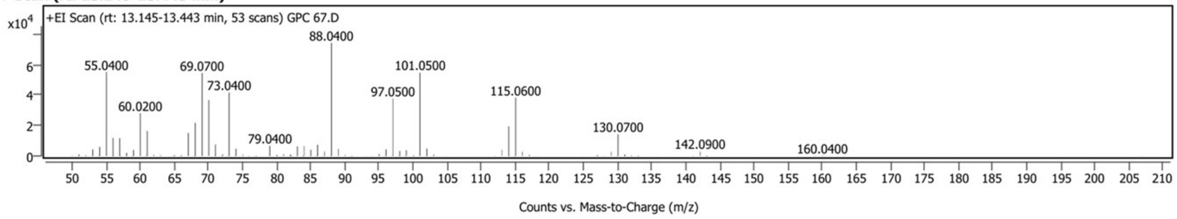

Figure S40. GC-MS data of the reaction mixture resulting from the reaction corresponding to Table S1; Entry 27.

9. Optimisation of Catalytic Conditions under solvent minimised conditions

Table S2. Optimisation of catalytic conditions for the hydrogenolysis of Polycaprolactone under solvent minimised conditions.

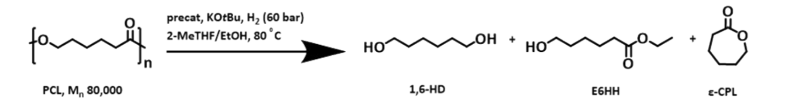

Entry	Precatalyst	Precatalyst loading (mol%)	KOtBu (mol%)	EtOH (mL)	Time,h	1,6-HD (%)	EHH (%)	ε-CPL (%)	TON[b]
1	8	0.05	5	0.25	20	91	0	0	1,820
2	8	0.01	5	0.25	72	96	0	0	9,600
3	8	0.005	5	0.25	72	88	0	0	17,600
4	7	0.05	5	0.25	20	96	0	0	1,920
5	7	0.01	5	0.25	72	99	0	0	9,900
6	7	0.005	5	0.25	72	98	0	0	19,600
7[c]	8	0.005	5	1.25	72	92	0	0	18,400
8[c]	7	0.005	5	1.25	72	95	0	0	19,000

[a]Polycaprolactone M_n = 80,000 Da (1 mmol of monomeric unit), EtOH, KO*t*Bu, precatalyst 8 (2,5 mM solution in 2-Me THF), precatalyst 7 (2 mM solution in EtOH) 80 °C, 60 bar H_2. Yields were estimated by quantitive GC-MS analysis using mesitylene as an internal standard. [b]TON estimated based on the number of ester groups hydrogenated. [c] 5 mmol of PCL used (relative to the monomeric unit).

Table S2; Entry 1:

Sample Chromatograms

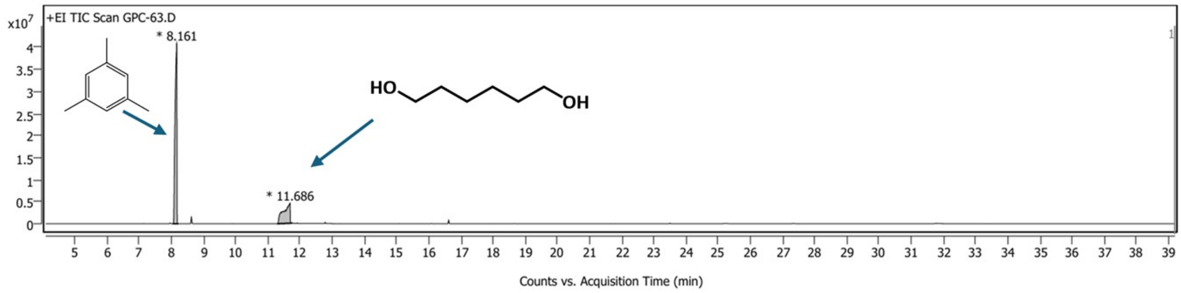

Chromatogram Peaks

Peak	Start	RT	End	Height	Area	Area %	SNR
1	8.041	8.161	8.213	40929581	161982599	100.00	
2	11.291	11.686	11.761	4580198	64728375	39.96	

Sample Spectra

+ Scan (rt: 11.269-11.755 min)

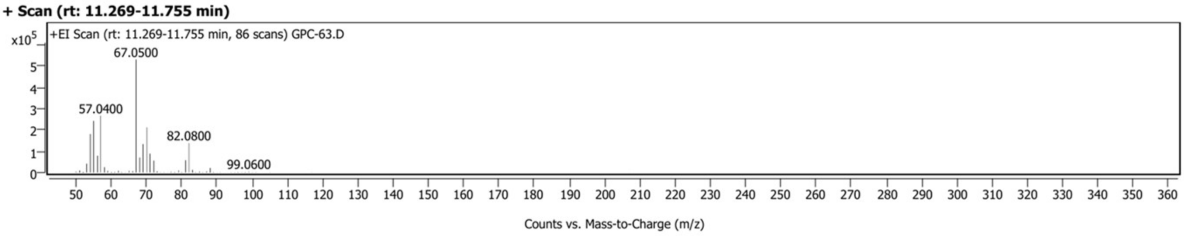

Figure S41. GC-MS data of the reaction mixture resulting from the reaction corresponding to Table S2; Entry 1.

Table S2; Entry 2:

Sample Chromatograms

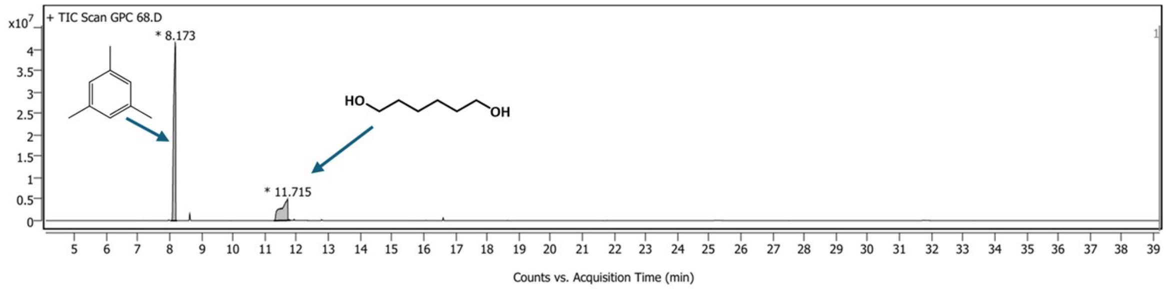

Chromatogram Peaks

Peak	Start	RT	End	Height	Area	Area %	SNR
1	8.036	8.173	8.224	41446431	171677594	100.00	
2	11.286	11.715	11.806	4956105	74085299	43.15	

Sample Spectra

+ Scan (rt: 11.148-11.795 min)

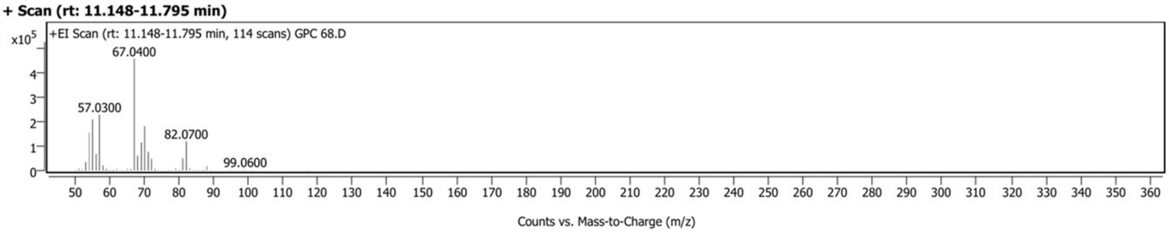

Figure S42. GC-MS data of the reaction mixture resulting from the reaction corresponding to Table S2; Entry 2.

Table S2; Entry 3:

Sample Chromatograms

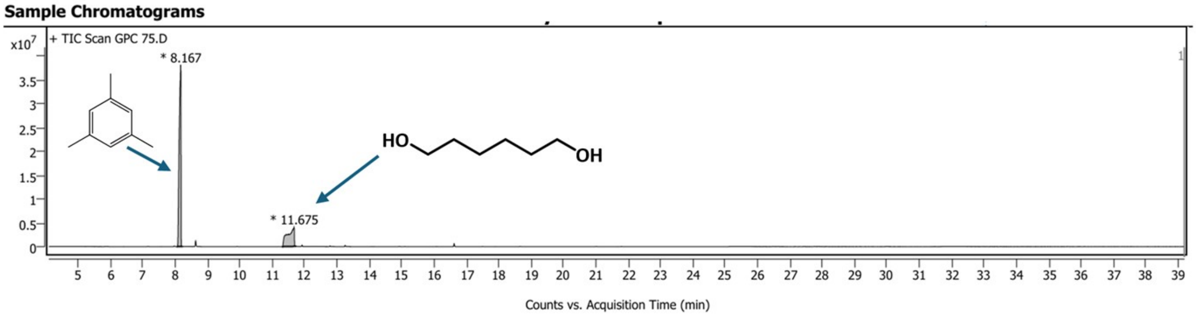

Chromatogram Peaks

Peak	Start	RT	End	Height	Area	Area %	SNR
1	8.047	8.167	8.236	38224800	143597503	100.00	
2	11.297	11.675	11.737	4019607	56634694	39.44	

Sample Spectra

+ Scan (rt: 11.177-11.766 min)

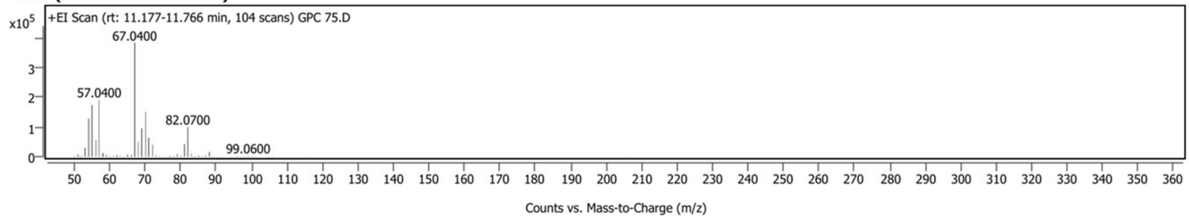

Figure S43. GC-MS data of the reaction mixture resulting from the reaction corresponding to Table S2; Entry 3.

<u>Table S2; Entry 4:</u>

Sample Chromatograms

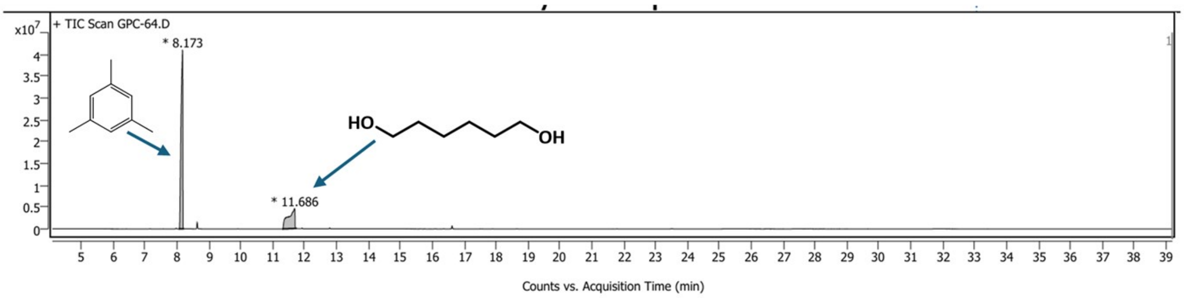

Chromatogram Peaks

Peak	Start	RT	End	Height	Area	Area %	SNR
1	8.058	8.173	8.213	41020169	156609490	100.00	
2	11.303	11.686	11.743	4563893	65893795	42.08	

Sample Spectra

+ Scan (rt: 11.303-11.743 min)

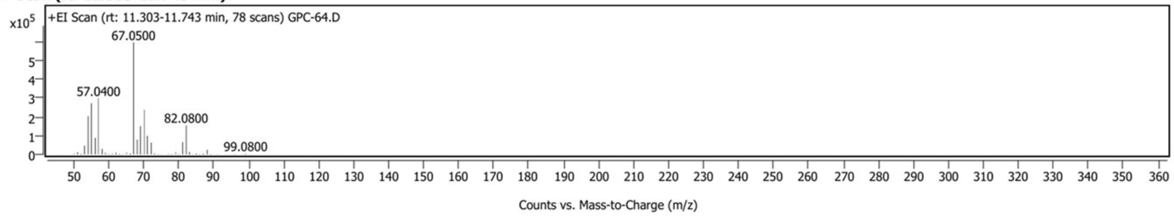

Figure S44. GC-MS data of the reaction mixture resulting from the reaction corresponding to Table S2; Entry 4.

<u>Table S2; Entry 5:</u>

Sample Chromatograms

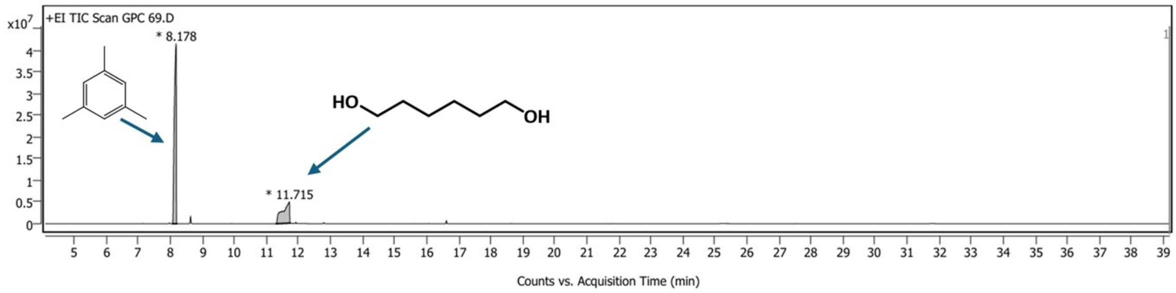

Chromatogram Peaks

Peak	Start	RT	End	Height	Area	Area %	SNR
1	8.052	8.178	8.213	41675303	175817702	100.00	
2	11.285	11.715	11.755	4904951	74840221	42.57	

Sample Spectra

+ Scan (rt: 11.205-11.846 min)

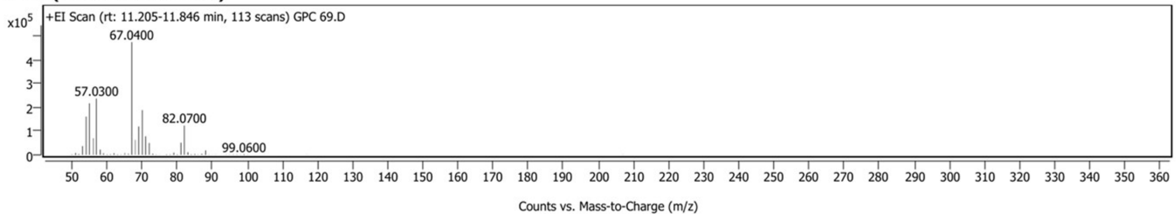

Figure S45. GC-MS data of the reaction mixture resulting from the reaction corresponding to Table S2; Entry 5.

<u>Table S2; Entry 6</u>

Sample Chromatograms

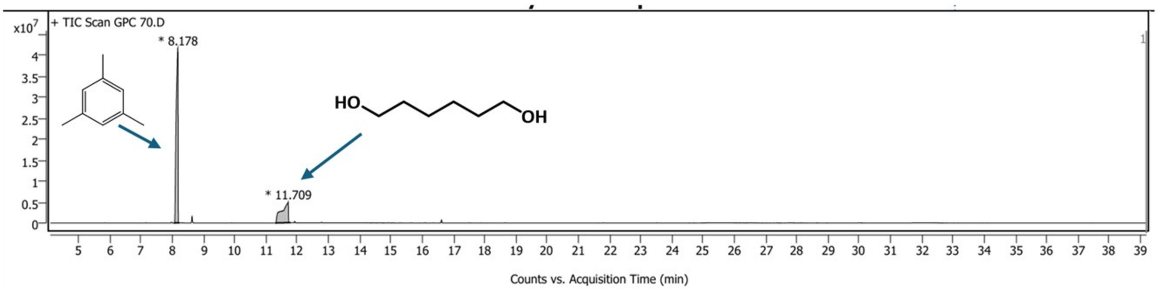

Chromatogram Peaks

Peak	Start	RT	End	Height	Area	Area %	SNR
1	8.052	8.178	8.224	41850944	170779075	100.00	
2	11.303	11.709	11.783	4935390	73447954	43.01	

Sample Spectra

+ Scan (rt: 11.091-11.846 min)

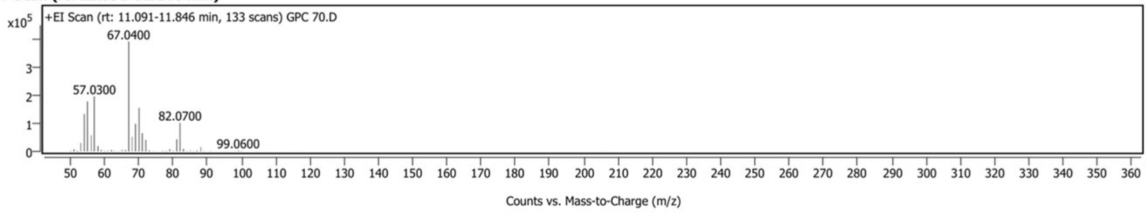

Figure S46. GC-MS data of the reaction mixture resulting from the reaction corresponding to Table S2; Entry 6.

<u>Table S2; Entry 7:</u>

Sample Chromatograms

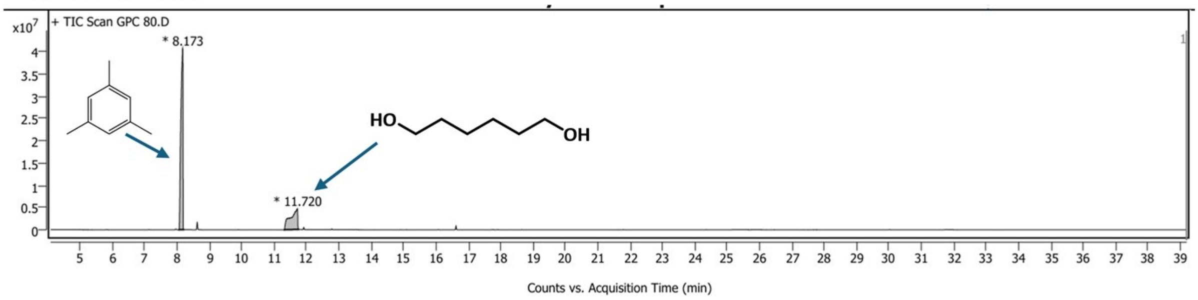

Chromatogram Peaks

Peak	Start	RT	End	Height	Area	Area %	SNR
1	8.041	8.173	8.218	40771412	181388791	100.00	
2	11.297	11.720	11.772	4620213	71810857	39.59	

Sample Spectra

+ Scan (rt: 11.263-11.846 min)

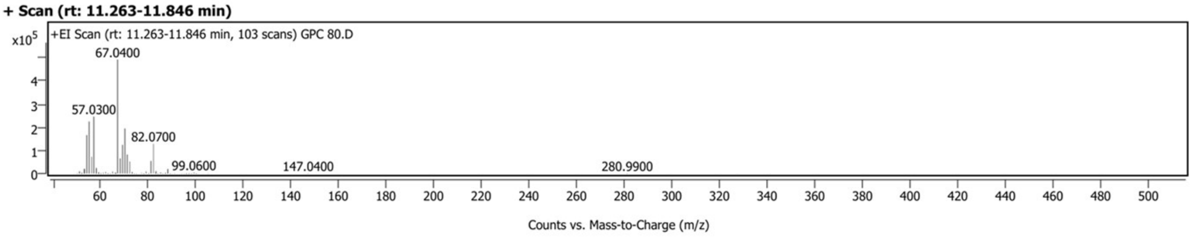

Figure S47. GC-MS data of the reaction mixture resulting from the reaction corresponding to Table S2; Entry 7.

<u>Table S2; Entry 8:</u>

Sample Chromatograms

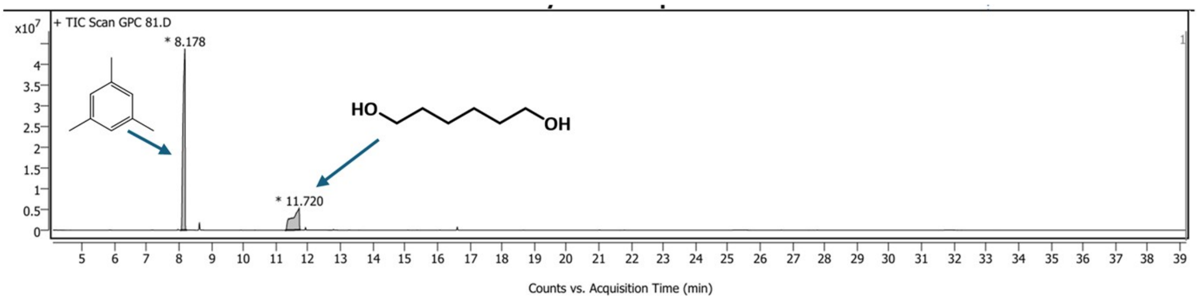

Chromatogram Peaks

Peak	Start	RT	End	Height	Area	Area %	SNR
1	8.041	8.178	8.247	43678628	192404961	100.00	
2	11.286	11.720	11.766	5178973	78720999	40.91	

Sample Spectra

+ Scan (rt: 11.286-11.789 min)

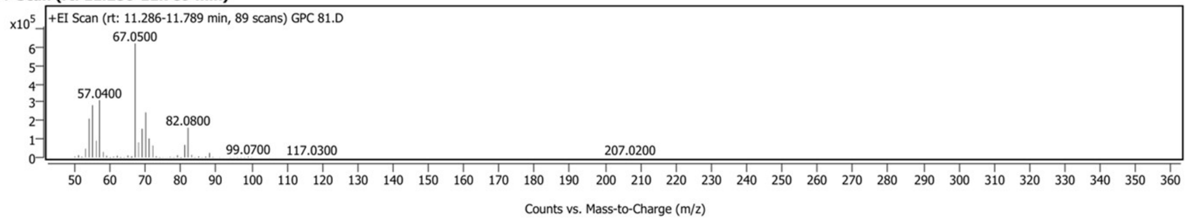

Figure S48. GC-MS data of the reaction mixture resulting from the reaction corresponding to Table S2; Entry 8.

10. Kinetic studies

Kinetic analysis of the depolymerisation of Polycaprolactone without precatalyst and Hydrogen pressure:

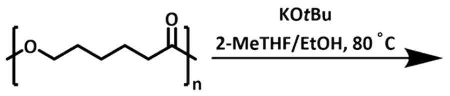
KOtBu
2-MeTHF/EtOH, 80 ˚C

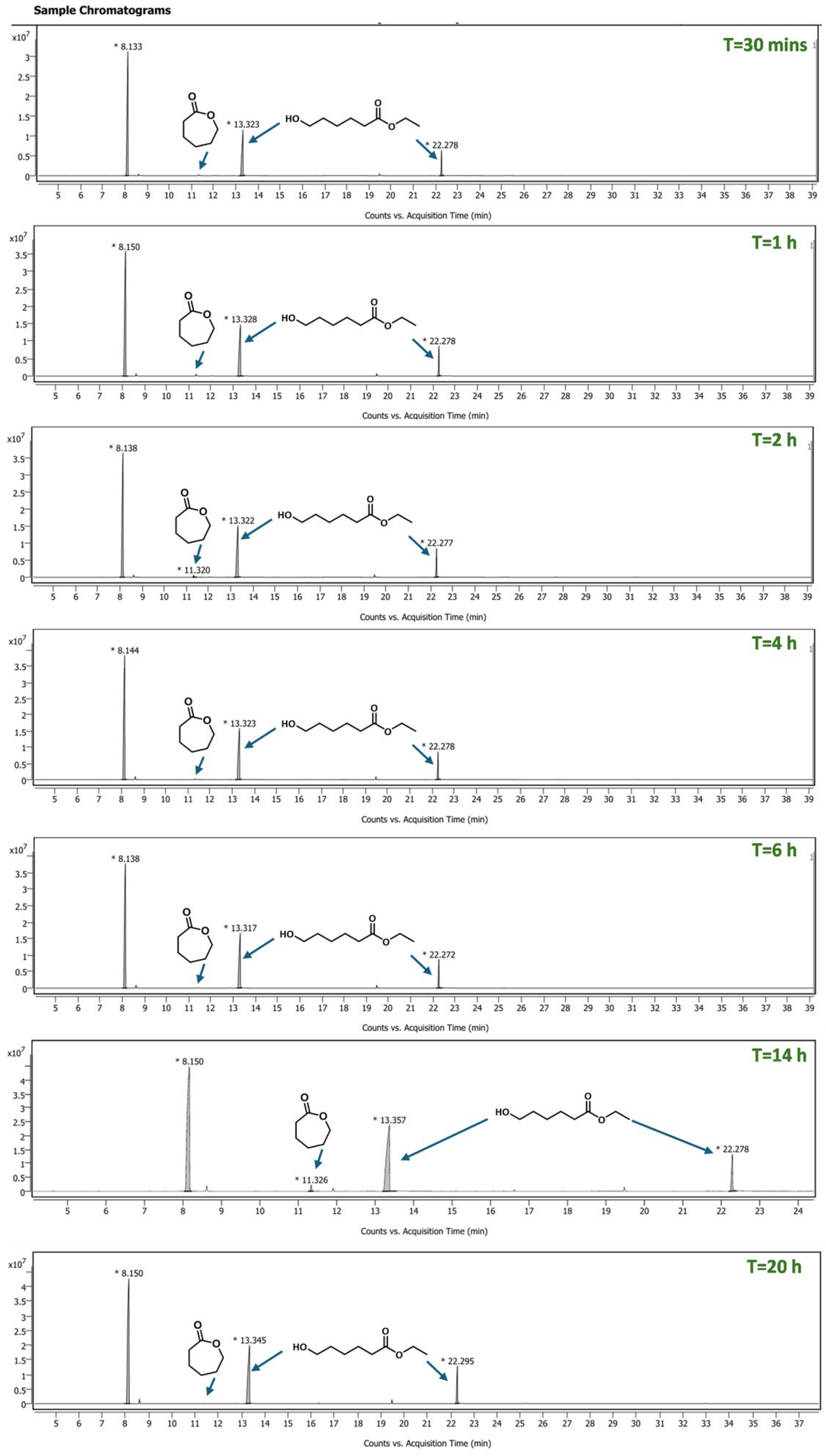
Sample Chromatograms
T=30 mins
* 8.133
* 13.323
* 22.278
T=1 h
* 8.150
* 13.328
* 22.278
T=2 h
* 8.138
* 13.322
* 11.320
* 22.277
T=4 h
* 8.144
* 13.323
* 22.278
T=6 h
* 8.138
* 13.317
* 22.272
T=14 h
* 8.150
* 13.357
* 11.326
* 22.278
T=20 h
* 8.150
* 13.345
* 22.295
HO
Counts vs. Acquisition Time (min)

Figure S49. GC-MS data of the reaction mixtures at different time intervals.

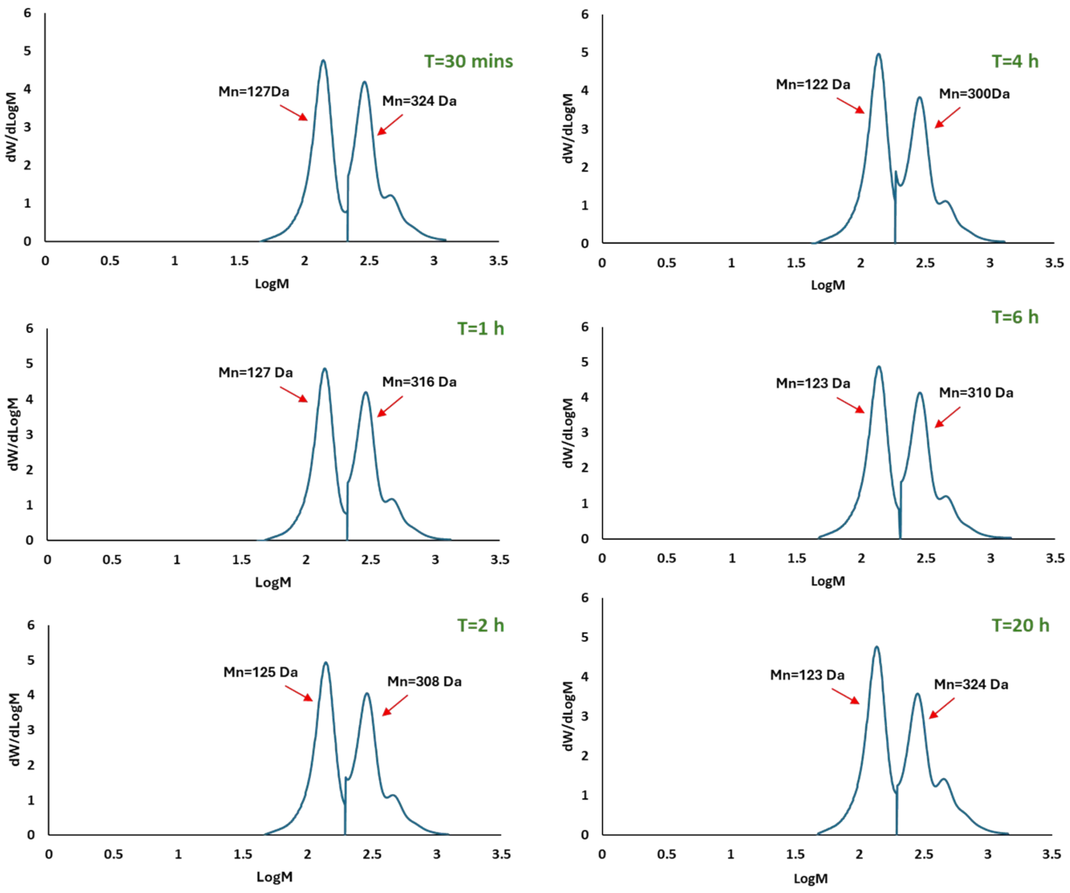

Figure S50. GPC data of the reaction mixtures at different time intervals.

Kinetic analysis of the depolymerisation of Polycaprolactone without Hydrogen pressure:

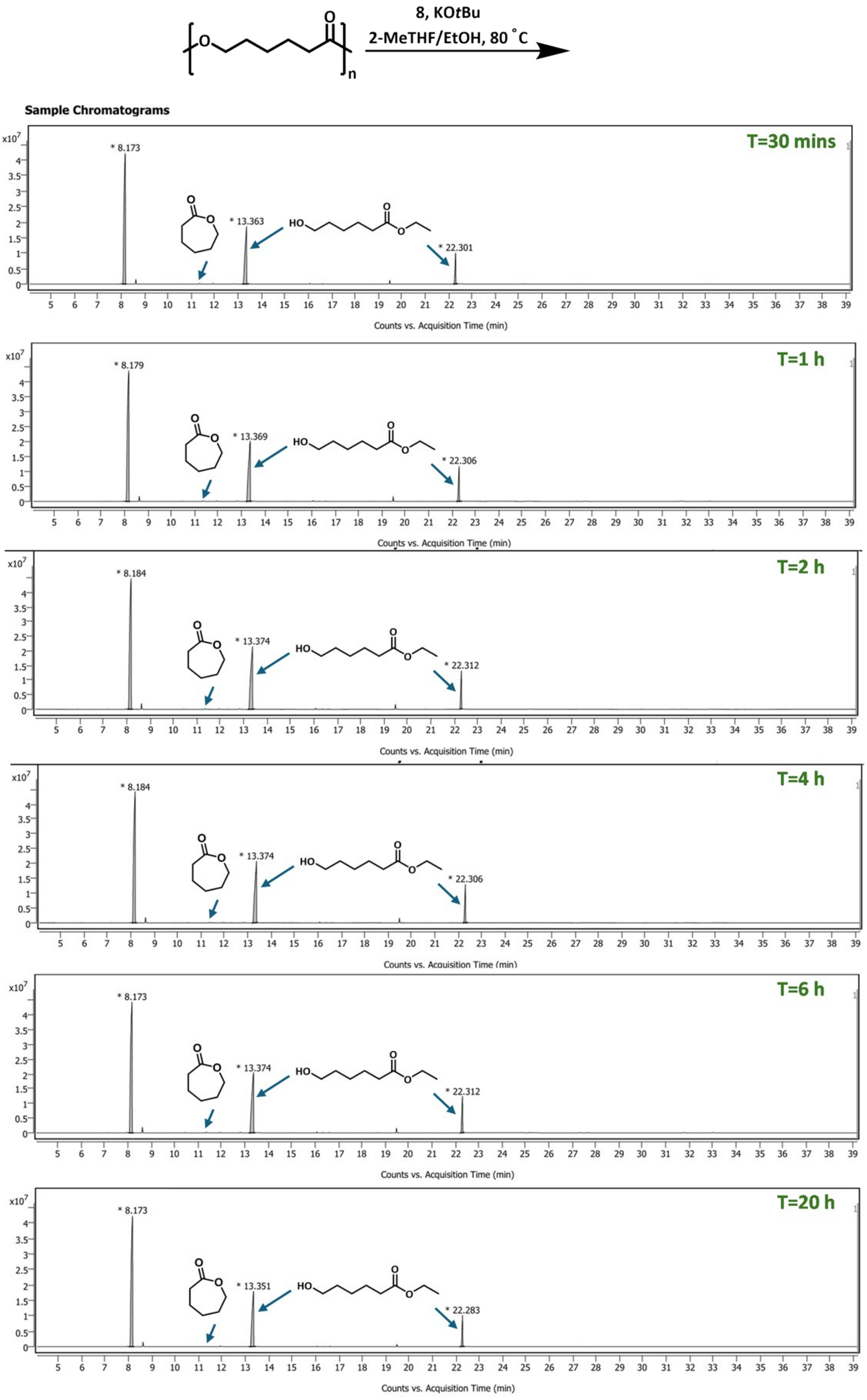

Figure S51. GC-MS data of the reaction mixtures at different time intervals.

Kinetic analysis of the depolymerisation of Polycaprolactone with Hydrogen pressure:

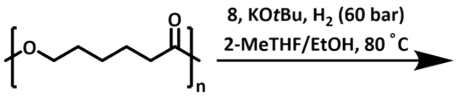
8, KOtBu, H2 (60 bar)
2-MeTHF/EtOH, 80 °C
n

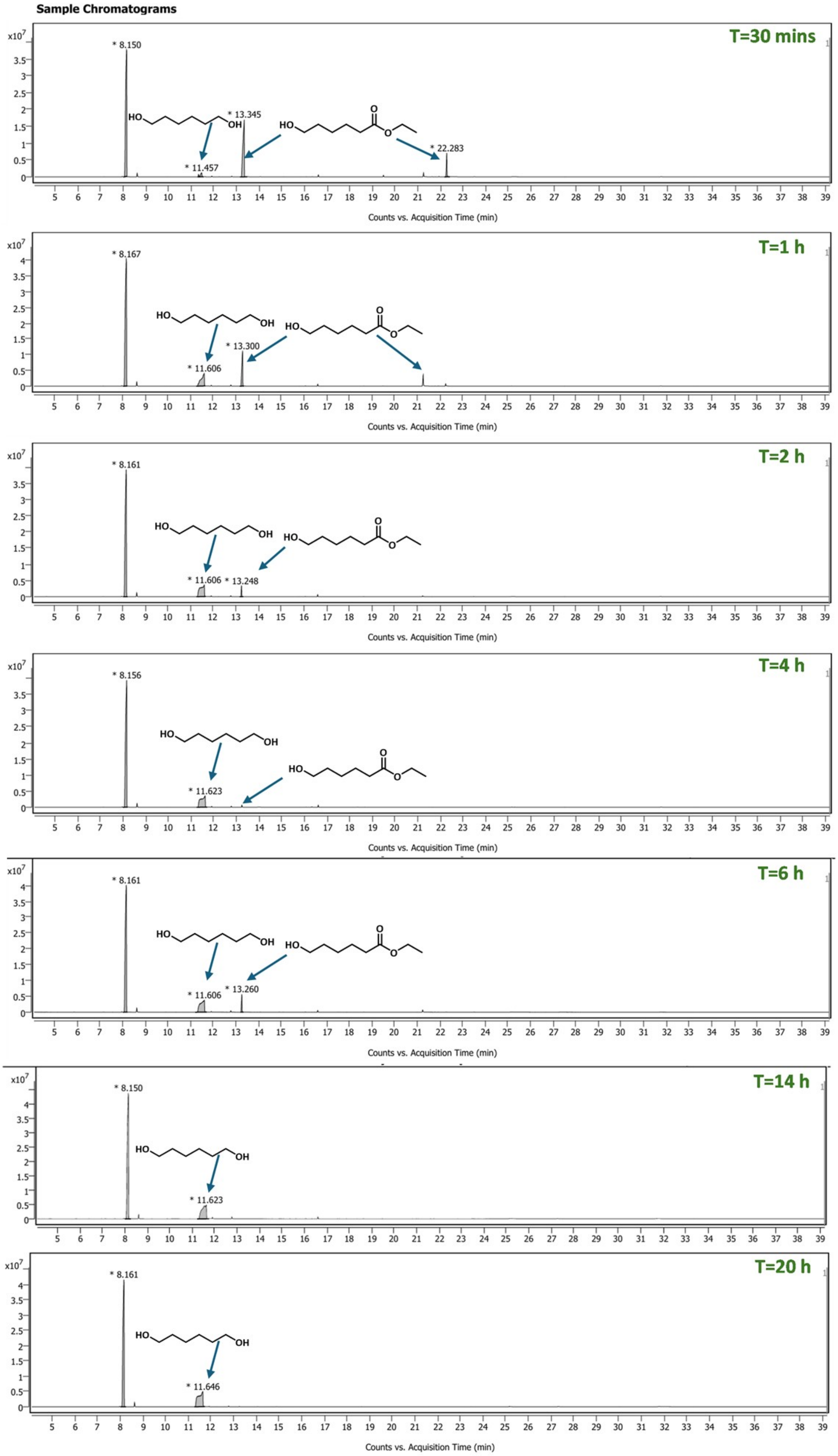
Sample Chromatograms
T=30 mins
* 8.150
* 11.457
* 13.345
* 22.283
T=1 h
* 8.167
* 11.606
* 13.300
T=2 h
* 8.161
* 11.606
* 13.248
T=4 h
* 8.156
* 11.623
T=6 h
* 8.161
* 11.606
* 13.260
T=14 h
* 8.150
* 11.623
T=20 h
* 8.161
* 11.646
HO
OH
x10^7
Counts vs. Acquisition Time (min)

Figure S52. GC-MS data of the reaction mixtures at different time intervals.

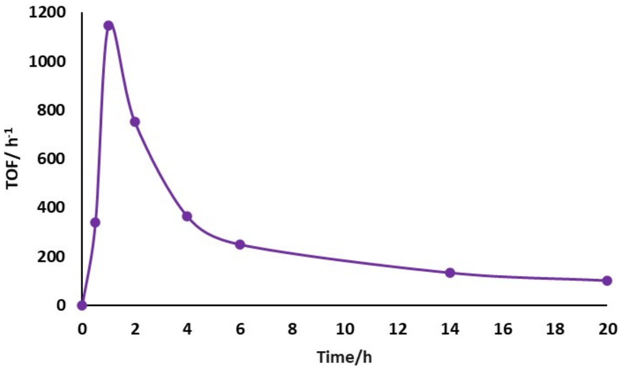

Figure S53. Turnover frequency (TOF) data for the hydrogenation reaction at different time intervals.

11. Reaction with ε-caprolactone

The hydrogenation reaction with ε-caprolactone as substrate was conducted instead of PCL using the same reaction conditions as discussed in Table S1, entry 17, and it resulted in full transformation to 1,6-hexanediol confirmed by the GC-MS.

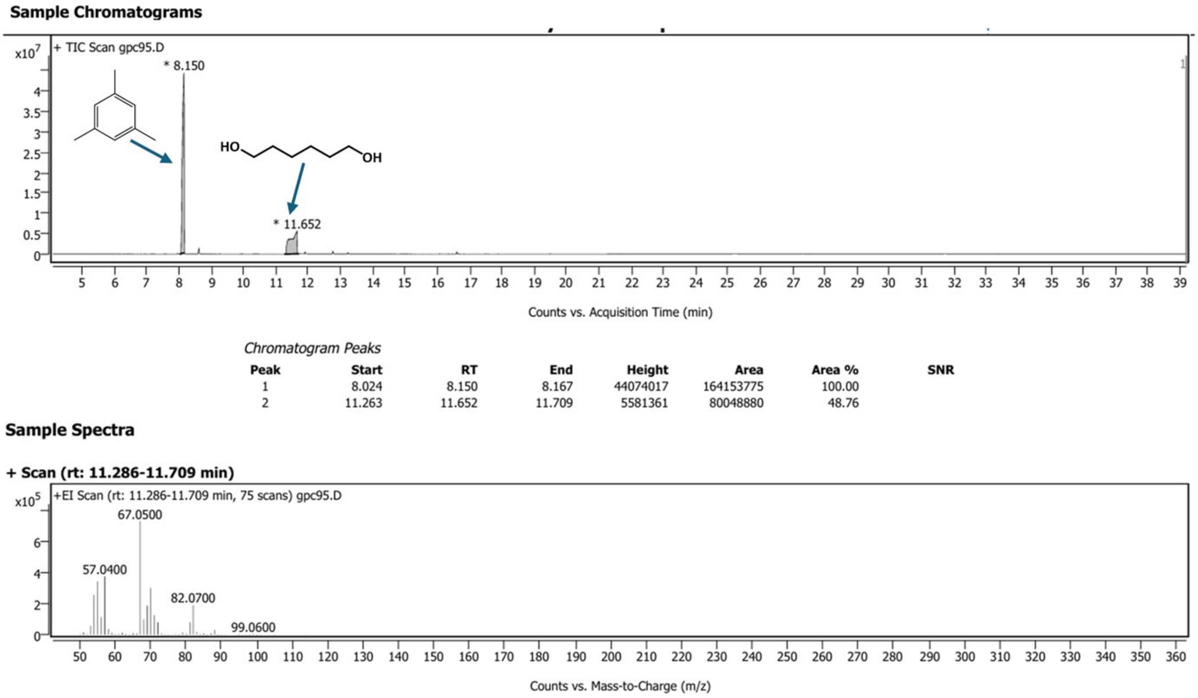

Figure S54. GC-MS data of the reaction mixture resulting from the reaction with ε-caprolactone.

12. Role of alcohols in transesterification reactions

The transesterification reactions for different alcohols using the same reaction conditions for 20 h, as described in Fig. 4a in the manuscript were performed. Reaction with methanol resulted in 76% yield of methyl 6-hydroxyhexanoate, and isopropanol resulted in 52% yield of isopropyl 6-hydroxyhexanoate. These suggest that transesterification process can work with multiple alcohols in similar ways.

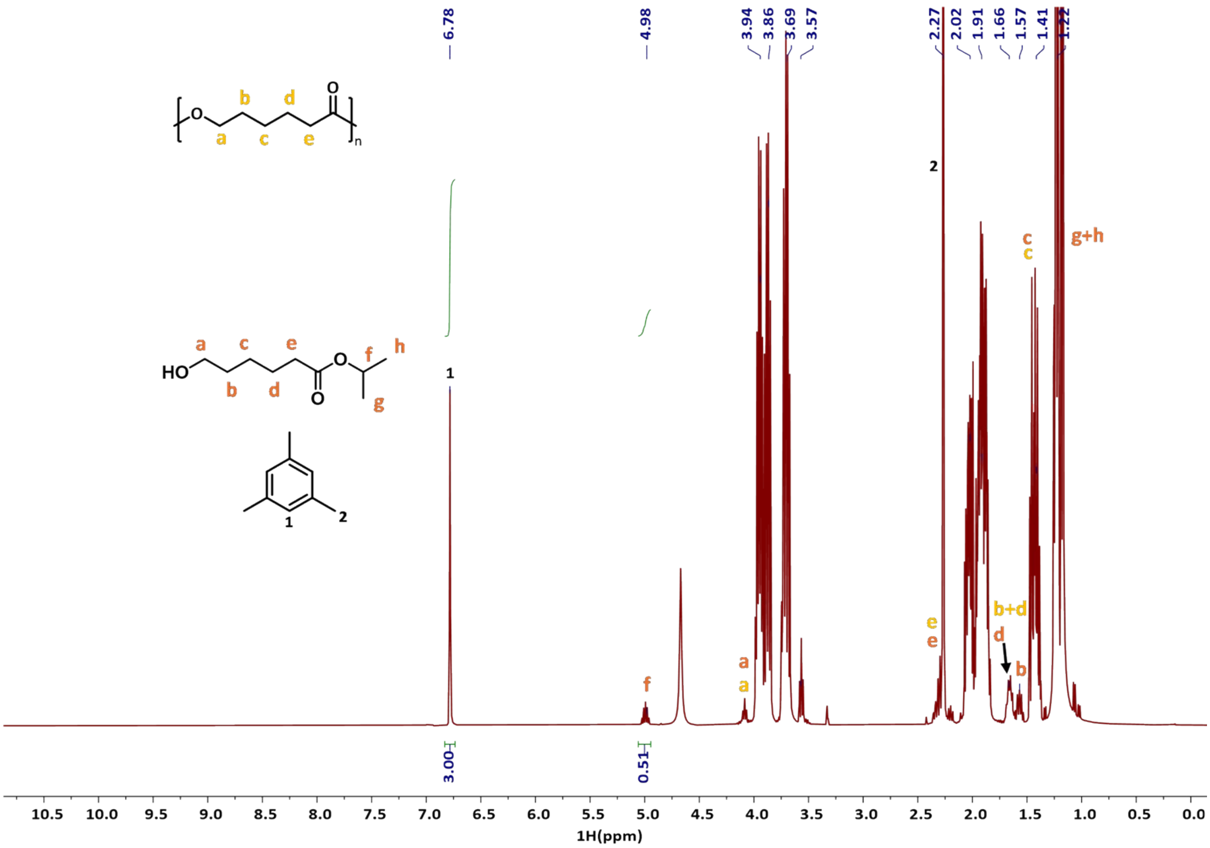

Figure S55. ^{1}H NMR (400 MHz, CD_3OD) spectrum of the reaction mixture resulting from the transesterification reaction done by using **isopropanol** as alcohol. Mesitylene is used as an internal standard.

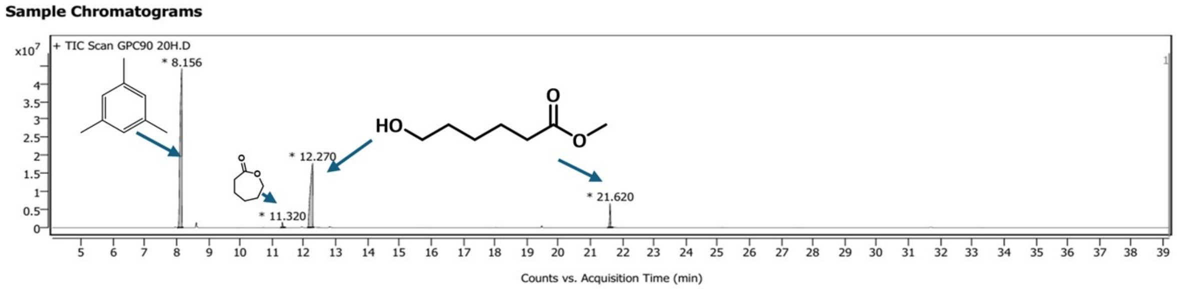

Chromatogram Peaks

Peak	Start	RT	End	Height	Area	Area %	SNR
1	8.013	8.156	8.207	44124982	171367763	100.00	
2	11.257	11.320	11.423	1440942	2519753	1.47	
3	12.104	12.270	12.321	17771985	95857098	55.94	
4	21.534	21.620	21.723	6682757	11447169	6.68	

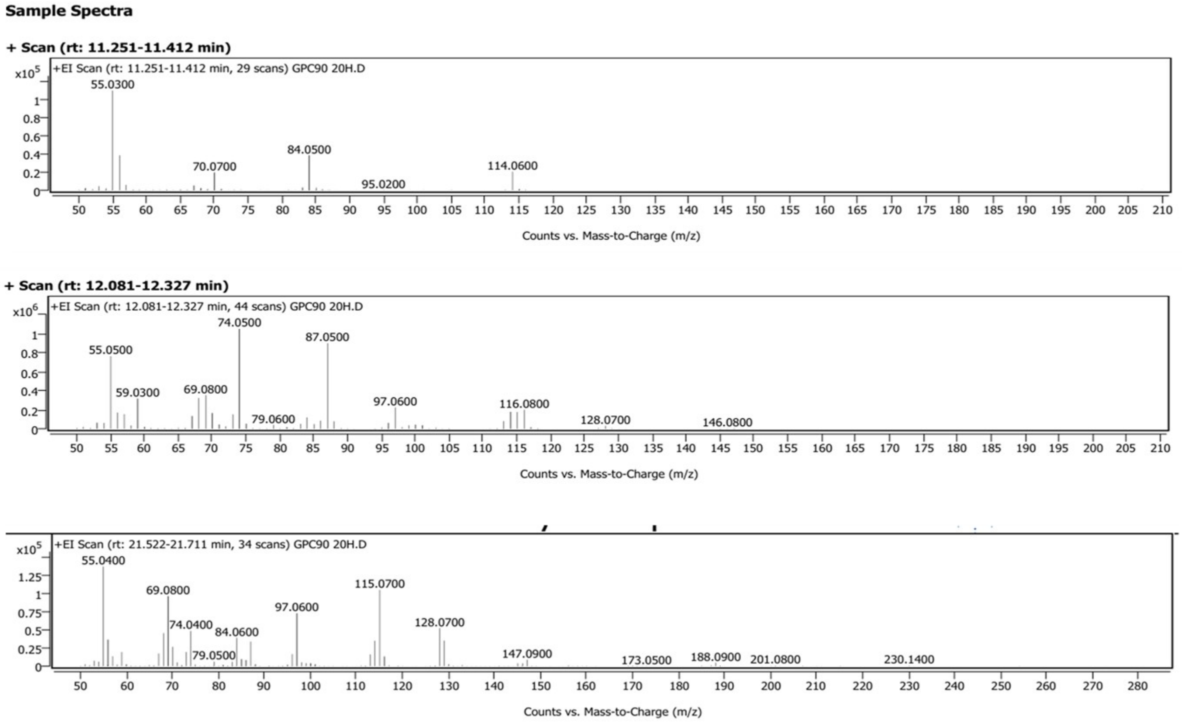

Figure S56. GC-MS data of the reaction mixture resulting from the transesterification reaction done by using **methanol** as alcohol. Mesitylene is used as an internal standard.

13. Role of temperature in transesterification and hydrogenation reactions

Conducting the transesterification reaction at 60 °C resulted in a 74% yield of E6-HH and a 3% yield of ε-CPL after 20 h (Figure S56). In contrast, hydrogenation of PCL under identical conditions as entry 17, Table S1(at 60 °C) produced 1,6-hexanediol selectively with 94% yield. Similarly, performing the transesterification reaction at 80 °C for 20 h afforded a 63% yield of E6-HH and a 0.5% yield of ε-CPL (Figure 4a), while the corresponding hydrogenation yielded >99% 1,6-hexanediol (entry 17, Table S1). These observations indicate that temperature plays a crucial role in the overall process: transesterification proceeds more efficiently at 60 °C, whereas hydrogenation is favoured at 80 °C.

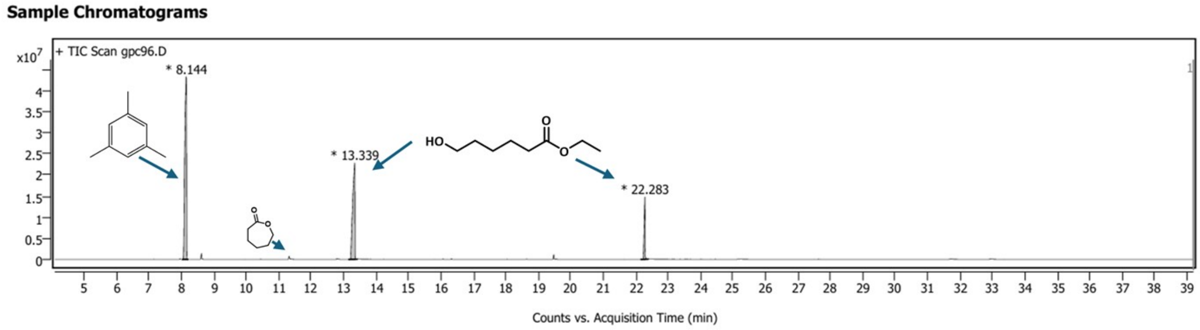

Chromatogram Peaks

Peak	Start	RT	End	Height	Area	Area %	SNR
1	8.018	8.144	8.207	43138966	160532911	100.00	
2	13.145	13.339	13.431	22806395	108812901	67.78	
3	22.151	22.283	22.397	14758835	29747868	18.53	

Figure S57. GC-MS data of the reaction mixture resulting from the transesterification reaction conducted at 60 ^oC. Mesitylene is used as an internal standard.

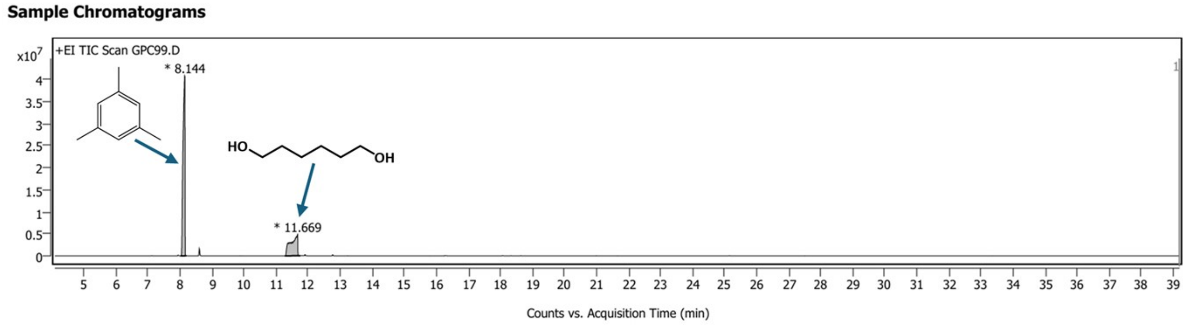

Chromatogram Peaks

Peak	Start	RT	End	Height	Area	Area %	SNR
1	8.018	8.144	8.196	40779080	169695004	100.00	
2	11.274	11.669	11.755	4797065	70821877	41.73	

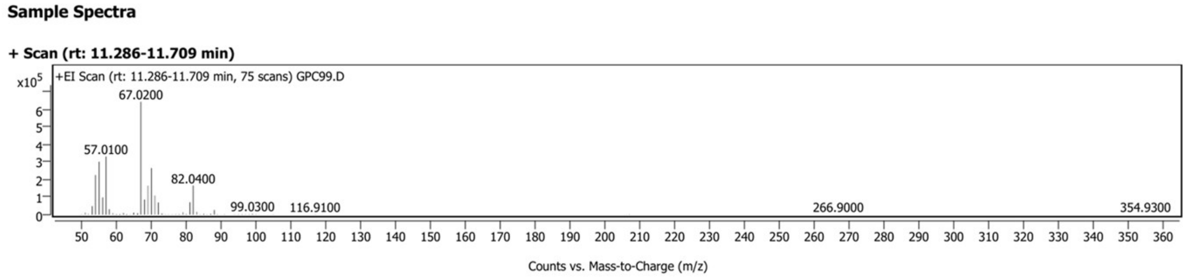

Figure S57a. GC-MS data of the reaction mixture resulting from the hydrogenation reaction conducted at 60 ^oC. Mesitylene is used as an internal standard.

14. Catalyst stability

To evaluate the catalyst stability, the reaction was first conducted for 72 h under the conditions described in entry 6, Table S2. Upon completion, 1 mmol of PCL was added to the reaction mixture, followed by recharging with H_2 gas, and the reaction was continued for an additional 72 h. However, no improvement in the product yield was observed, as confirmed by the NMR spectrum. The process predominantly resulted in depolymerization of the polymer into oligomers or E6-HH through a base-mediated transesterification pathway, indicating that the catalyst becomes deactivated after the first cycle.

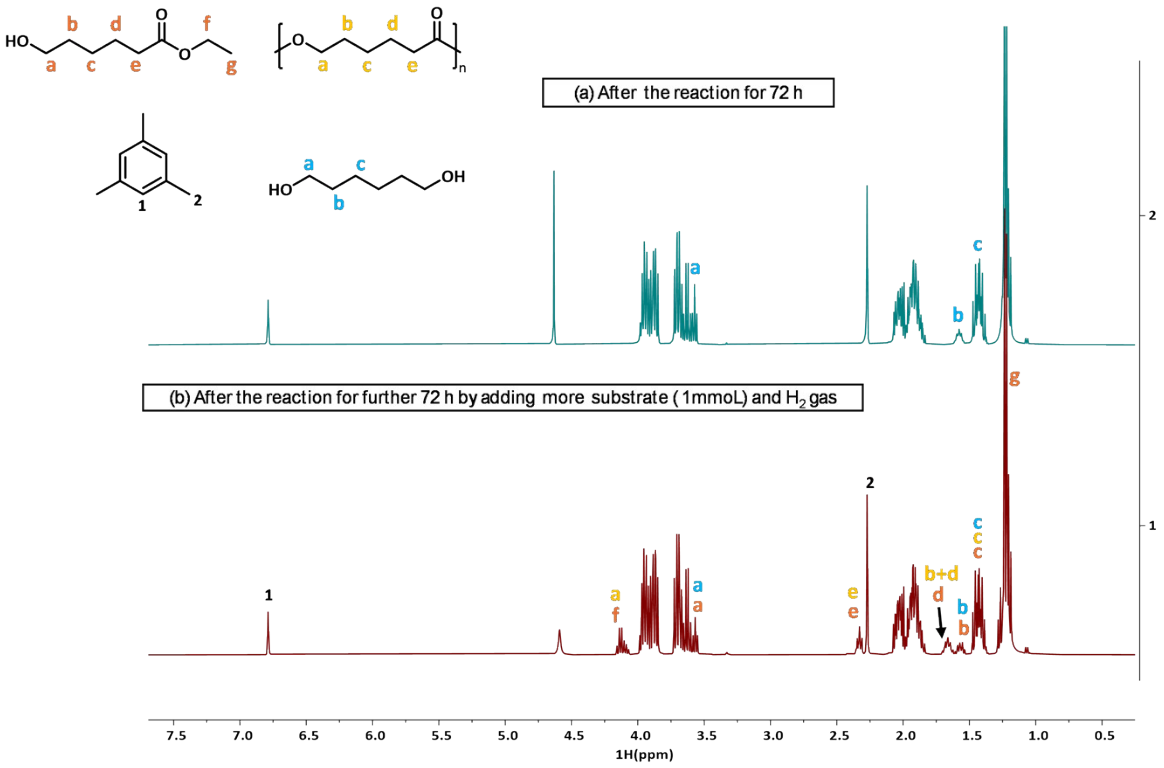

Figure S58. ^{1}H NMR (400 MHz, CD_3OD) spectrum of the reaction mixture resulting from the reaction corresponding to Table S2; Entry 6. Mesitylene is used as an internal standard.

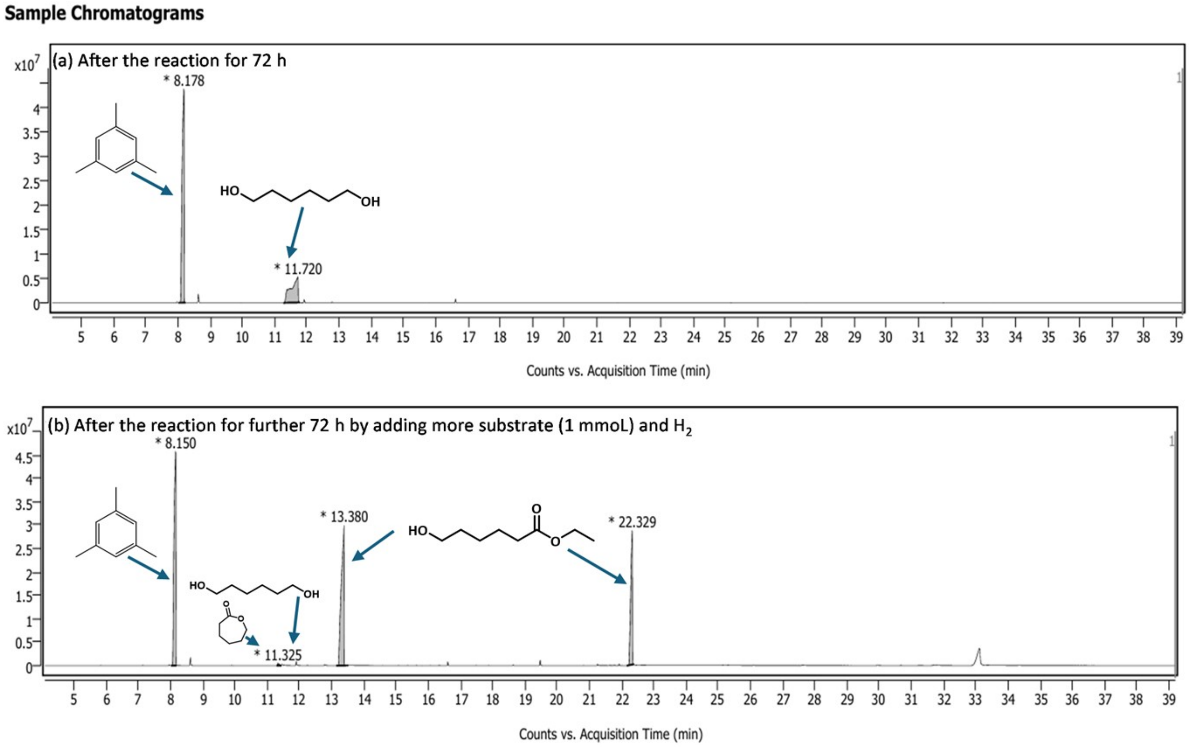

Figure S59. GC-MS data of the reaction mixture resulting from the reaction corresponding to Table S2; Entry 6. Mesitylene is used as an internal standard.

15. DFT Studies

The computational study was carried out using a two-step approach. Initially, geometry optimizations and frequency calculations were performed using the functional B3LYP[3] and the 6-21G basis set[4] to

obtain reliable starting structures. The optimized geometries and frequencies were then re-optimized at the higher B3LYP/6-311G+(d,p)[5] level of theory. Frequency calculations at this level of theory confirmed stationary points and transition states and were used to compute thermodynamic properties at 353.15 K, tetrahydrofuran (THF) as the reaction medium (using PCM as solvent model)[6] and 59.2 atm to match the experimental conditions. Gibbs Free Energies, unless otherwise stated, were computed by adding the Free Energy correction terms from the frequency calculations to the single point energies at the B3LYP/6-311G+(d,p) level of theory in solution (THF).

Gibbs free energies were computed by adding the free energy correction term from the frequency calculation to the single point energy in dioxane, according to

$$G^{B3LYP/6-311G*(d,p)}_{(THF,353.15K)} = E^{B3LYP/6-311G*(d,p)}_{THF} + corr^{B3LYP/6-311G*(d,p)}_{freq(THF,\ 59.2\ atm,\ 353.15K)}$$

Optimizations, frequency calculations and single point energies were done using the Gaussian 16 software suite in the C.01 revision.

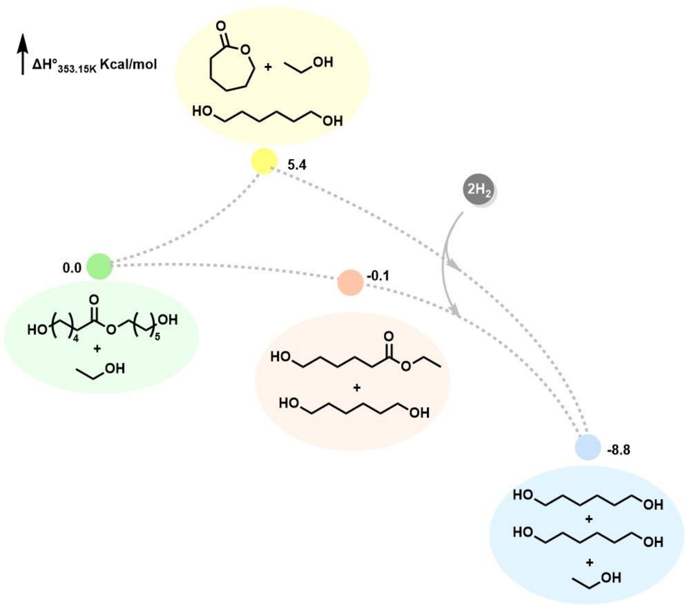

Figure S60. Enthalpy surface for the depolymerisation of caprolactone dimer to form 1,6-HD. Enthalpies were calculated at 353.15 K, 60 bar and at 1 M concentration of solutes in Kcal/mol.

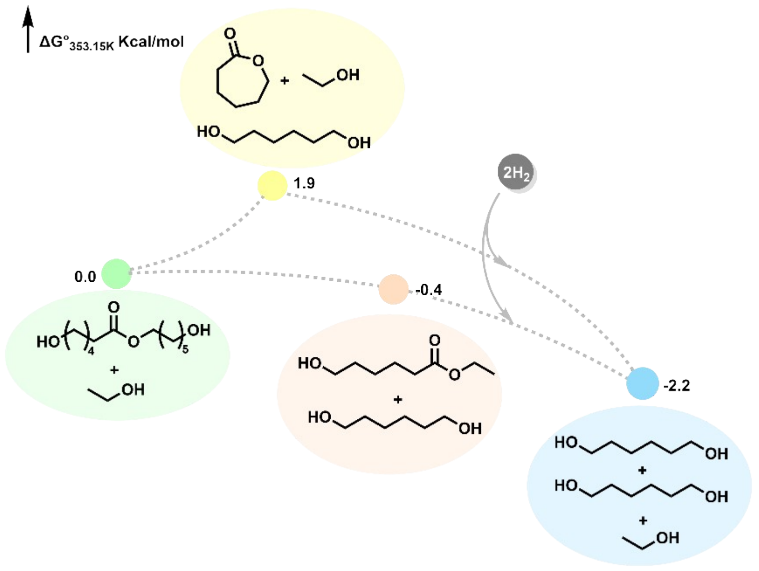

Figure S61. Energy surface for the depolymerisation of caprolactone dimer to form 1,6-HD. Free energies were calculated at 353.15 K, 60 bar and at 1 M concentration of solutes in Kcal/mol.

CARTESIAN COORDINATES

ε-Caprolactone

O	0.78044	-1.23389	-0.07345
O	2.50287	0.08419	-0.41033
C	-1.86520	0.67934	-0.03983
C	-0.62957	1.56425	-0.23517
C	-1.65614	-0.80020	-0.38100
C	0.59781	1.13832	0.60122
C	-0.56229	-1.49130	0.42411
C	1.37102	-0.02372	0.01131
H	-2.19427	0.75759	1.00398
H	-2.68367	1.07310	-0.65033
H	-0.88282	2.58866	0.05103
H	-0.35132	1.60090	-1.29430
H	-2.58776	-1.34369	-0.19088
H	-1.43531	-0.92714	-1.44682
H	0.28474	0.89194	1.62185
H	1.30848	1.96123	0.66911
H	-0.60918	-1.23192	1.48565
H	-0.64916	-2.57318	0.33714

Zero-point correction = 0.155869 Hartree/Particle
Thermal correction to Energy = 0.165754
Thermal correction to Enthalpy = 0.166872
Thermal correction to Gibbs Free Energy = 0.121449

Sum of electronic and zero-point Energies = -385.082609
Sum of electronic and thermal Energies = -385.072724
Sum of electronic and thermal Enthalpies = -385.071606
Sum of electronic and thermal Free Energies = -385.117028

Caprolactone Dimer

C -2.33804 -0.30459 -0.19039
H -2.29939 -0.78723 -1.17798
H -2.19214 -1.10975 0.54179
C -3.69312 0.38525 0.01825
H -3.81174 1.19096 -0.71766
H -3.71205 0.86211 1.00749
C -4.86911 -0.60124 -0.10049
H -4.73855 -1.41363 0.63056
H -4.85185 -1.07002 -1.09620
C -6.23343 0.07753 0.12524
H -6.37226 0.88646 -0.60499
H -6.25868 0.53739 1.12263
C -7.39545 -0.89912 0.00514
H -7.30597 -1.70501 0.74580
H -7.42848 -1.34532 -0.99776
O -8.63960 -0.14642 0.24807
H -9.42150 -0.72949 0.17870
O 0.03113 -0.05197 -0.14218
C 1.29805 0.73008 -0.07500
H 1.32300 1.40571 -0.93565
H 1.27880 1.32593 0.84247
C 2.44371 -0.27025 -0.09078
H 2.33542 -0.95459 0.76120
H 2.38103 -0.87738 -1.00361
C 3.81523 0.43056 -0.02377
H 3.92224 1.11371 -0.87951
H 3.86628 1.05228 0.88259
C 4.99069 -0.56454 -0.01955
H 4.87916 -1.25027 0.83396
H 4.94376 -1.18384 -0.92802
C 6.36095 0.13515 0.05818
H 6.48323 0.81025 -0.79992
H 6.41025 0.75459 0.96417
C 7.51989 -0.85225 0.07656
H 7.53174 -1.45836 -0.83909
H 7.44630 -1.52414 0.94211
O 8.76900 -0.07373 0.16576
H 9.54934 -0.66289 0.17899

O -1.21151 1.86674 0.00602
C -1.15772 0.62935 -0.09564

Zero-point correction = 0.353879 Hartree/Particle
Thermal correction to Energy = 0.381274
Thermal correction to Enthalpy = 0.382392
Thermal correction to Gibbs Free Energy = 0.285372

Sum of electronic and zero-point Energies = -772.534653
Sum of electronic and thermal Energies = -772.507257
Sum of electronic and thermal Enthalpies = -772.506139
Sum of electronic and thermal Free Energies = -772.603159

Ethanol

O -1.16167 -0.39642 0.00003
H -1.99194 0.09087 0.00015
C -0.07875 0.54789 -0.00002
H -0.14157 1.19009 -0.88696
H -0.14154 1.19016 0.88687
C 1.22707 -0.22326 -0.00002
H 1.30227 -0.85782 -0.88687
H 2.07392 0.46800 -0.00008
H 1.30232 -0.85772 0.88691

Zero-point correction = 0.079410 Hartree/Particle
Thermal correction to Energy = 0.085068
Thermal correction to Enthalpy = 0.086186
Thermal correction to Gibbs Free Energy = 0.052690

Sum of electronic and zero-point Energies = -155.020221
Sum of electronic and thermal Energies = -155.014563
Sum of electronic and thermal Enthalpies = -155.013445
Sum of electronic and thermal Free Energies = -155.046941

1,6 Hexanediol

C -1.92755 0.37825 0.00000
H -1.95681 1.03069 -0.88022
H -1.95680 1.03068 0.88023
C -0.62965 -0.43720 0.00000
H -0.61084 -1.09500 -0.87789
H -0.61084 -1.09500 0.87788

C 0.62965 0.43720 0.00000
H 0.61084 1.09500 0.87789
H 0.61084 1.09500 -0.87788
C 1.92755 -0.37825 0.00000
H 1.95680 -1.03068 -0.88023
H 1.95681 -1.03069 0.88022
C 3.17120 0.49641 0.00000
H 3.17929 1.14227 0.88746
H 3.17929 1.14227 -0.88746
C -3.17120 -0.49641 0.00000
H -3.17929 -1.14227 0.88746
H -3.17929 -1.14227 -0.88746
O 4.32752 -0.35539 -0.00001
H 5.11529 0.19822 0.00000
O -4.32752 0.35539 0.00000
H -5.11529 -0.19822 0.00001

Zero-point correction = 0.197890 Hartree/Particle
Thermal correction to Energy = 0.212206
Thermal correction to Enthalpy = 0.213324
Thermal correction to Gibbs Free Energy = 0.156206

Sum of electronic and zero-point Energies = -387.441300
Sum of electronic and thermal Energies = -387.426984
Sum of electronic and thermal Enthalpies = -387.425866
Sum of electronic and thermal Free Energies = -387.482984

Ethyl 6-hydroxyhexanoate

C 4.53364 -0.61149 -0.00007
H 4.55325 -1.25166 0.89203
H 4.55331 -1.25134 -0.89239
C 3.29850 0.27929 0.00004
H 3.33154 0.93163 0.88332
H 3.33156 0.93188 -0.88306
C 1.98911 -0.53207 -0.00008
H 1.96511 -1.18990 -0.88222
H 1.96510 -1.19014 0.88190
C 0.73785 0.36530 0.00003
H 0.75601 1.02231 -0.87953
H 0.75608 1.02218 0.87969
C -0.55804 -0.45675 0.00002
H -0.59707 -1.11890 -0.87619
H -0.59712 -1.11886 0.87627
C -1.81447 0.37767 -0.00003

O 5.71853 0.26584 0.00015
H 6.54362 -0.25888 -0.00024
O -1.86858 1.61930 0.00004
O -2.93926 -0.40574 -0.00018
C -4.27212 0.26659 -0.00016
H -4.32687 0.90004 0.88947
H -4.32709 0.89958 -0.89011
C -5.31826 -0.83102 0.00024
H -5.22572 -1.46249 0.88972
H -6.31860 -0.38275 0.00018
H -5.22585 -1.46300 -0.88890

Zero-point correction = 0.235610 Hartree/Particle
Thermal correction to Energy = 0.254105
Thermal correction to Enthalpy = 0.255223
Thermal correction to Gibbs Free Energy= 0.181343

Sum of electronic and zero-point Energies = -540.113454
Sum of electronic and thermal Energies = -540.094959
Sum of electronic and thermal Enthalpies = -540.093842
Sum of electronic and thermal Free Energies = -540.167722

16. References

1 S. Elangovan, C. Topf, S. Fischer, H. Jiao, A. Spannenberg, W. Baumann, R. Ludwig, K. Junge and M. Beller, *J Am Chem Soc*, 2016, **138**, 8809–8814.

2 C. Alberti and S. Enthaler, *ChemistrySelect*, 2021, **6**, 11244–11248.

3 A. D. Becke, *J Chem Phys*, 1993, **98**, 5648–5652.

4 J. S. Binkley, J. A. Pople and W. J. Hehre, *J Am Chem Soc*, 1980, **102**, 939–947.

5 R. Krishnan, J. S. Binkley, R. Seeger and J. A. Pople, *J Chem Phys*, 1980, **72**, 650–654.

6 J. Tomasi, B. Mennucci and R. Cammi, *Chem Rev*, 2005, **105**, 2999–3093.
